# Long-Term Use of Everolimus in Patients with Tuberous Sclerosis Complex: Final Results from the EXIST-1 Study

**DOI:** 10.1371/journal.pone.0158476

**Published:** 2016-06-28

**Authors:** David N. Franz, Elena Belousova, Steven Sparagana, E. Martina Bebin, Michael D. Frost, Rachel Kuperman, Olaf Witt, Michael H. Kohrman, J. Robert Flamini, Joyce Y. Wu, Paolo Curatolo, Petrus J. de Vries, Noah Berkowitz, Julie Niolat, Sergiusz Jóźwiak

**Affiliations:** 1 Department of Neurology, Cincinnati Children’s Hospital Medical Center, Cincinnati, Ohio, United States of America; 2 Department of Pediatrics, Research and Clinical Institute of Pediatrics, Pirogov Russian National Research Medical University, Moscow, Russia; 3 Department of Neurology, Texas Scottish Rite Hospital for Children, Dallas, Texas, United States of America; 4 Department of Neurology, University of Alabama School of Medicine, Birmingham, Alabama, United States of America; 5 Department of Neurology, Minnesota Epilepsy Group, St. Paul, Minnesota, United States of America; 6 Department of Neurology, Children’s Hospital and Research Center, Oakland, California, United States of America; 7 Department of Oncology, University of Heidelberg Medical Center and German Cancer Research Center, Heidelberg, Germany; 8 Department of Neurology, University of Chicago, Chicago, Illinois, United States of America; 9 Department of Neurology, Children’s Healthcare of Atlanta, Atlanta, Georgia, United States of America; 10 Department of Neurology, Mattel Children’s Hospital at the University of California Los Angeles, Los Angeles, California, United States of America; 11 Department of Neurology, University of Rome Tor Vergata, Rome, Italy; 12 Department of Psychiatry and Mental Health, Division of Child & Adolescent Psychiatry, University of Cape Town, Cape Town, South Africa; 13 Department of Oncology, Novartis Pharmaceuticals Corporation, East Hanover, New Jersey, United States of America; 14 Department of Oncology, Novartis Pharmaceuticals S.A.S., Rueil-Malmaison, France; 15 Department of Pediatric Neurology, Medical University of Warsaw, Warsaw, Poland; Institute of Bioengineering and Nanotechnology, SINGAPORE

## Abstract

**Background:**

Everolimus, a mammalian target of rapamycin (mTOR) inhibitor, has demonstrated efficacy in treating subependymal giant cell astrocytomas (SEGAs) and other manifestations of tuberous sclerosis complex (TSC). However, long-term use of mTOR inhibitors might be necessary. This analysis explored long-term efficacy and safety of everolimus from the conclusion of the EXIST-1 study (NCT00789828).

**Methods and Findings:**

EXIST-1 was an international, prospective, double-blind, placebo-controlled phase 3 trial examining everolimus in patients with new or growing TSC-related SEGA. After a double-blind core phase, all remaining patients could receive everolimus in a long-term, open-label extension. Everolimus was initiated at a dose (4.5 mg/m^2^/day) titrated to a target blood trough of 5–15 ng/mL. SEGA response rate (primary end point) was defined as the proportion of patients achieving confirmed ≥50% reduction in the sum volume of target SEGA lesions from baseline in the absence of worsening nontarget SEGA lesions, new target SEGA lesions, and new or worsening hydrocephalus. Of 111 patients (median age, 9.5 years) who received ≥1 dose of everolimus (median duration, 47.1 months), 57.7% (95% confidence interval [CI], 47.9–67.0) achieved SEGA response. Of 41 patients with target renal angiomyolipomas at baseline, 30 (73.2%) achieved renal angiomyolipoma response. In 105 patients with ≥1 skin lesion at baseline, skin lesion response rate was 58.1%. Incidence of adverse events (AEs) was comparable with that of previous reports, and occurrence of emergent AEs generally decreased over time. The most common AEs (≥30% incidence) suspected to be treatment-related were stomatitis (43.2%) and mouth ulceration (32.4%).

**Conclusions:**

Everolimus use led to sustained reduction in tumor volume, and new responses were observed for SEGA and renal angiomyolipoma from the blinded core phase of the study. These findings support the hypothesis that everolimus can safely reverse multisystem manifestations of TSC in a significant proportion of patients.

**Trial Registration:**

ClinicalTrials.gov NCT00789828

## Introduction

Tuberous sclerosis complex (TSC) is a genetic disorder that occurs in approximately 1:6000 live births affecting approximately1 million people worldwide [1,2]. In TSC, the growth of benign tumors in various organs occurs from loss of *TSC1* or *TSC2* genes and subsequent overactivation of mammalian target of rapamycin (mTOR), a kinase responsible for regulating cell growth, proliferation, and angiogenesis [1,3,4].

Subependymal giant cell astrocytomas (SEGAs) are slow-growing tumors often found near the foramen of Monro in the brains of patients with TSC [5–8]. SEGA growth can impede cerebrospinal fluid flow in the ventricles, leading to acute hydrocephalus or death [4,7]. In the kidneys, renal angiomyolipomas, which are tumors composed of fat cells, immature smooth muscle, and abnormal blood vessels, grow in number and size with age [9,10]. Larger angiomyolipomas can develop aneurysms that can rupture and cause life-threatening hemorrhage or encroach on normal renal tissue, leading to renal failure [7]. Skin lesions, such as hypomelanotic macules, facial angiofibromas, and shagreen patches, can also be present at birth or develop early in life in the majority of patients and can cause significant physical and psychological burden [11].

Everolimus has demonstrated efficacy in treating symptomatic, growing SEGAs in patients with TSC, first in an open-label phase 1/2 trial in 28 patients with TSC-associated SEGA [12] and subsequently in the double-blind phase in the large randomized, international, placebo-controlled, phase 3 trial EXIST-1 [13]. Everolimus has also demonstrated significant reduction in renal angiomyolipoma volume compared with placebo in the phase 3 EXIST-2 trial [14]. Consensus guidelines now include mTOR inhibitors as recommended treatment for asymptomatic, growing SEGAs and renal angiomyolipomas [15–17]. In addition, mTOR inhibitors have shown promise in treating multiple manifestations of TSC, including skin manifestations, cardiac rhabdomyoma, pulmonary lymphangioleiomyomatosis, and epilepsy, which supports the use of mTOR inhibitors as targeted multisystemic therapy for the disease [18,19].

There is some evidence that TSC-associated tumors regrow after cessation of mTOR inhibitor treatment, suggesting that therapy with an mTOR inhibitor might necessitate long-term or perhaps indefinite use [20]. Therefore, EXIST-1 included a long-term, open-label extension phase to assess the efficacy and safety of everolimus over at least 4 years [13]. Interim results from 111 patients treated with everolimus showed sustained SEGA reduction and acceptable toxicity over approximately 2 years of treatment [21]. The EXIST-1 study concluded on October 2, 2014, and final results from approximately 4 years of treatment with everolimus in terms of the primary efficacy end point (SEGA), as well as secondary (skin lesions), and exploratory (renal angiomyolipoma) efficacy and safety end points, are presented in this article.

## Methods

### Study Design and Participants

The methodology of this study has been published previously [13,21]. This prospective, multicenter, double-blind, placebo-controlled phase 3 trial included patients of any age with definitive TSC per modified Gomez criteria [22,23] with ≥1 target SEGA lesion (≥1.0 cm in longest diameter using magnetic resonance imaging [MRI]) and radiological evidence of serial SEGA growth, presence of a new SEGA lesion, or new or worsening hydrocephalus. The study was initiated on August 20, 2009 (first patient randomly assigned) and follow-up continued until October 2, 2014.

The study was reviewed by an independent ethics committee or institutional review board for each center (see S1 Table for list of committees/review boards) and was conducted in compliance with good clinical practice and under the principles of the Declaration of Helsinki. Written informed consent was obtained from each patient or the patient’s guardian prior to randomization. The protocol of this trial and supporting CONSORT checklist are available as supporting information (see S1 Protocol and S1 Checklist).

### Procedures

In the core phase, patients were randomly assigned 2:1 to receive either everolimus or placebo until the last patient randomly assigned had been treated for 6 months [13]. After positive results were observed in the double-blind core phase (database cutoff, March 2, 2011), the study continued in a preplanned extension phase in which all patients remaining in the study at the end of the core phase could receive open-label everolimus until the last patient randomly assigned had been treated for 4 years.

Everolimus was initiated at a dose of 4.5 mg/m^2^/day based on body surface area and titrated to blood trough concentrations of 5–15 ng/mL, with adjustments allowed based on toxicity. Trough concentrations were monitored at each visit. Brain MRI and kidney computed tomography /MRI (for patients with ≥1 target renal angiomyolipoma, ≥1.0 cm in longest diameter) were performed at screening/baseline, at weeks 12, 24, and 48 after treatment initiation, and annually thereafter. Physician’s global assessment (PGA) of skin lesions was performed every 12 weeks. Sexual maturation was assessed at screening/baseline and annually thereafter using Tanner staging and developmental milestones. For patients aged <18 years at treatment initiation, height and weight were measured at 24-week intervals before and after starting everolimus. Patients who discontinued study medication received an end-of-study treatment evaluation 28 days after their last everolimus dose.

### End Points

The primary end point—SEGA response rate—represented the proportion of patients with ≥50% reduction in the sum volumes of all target SEGA lesions from baseline in the absence of worsening nontarget or new target SEGA lesions and new or worsening hydrocephalus per central radiology review. Secondary efficacy end points included time to SEGA response, time to SEGA progression, and duration of SEGA response. SEGA progression was defined as an increase in SEGA volume ≥25% from lowest value to a value greater than baseline or as appearance of new SEGA lesions ≥1 cm in longest diameter, worsening of nontarget lesions, or new or worsening hydrocephalus. The duration of SEGA response was defined as the duration between first SEGA response and the time of progression or last radiological assessment.

Renal angiomyolipoma response rate was an exploratory end point. This was defined as the proportion of patients with ≥50% reduction in the sum volume of all target renal angiomyolipomas compared with baseline in the absence of any new target renal angiomyolipomas, as >20% increase in volume in either kidney from its lowest value, or as renal angiomyolipoma-related bleed of grade ≥2 per central radiology review. Skin lesion response rate was defined as the proportion of patients with either complete clinical response (100% improvement) or partial response (≥50% improvement) in skin lesions according to the PGA per the investigator. Skin lesion types included in the assessment were hypomelanotic macules, shagreen patches, periungual or subungual fibroma, facial angiofibroma, and forehead plaques. Duration of skin lesion response was defined as the time from the date of first skin lesion response until progression (worsening of the lesion by ≥25% compared with baseline), according to the PGA.

All responses required confirmation with a second scan (PGA for skin lesions) at least 8 weeks later. Adverse events (AEs) were assessed according to the National Cancer Institute Common Toxicity Criteria for Adverse Events version 3.0.

### Statistical Analysis

A detailed discussion of the methodology has been described previously [13,21]. The current analyses included all patients who received ≥1 dose of everolimus. Descriptive statistics were used to summarize baseline data at everolimus initiation. Changes of volume from baseline with exact 95% confidence intervals (CIs) were calculated for SEGA response and renal angiomyolipoma response. The 95% CIs were also calculated for skin lesion response rate. Summary statistics from the Kaplan-Meier curve determined median duration of SEGA response and median time to SEGA progression, with statistics given as point estimates with 95% CI. Height and weight were summarized using standard deviation scores (SDSs), taking into account the Centers for Disease Control and Prevention (CDC) growth charts. Data from patients who discontinued the study prematurely were included in these analyses up until the time of their discontinuation.

## Results

### Patients

**Fig 1:**
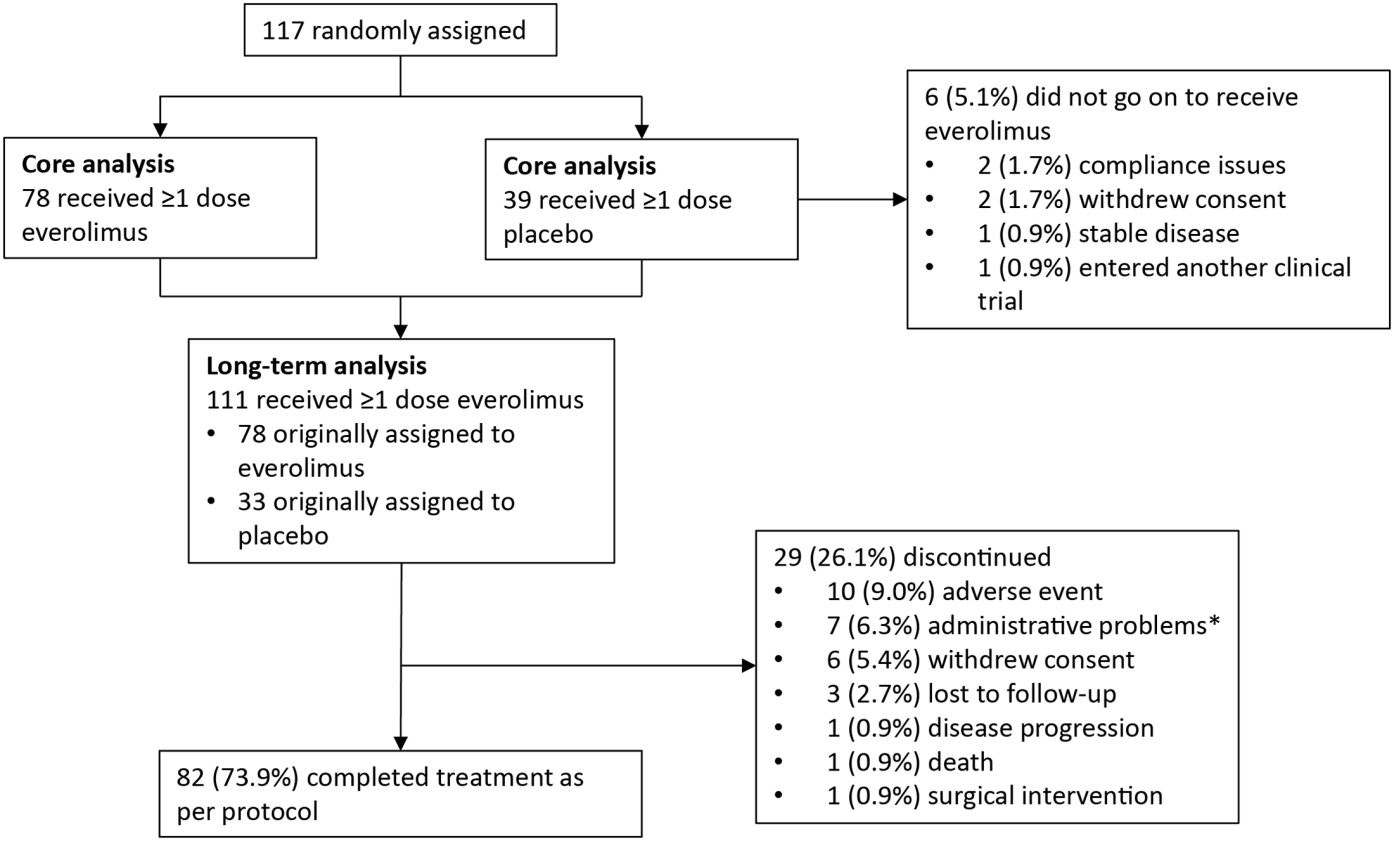
CONSORT diagram. *Administrative problems defined as non-compliant with study visit or non-compliant with study drug.

Patient baseline characteristics have been reported previously [13,21]. Overall, 111 patients (33 originally assigned to receive placebo) received ≥1 dose of everolimus and were included in this final analysis (Fig 1). The majority of patients (83.8%) were aged <18 years (Table 1). At baseline (everolimus initiation), all patients (100%) had evidence of SEGA, 71 (64%) had renal angiomyolipoma as a major feature (41 [36.9%] had ≥1 target renal angiomyolipoma), and 105 (94.6%) had ≥1 skin lesion. At the end of the study (October 2, 2014), median duration of everolimus exposure was 47.1 months (range, 1.9–58.3); median dose intensity was 5.89 mg/m^2^/day (range, 1.0–13.8).

**Table 1:** Patient Baseline Characteristics.

	Everolimus (N = 111)
Median age (range), years	9.5 (1.1–27.4)
Age category, n (%)	
<3 years	18 (16.2)
3 to <10 years	41 (36.9)
10 to <18 years	34 (30.6)
≥18 years	18 (16.2)
Male sex, n (%)	64 (57.7)
Median body surface area (range), m^2^	1.07 (0.4–2.2)
Race, n (%)	
White	104 (93.7)
Black	4 (3.6)
Pacific Islander	1 (0.9)
Other	2 (1.8)
Use of EIAED at start of everolimus, n (%)	43 (38.7)
TSC feature, n (%)	
SEGA	111 (100)
Renal angiomyolipoma (major feature)	71 (64)
Renal angiomyolipoma (≥1 target lesion)	41 (36.9)
≥1 skin lesion	105 (94.6)
Median sum volume of target SEGA lesions (range), cm^3^	1.58 (0.2–25.2)
No. of target SEGA lesions, n (%)	
0	2 (1.8)
1	60 (54.1)
2	46 (41.4)
3	2 (1.8)
≥4	1 (0.9)
Median sum volume of target renal angiomyolipoma lesions (range), cm^3^	10.03 (0.5–198.1)*
No. of target renal angiomyolipoma lesions, n (%)	
1–5	33 (29.7)
6–10	8 (7.2)
>10	0

EIAED, enzyme-inducing antiepileptic drug; SEGA, subependymal giant cell astrocytoma; TSC, tuberous sclerosis complex.

*Calculated in patients with ≥1 target renal angiomyolipoma lesion (≥1.0 cm in longest diameter) and a readable scan (n = 38).

### SEGA-Related Results

At study completion, SEGA response had been achieved at any time by 64 patients, for a response rate of 57.7% (95% CI, 47.9–67.0) per central radiology review. This is an increase in best overall response from 34.6% in the primary analysis and 48.6% from an approximately 2-year interim analysis [13,21]. Median time to SEGA response was 5.32 months (95% CI, 3.02–5.59) and ranged from approximately 2.5 to 33.1 months. Although the majority of responses were observed within the first months, some responses were observed/confirmed more than 2.5 years into the study.

**Fig 2:**
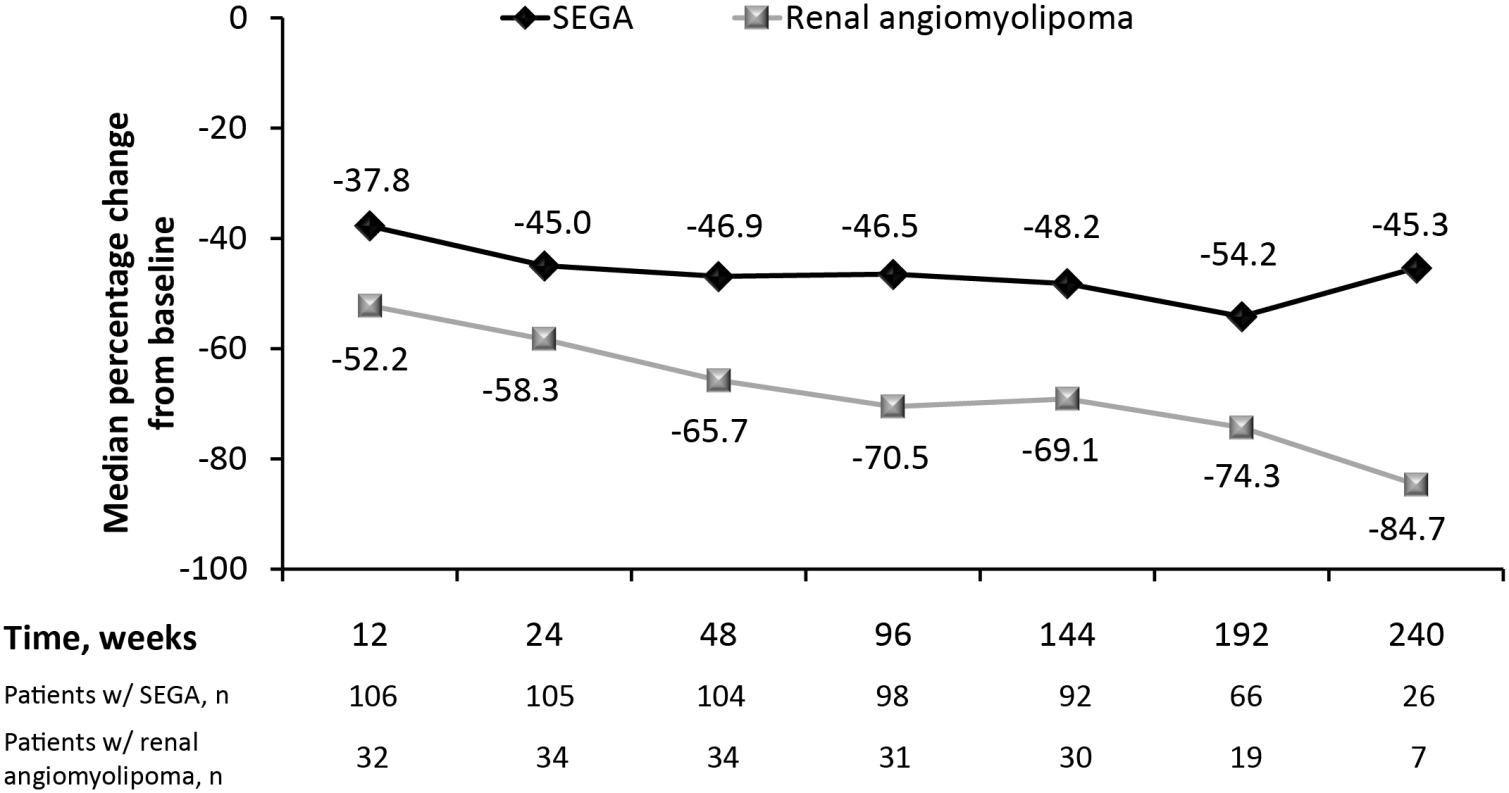
Median percentage reduction in SEGA and renal angiomyolipoma volume over time.

**Fig 3:**
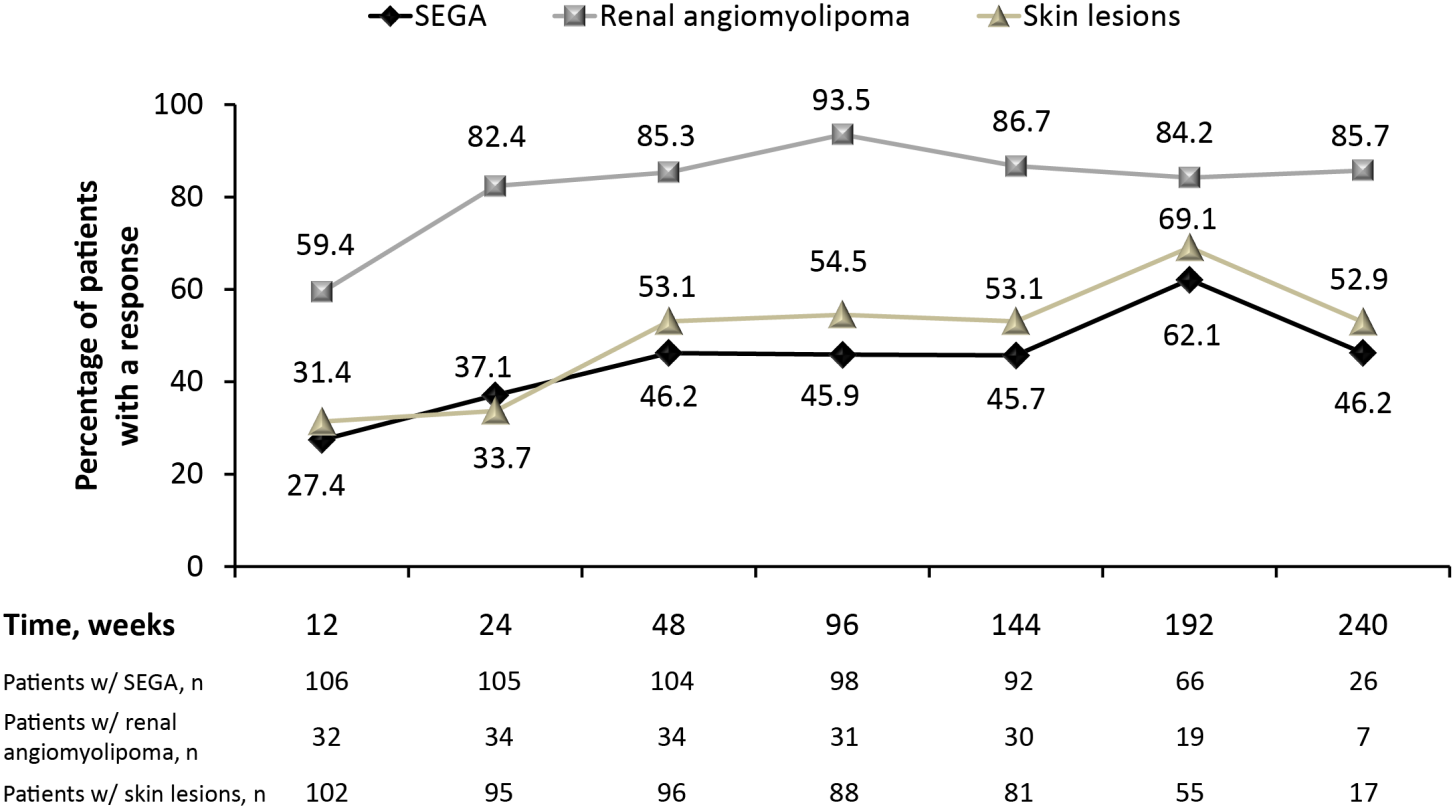
Proportion of patients with ≥50% reduction/improvement in SEGA, renal angiomyolipoma, or skin lesions. * *Skin lesion response determined as partial or complete by Physician’s Global Assessment.

Median reduction from baseline in total SEGA volume increased slightly over time (Fig 2), as did the proportion of patients achieving ≥50% reduction in SEGA volume over time (Fig 3). The number of patients with values at week 240 was low (26 of 111); however, after 192 weeks of treatment, 62% of patients (41 of 66) had ≥50% reduction in SEGA volume and 77% of patients (51 of 66) had ≥30% reduction in SEGA volume.

Thirteen patients (11.7%) had SEGA progression at any time during the study. Only one of these patients discontinued everolimus treatment because of SEGA progression. For six patients, progression was detected at the end-of-treatment visit after everolimus had already been discontinued for other reasons (completed treatment [n = 2], AE [n = 2] noncompliance [n = 1], withdrew consent [n = 1]). In the other six patients, although progression was noted, everolimus was continued at the discretion of the investigator. SEGA progression occurred approximately 250 to 1700 days after everolimus initiation. Five of the 13 patients with progression had achieved response before progression. Duration of SEGA response for all responders (duration between first SEGA response and the time of progression *or* last radiological assessment) ranged from approximately 5.1 to 53.3 months. The Kaplan-Meier estimate of duration of response at 48 months was 89.4% (95% CI, 76.2–95.5). Because of the low number of SEGA progressions, median time to SEGA progression could not be determined. The progression-free survival rate at 3 years after treatment initiation was 88.8% (95% CI, 80.6–93.6). No patient required surgery because of SEGA progression during the study.

### Renal Angiomyolipoma Response

Of the 41 patients with ≥1 target renal angiomyolipoma at baseline, 30 patients achieved response, for a response rate of 73.2% (95% CI, 57.1–85.8). This represents an improvement from 53.3% (n = 16 of 30) in the everolimus arm at the time of the primary analysis [13]. The median duration of renal angiomyolipoma response was 42.3 months (95% CI, 27.8–NA). Renal angiomyolipoma volume continued to decrease over the study period (Fig 2); at week 192 (n = 19), median percentage reduction in the sum volume of target angiomyolipoma lesions was 74.3%, with a median volume decrease from 14.65 cm^3^ at baseline to 4.35 cm^3^.

Nearly 95% of evaluable patients experienced a reduction in the sum volume of target angiomyolipoma lesions as the best percentage change per central radiology review. More than 80% of patients experienced reduction in renal angiomyolipoma volume of ≥50% at week 24, and this degree of response continued over the study period (Fig 3). No new angiomyolipoma lesions were observed, and no instance of grade ≥2 bleeding occurred.

### Skin Lesion Response

In 105 patients with ≥1 skin lesion at baseline, the skin lesion response rate was 58.1% (61 of 105; 95% CI, 48.1–67.7), with nine patients (8.6%) experiencing complete clinical response and 52 (49.5%) experiencing partial response. This was improved from a partial response rate of 42% (30 of 72) and complete response rate of 0% reported among everolimus-treated patients during the primary analysis of the study [13]. More than half the patients demonstrated a response to treatment; nearly one-third reached complete or partial response as early as week 12, with responder rates increasing steadily through week 192 (Fig 3). Three of the 61 responders (4.9%) experienced progression of skin lesions, ranging from 2.3 to 55.5 months after the response was first noted.

### Safety

All but one patient (99.1%) experienced an AE during this long-term study. Emergence of individual AEs was generally highest in the first year and decreased each subsequent year (Table 2). The majority of patients (89.2%) experienced ≥1 AE that was suspected to be related to everolimus. The most common AEs (by preferred term) suspected to be treatment-related in >10% of patients were stomatitis (43.2%), mouth ulceration (32.4%), pneumonia (13.5%), blood cholesterol level increase (11.7%), hypercholesterolemia (11.7%), nasopharyngitis (10.8%), and pyrexia (10.8%). Among all patients, 36.0% experienced a grade 3 treatment-related AE. The most common (>5%) events of this severity were stomatitis (10.8%), pneumonia (8.1%), and neutropenia (5.4%). Only five patients (4.5%) experienced a treatment-related grade 4 AE, which included neutropenia (2 patients, 1.8%), pneumonia, febrile infection, gastroenteritis, and pneumothorax (1 patient each, 0.9%); febrile infection and gastroenteritis occurred in the same patient. Most patients (n = 101 [91.0%]) required ≥1 dose interruption or reduction during the study. The most common reason for dose interruption or reduction was an AE (n = 80 [72.1%]).

**Table 2:** AEs by Preferred Term Regardless of Relationship to Study Drug and by Year of Emergence Occurring in ˃10% of Patients.

	Everolimus				
AEs, n (%)	≤12 months (N = 111)	13–24 months (n = 106)	25–36 months (n = 98)	37–48 months (n = 88)	>48 months (n = 57)
Any AE	108 (97.3)	93 (87.7)	84 (85.7)	66 (75.0)	28 (49.1)
Stomatitis	44 (39.6)	13 (12.3)	11 (11.2)	6 (6.8)	5 (8.8)
Mouth ulceration	32 (28.8)	15 (14.2)	10 (10.2)	7 (8.0)	1 (1.8)
Convulsion	24 (21.6)	15 (14.2)	13 (13.3)	10 (11.4)	4 (7.0)
Pyrexia	22 (19.8)	18 (17.0)	12 (12.2)	5 (5.7)	1 (1.8)
Vomiting	21 (18.9)	8 (7.5)	5 (5.1)	3 (3.4)	0
Cough	21 (18.9)	7 (6.6)	6 (6.1)	4 (4.5)	2 (3.5)
Nasopharyngitis	19 (17.1)	12 (11.3)	10 (10.2)	9 (10.2)	2 (3.5)
Diarrhea	18 (16.2)	9 (8.5)	3 (3.1)	2 (2.3)	3 (5.3)
Upper respiratory tract infection	16 (14.4)	9 (8.5)	4 (4.1)	6 (6.8)	1 (1.8)
Pharyngitis	13 (11.7)	5 (4.7)	8 (8.2)	4 (4.5)	4 (7.0)
Ear infection	12 (10.8)	5 (4.7)	6 (6.1)	1 (1.1)	0
Pneumonia	7 (6.3)	12 (11.3)	10 (10.2)	3 (3.4)	2 (3.5)

AE, adverse event.

In total, 11 patients (9.9%) experienced an AE that led to discontinuation. These AEs included *Acinetobacter* bacteremia, aggression, anemia, azoospermia, blood alkaline phosphatase level increase, focal segmental glomerulosclerosis, need for neurosurgery, neutropenia, pneumonia, pneumothorax, sinusitis, stomatitis, and viral infection (1 patient each, 0.9%). Among these, viral infection, blood alkaline phosphatase level increase, and *Acinetobacter* bacteremia were all reported in the same patient. One patient experienced an AE that led to discontinuation, but the investigator confirmed that the main reason for treatment discontinuation was withdrawal of consent by the subject. One death (accidental asphyxiation) was reported and was not suspected by the investigator to be treatment related.

### Growth and Development and Sexual Maturation

More than half the patients were at Tanner stage 1 at baseline (34 of 64 male patients, 27 of 47 female patients for breast development, and 25 of 47 female patients for pubic hair). Over the course of the study, normal progression in Tanner stage was usually seen. Median age to attain Tanner stage 2 was 12.1 years for genitalia and 12.0 years for pubic hair in males (range, 9.5–14.9) and 10.4 years for breast development and 11.0 years for pubic hair in females (range, 6.6–13.0).

In the 31 female patients between the ages of 10 and 55 years who were at risk for amenorrhea, seven (22.6%) experienced ≥1 amenorrhea episode. Three patients reported grade 3 amenorrhea; two were suspected to be related to study drug. None of these events led to study discontinuation or necessitated dose adjustment.

Of the 93 patients aged <18 years at everolimus initiation with an evaluable height and weight SDS at baseline, six (6.5%) and 13 (14.0%) reported notably low height and weight SDS at baseline, respectively, and 16 (17.2%) and 15 (16.1%) reported notably high height and weight SDS at baseline. These percentages did not increase over time after start of everolimus.

## Discussion

The results from this final analysis of the long-term EXIST-1 study add to the compelling evidence for the efficacy and safety of everolimus in patients with TSC-associated SEGA. The core phase of this study showed the superiority of everolimus compared with placebo in terms of SEGA response rate [13]; however, the core phase duration was inadequate to assess the long-term implications of everolimus treatment in these patients. In this final analysis, SEGA response rate increased to 58% over approximately 4 years of treatment, indicating that patients who do not initially respond to everolimus might achieve response with continued exposure. The proportion of patients with a clinically significant ≥50% reduction in SEGA volume generally increased over time, showing that clinically relevant SEGA reductions persisted. The rate of SEGA progression was relatively low (~12%), and a number of patients continued everolimus despite progression because they were attaining clinical benefit.

Evaluation of SEGA was the primary focus of this study. However, because TSC affects multiple organ systems, we also evaluated the effects of everolimus in patients with renal angiomyolipoma and skin lesions. In this analysis, increasing and sustained reductions in renal angiomyolipoma volume and improvement in skin lesions were observed, providing evidence of broad clinical impact of systemic treatment with everolimus in patients with TSC. Although the study was not adequately designed to fully assess effects in these manifestations, the positive results are not entirely unexpected. The evidence examining use of oral mTOR inhibitors for TSC has shown that the drug class has the potential for multisystemic effects in TSC not only in treating SEGAs or renal angiomyolipomas, but also TSC-associated seizures, cardiac rhabdomyoma, lymphangioleiomyomatosis, or some of the TSC-associated neuropsychiatric disorders [18,19]. Additional studies in these areas are needed or are underway.

Everolimus proved to be safe and well tolerated over the long term. The pattern of AEs was consistent with the known safety profile of everolimus. Although more than 80% of patients experienced an AE suspected to be drug related during treatment, most AEs resolved without any complication. Nearly three-quarters of the patients completed this long-term study as per protocol, with only 10 patients (9.0%) discontinuing the study drug because of an AE.

The frequency of emerging AEs of all types decreased over time, with stomatitis and mouth ulceration remaining the most common. Lack of data on the long-term effects of mTOR inhibitors on growth and sexual maturation had represented an area of uncertainty, but clinical data from this report do not indicate that these areas are impacted by use of everolimus. This is further supported by data in pediatric transplant patients, in whom the use of everolimus for up to 11 years was not associated with growth disturbances and did not impact sexual maturation [24,25].

The goal of treatment with an mTOR inhibitor is to decrease or stabilize the size and number of tumors in patients with TSC [15,16,26]. In this long-term follow-up of patients with growing SEGA at baseline, no patient required surgery because of SEGA progression while on everolimus, and one patient elected to discontinue from the study to pursue surgery despite having >50% reduction in SEGA volume. For one patient there was radiographic evidence of hydrocephalus in the absence of SEGA growth and without clinical symptoms of progression. None of the patients with renal angiomyolipoma experienced renal hemorrhage.

Similar to long-term phase 1/2 data, at least half the patients had SEGA volume reduction ≥50% at later time points (192 weeks [44 months] in this study and at 48 and 60 months in the phase 1/2 study) [27]. In the core phase of the EXIST-1 study, 53% of patients receiving everolimus had a renal angiomyolipoma response [13]. Comparatively, 42% of patients had a renal angiomyolipoma response in the core phase of the similarly designed EXIST-2 study in adults with TSC and renal angiomyolipoma [14]. In a longer term interim analysis from that trial, the response rate had increased to 54% after median treatment duration of 28.9 months [28]. At the conclusion of the current EXIST-1 study, approximately 73% of patients had experienced renal angiomyolipoma response. Our study included fewer and younger patients with renal angiomyolipoma, and it reports longer duration of everolimus exposure than what is currently published for EXIST-2. A final long-term analysis of that study is forthcoming and might provide a better comparison.

Key safety results obtained during this long-term study remained consistent with those previously reported in TSC-associated clinical settings with everolimus [12–14,21,27–29]. No new long-term safety issues arose from this analysis.

Important limitations of this analysis include the open-label design of the extension phase and that the study was not powered or designed to adequately assess other secondary (skin lesions) or exploratory (renal angiomyolipoma) clinical end points. At the end of the study, a majority of patients had not yet completed their sexual development. Ongoing evaluation of sexual development in a subset of these patients is being planned in a follow-up study.

## Conclusions

Long-term efficacy and safety data from the completed EXIST-1 study confirm that everolimus is a safe and effective therapeutic option for patients with TSC-associated SEGA. Moreover, everolimus may reduce the risk for clinical complications of SEGA, such as hydrocephalus and surgery, and use of everolimus effectively manages other TSC manifestations, such as angiomyolipoma and skin lesions. Most AEs appeared in the first year of treatment and were managed by dose adjustment/interruption, and no new safety concerns were raised through this analysis. An upcoming final report on EXIST-2 will provide further assessment of the long-term efficacy and safety of everolimus in treating TSC.

## Supporting Information

S1 Checklist CONSORT 2010 checklist of information to include when reporting a randomized trial. (DOC)

S1 Protocol EXIST-1 study protocol. (PDF)

S1 Table List of ethics committees and/or intuitional review boards that approved the EXIST-1 study. (DOCX)
