# Supplementary material for: Long-Term Use of Everolimus in Patients with Tuberous Sclerosis Complex: Final Results from the EXIST-1 Study
Source: PLoS One. 2016 Jun 28;11(6):e0158476. doi: 10.1371/journal.pone.0158476 (PMC4924870; doi:10.1371/journal.pone.0158476)
Supplement: S1 Protocol — (PDF) [file pone.0158476.s002.pdf]

NOVARTIS

Clinical Development

RAD001 (everolimus)

Clinical Trial Protocol CRAD001M2301

A randomized, double-blind, placebo-controlled study of RAD001 in the treatment of patients with subependymal giant cell astrocytomas (SEGA) associated with Tuberous Sclerosis Complex (TSC)

Document type:	Amended Protocol Clean
EUDRACT number:	2007-006997-27
Version number:	07
Development phase:	III Document
status:	Final
Release date:	15-Jan-2013

Property of Novartis
Confidential
May not be used, divulged, published or otherwise disclosed
without the consent of Novartis

Table of contents

Table of contents 2
List of tables 6
List of figures 7
List of abbreviations 8
Amendment 7 12
Summary of previous amendments 13
Amendment 6 13
Amendment 5 14
Amendment 3 and 4 15
Oncology clinical study protocol synopsis 17
1 Background 33
1.1 Overview of Tuberous Sclerosis Complex (TSC) and Subependymal Giant Cell Astrocytomas (SEGA) 33
1.2 Volumetric assessment of tumor response 34
1.3 Overview of RAD001 35
1.3.1 RAD001 35
1.3.2 The role of mTOR pathway tumorigenesis 36
1.3.3 Preclinical studies 37
1.3.4 Clinical experience 38
1.3.5 Rational for targeted dose 41
1.3.6 Safety in phase I to III oncology studies 41
1.3.7 Phase I study of everolimus in pediatric patients with refractory solid tumor 43
1.3.8 RAD001 phase II study in SEGA 44
1.4 History of Amendments 47
1.4.1 Amendment 1 47
1.4.2 Amendment 2 48
2 Study rationale/purpose 49
3 Objectives 50
3.1 Primary objectives 50
3.2 Secondary objectives 50
4 Study design 51
4.1 Pre-treatment phase (Screening/Baseline) 51
4.2 Blinded treatment phase 56

4.3 Open-label treatment phase ... 58
4.4 Follow-up phase ... 59
4.5 Extension phase ... 60
5 Population ... 62
5.1 Inclusion criteria ... 62
5.2 Inclusion criteria ... 62
5.3 Exclusion criteria ... 64
6 Treatment ... 65
6.1 Investigational and control drugs ... 65
6.1.1 Known undesirable effects of study drug/treatment ... 66
6.1.2 Dosing modifications in case of treatment-related toxicities ... 68
6.1.3 Study drug ... 71
6.2 Treatment arms ... 71
6.3 Patient numbering ... 72
6.4 IWRS procedure ... 72
6.5 Treatment assignment ... 73
6.6 Treatment blinding ... 74
6.7 Treating the patient ... 75
6.7.1 Study drug administration ... 75
6.7.2 Permitted study drug adjustments ... 76
6.7.3 Concomitant medications ... 78
6.7.4 Study drug interruption or discontinuation ... 80
6.7.5 Withdrawal from the study and study evaluation completion ... 81
6.7.6 Emergency unblinding of treatment assignment ... 82
6.7.7 Treatment compliance ... 82
7 Visit schedule and assessments ... 83
7.1 Information to be collected on screening failures ... 101
7.2 Inclusion/exclusion criteria ... 101
7.3 Patient demographics/other baseline assessments ... 101
7.3.1 Baseline assessments of SEGAs and angiomyolipomata ... 101
7.3.2 Special laboratory tests ... 102
7.4 Treatments ... 103
7.5 Efficacy ... 104
7.5.1 Radiological evaluation ... 104
7.5.2 SEGA response evaluation ... 104
7.5.3 SEGA ... 104
7.5.4 Angiomyolipoma response evaluation ... 105

4.3 Open-label treatment phase ... 58
4.4 Follow-up phase ... 59
7.6 Safety ... 108
7.6.1 Adverse events ... 109
7.6.2 Chest CT scan ... 110
7.6.3 Physical examination ... 111
7.6.4 Vital signs ... 112
7.6.5 Body surface area ... 112
7.6.6 Performance status scale ... 113
7.6.7 Laboratory evaluations ... 114
7.6.8 Pulmonary function tests ... 116
7.7 Pharmacokinetics ... 117
7.7.1 Pharmacokinetic (PK) blood sample collection and handling ... 117
7.7.2 RAD001 pharmacokinetic sample shipment ... 123
7.7.3 Analytical method ... 124
7.8 Biomarkers ... 124
8 Safety monitoring ... 125
8.1 Serious adverse event reporting ... 126
8.2 Pregnancies ... 127
8.3 Data Monitoring Committee ... 127
8.4 Steering Committee ... 128
9 Data review and data management ... 128
9.1 Site monitoring ... 128
9.2 Data collection ... 129
9.3 Database management and quality control ... 129
10 Statistical methods and data analysis ... 130
10.1 Populations for analysis ... 130
10.2 Patient demographics/other baseline characteristics ... 131
10.3 Treatments (study drug, concomitant therapies, compliance) ... 131
10.3.1 Study medication ... 131
10.3.2 Concomitant therapies ... 131
10.4 Primary objective ... 131

10.4.1 Variable 131
10.4.2 Statistical hypothesis, model, and method of analysis 131
10.4.3 Handling of missing values/censoring/discontinuations 132
10.4.4 Supportive analyses 132
10.5 Secondary objectives 133
10.5.1 Frequency of epileptiform events 133
10.5.2 Time to SEGA progression 133
10.5.3 Skin lesion response rate 134
10.5.4 Multiplicity adjustment for analysis of main secondary endpoints 134
10.5.5 Additional secondary efficacy analyses 135
10.5.6 Safety 136
10.5.7 Tolerability 138
10.5.8 Resource utilization 138
10.5.9 Pharmacokinetics 138
10.6 Interim analysis 140
10.7 Sample size calculation 140
11 Administrative procedures 142
12 Protocol adherence 144
13 References (available upon request) 145

List of tables

Table 1-1 Active drug substance ... 36
Table 1-2 Steady-state RAD001 pharmacokinetics (daily dosing) ... 40
Table 1-3 Adverse events suspected to be drug-related in greater or equal to 10 percent of patients with advanced cancers reported in phase I RAD001 monotherapy [Studies C2101, C2102, C2107] ... 42
Table 1-4 Primary tumor volume responses ... 45
Table 1-5 Total tumor volume responses ... 45
Table 4-1 Action to be taken for positive baseline hepatitis B results for patients that are active prior to Amendment 2 approval ... 54
Table 4-2 Guidelines for management of hepatitis B for patients that are active prior to Amendment 2 approval ... 54
Table 4-3 Guidelines for management of hepatitis C for patients that are active prior to Amendment 2 approval ... 55
Table 5-1 Diagnostic Criteria for Tuberous Sclerosis Complex ... 64
Table 6-1 Everolimus dose modification guidelines for non-hematologic toxicities ... 68
Table 6-2 Dose modification guidelines for hematologic toxicities ... 69
Table 6-3 Management of non-infectious pneumonitis ... 70
Table 6-4 RAD001 dose levels for dose adjustment ... 77
Table 6-5 Clinical relevant drug interaction: inducers and inhibitors of isoenzyme CYP3A ... 79
Table 6-6 Clinical relevant drug interactions medicated by PgP ... 79
Table 7-1 Blinded phase visit evaluation schedule ... 84
Table 7-2 Open-label and extension phase visit evaluation schedule ... 93
Table 7-3 Physician's Global Assessment of Clinical Condition (PGA) ... 108
Table 7-4 PK Sample log table for visits 1-99 (Refer to Table 7-1) ... 119
Table 7-5 PK sample log table for visits 101-199 (Refer to Table 7-2) ... 121
Table 7-6 PK sample log for full PK profile (Amendment 5) ... 122
Table 10-1 Definition of potential prognostic factors ... 132
Table 10-2 Sensitivity of study power to treatment by stratum interaction, with balanced strata sizes ... 141
Table 10-3 Sensitivity of study power to treatment by stratum interaction, with unbalanced strata sizes ... 141
Table 10-4 Sensitivity of study power to size of treatment effect ... 142

List of figures

Figure 2-1 Survival in a mouse brain model of TSC 50
Figure 4-1 Study Flowchart 61

List of abbreviations

AACR	American Association for Cancer Research
Ab	Antibodies
ADR	Adverse Drug Reaction
AE	Adverse Event
ALT	Alanine Aminotransferase
ANC	Absolute Neutrophil Count
ANCOVA	Analysis of Covariance
ASCO	American Society of Clinical Oncology
AST	Aspartate Aminotransferase
ATC	Anatomical Therapeutic Chemical
ATS	American Thoracic Society
AUC	Area Under the Curve
BAL	Bronchoalveolar Lavage
BASC-2	Behavioral Assessment System for Children, Second Edition
BFGF	Basic Fibroblast Growth Factor
BRIEF	Behavior Rating Inventory of Executive Function
BSA	Body Surface Area
BUN	Blood Urea Nitrogen
C_{2h}	Concentration at 2-hour timepoint
CANTAB	Cambridge Neuropsychological Test Automated Battery
CCR	Complete Clinical Response
C_{max}	Maximum Concentration
CMH	Cochran-Mantel-Haenszel
C_{min}	Minimum (trough) Concentration
CoA	Coenzyme A
CPK	Creatine Phosphokinase
CPO	Country Pharmaceutical Organization
CrCl	Creatinine Clearance
CRF	Case Report Form
CRO	Contract Research Organization
CSF	Cerebral Spinal Fluid
CSF	Cerebral Spinal Fluid
CT	Computed Tomography
CTCAE	Common Toxicity Criteria for Adverse Events
CTH	Clinical Trial Head
CV	Coefficient of Variation
CYP3A	Cytochrome P450 3A4
DL_{CO}	Carbon Monoxide Diffusion Capacity
DLT	Dose-limiting Toxicity
DMC	Data Monitoring Committee
DNA	Deoxyribonucleic Acid
ECG	Electrocardiogram

EDTA	Ethylenediaminetetraacetic Acid
EEG	Electroencephalogram
EIAED	Enzyme- Inducing Antiepileptic Drugs
ELISA	Enzyme-linked Immunosorbent Assay
EOT	End of Treatment
ERS	European Respiratory Society
FAS	Full Analysis Set
FDA	US Food and Drug Administration
FEV_1	Forced Expiratory Volume in One Second
FGF	Fibroblast Growth Factor
FOV	Field of View
FPFV	First Patient First Visit
FSE	Fast Spin Echo
FSH	Follicle- stimulating Hormone
GCP	Good Clinical Practice
GFR	Glomerular Filtration rate
GI	Gastrointestinal
GIST	Gastrointestinal Stromal Tumor
HBcAb	Hepatitis B core antibodies
HBs Ab	Hepatitis B surface antibodies
HBsAg	Hepatitis B surface antigen
HBV	Hepatitis B virus
HCT	Hematocrit
HCV	Hepatitis C virus
HDL	High Density Lipoprotein
HIV	Human Immunodeficiency Virus
HMG	3-hydroxy-3-methyl-glutaryl
HUVEC	Human Umbilical Vein Endothelial Cell
IB	Investigator’s Brochure
IC_{50}	Half Maximal Inhibitory Concentration
ICH	International Conference on Harmonization
IEC	Independent Ethics Committee
IHC	Immunohistochemistry
IMS	Integrated Medical Safety
IN	Investigator Notification
IND	Investigational New Drug Application
INR	International Normalized Ratio
IRB	Institutional Review Board
IWRS	Interactive Web Response System
LC-MS	Liquid Chromatography-mass Spectrometry
LDH	Lactate Dehydrogenase
LDL	Low Density Lipoprotein
LFTs	Liver function tests
LH	Luteinizing Hormone

LLOQ	Lower Limit Level of Quantification
LPLV	Last Patient Last Visit
MedDRA	Medical Dictionary for Regulatory Activities
mg/d	Milligrams per day
MPD	Molecular Pharmacodynamic
MRI	Magnetic Resonance Imaging
MRP	Mutual Recognition Procedure
MSD	Meso Scale Discovery
MTD	Maximum Tolerated Dose
mTOR	Mammalian Target of Rapamycin
NCI	National Cancer Institute
NET	Neuroendocrine tumors
O_2	Oxygen
p.o.	per os/by mouth/orally
PCR	Polymerase Chain Reaction
PD	Progressive Disease
PFT	Pulmonary Function Test
PGA	Physician’s Global Assessment of Clinical Condition
PgP	P-glycoprotein
pH	Negative Log Hydrogen Ion Concentration
PI3K	Phosphatidylinositol 3-kinase
PK	Pharmacokinetic
PLGF	Placental Growth Factor
PPS	Per Protocol Set
PR	Partial Response
PT	Prothrombin Time
PTS	Post-text Supplements
PTT	Partial Thromboplastin
RBC	Red Blood Cells
REB	Research Ethics Board
RECIST	Response Evaluation Criteria in Solid Tumors
RFS	Recurrence- free Survival
RNA	Ribonucleic acid
ROI	Region of Interest
s.c.	Subcutaneous
SAE	Serious Adverse Event
SCL-90-R	Symptom Checklist -90-Revised
SD	Stable Disease
SEGA	Subependymal Giant Cell Astrocytoma
SEN	Subependymal Nodule
SGOT	Serum Glutamic Oxaloacetic Transaminase
SGPT	Serum Glutamic Pyruvic Transaminase
SI	Système International
SSC	Study Steering Committee

SSQ	Seizure Severity Questionnaire
$t_{1/2}$	Half-life
t_{max}	Maximum time
TS	Tuberous Sclerosis
TSC	Tuberous Sclerosis Complex
TTSP	Time to SEGA Progression
ULN	Upper Limit of Normal
URS	User Requirement Specification
URTI	Upper Respiratory Tract Infection
VABS	Vineland Adaptive Behavior Scale
VEGF	Vascular Endothelial Growth Factor
Vz/F	Distribution Volume
WASI	Wechsler Abbreviated Scale of Intelligence
WBC	White Blood Cell
WHO	World Health Organization
WPPSI	Wechsler Preschool and Primary Scale of Intelligence
WRAML-2	Wide Range Assessment of Memory and Learning

Amendment 7

Amendment rationale

At the time of this protocol amendment, enrollment is complete. There are approximately 100 active patients being followed in the extension phase of the study.

This protocol is being amended in order to correct a single paragraph at the end of Amendment 6 which stated that the amendment (i.e. Amendment 6) was required for patient safety due to an immediate hazard.

Text in the aforementioned paragraph of Amendment 6 inadvertently implied that there is an Urgent Safety Measure (USM) which requires immediate protocol amendment implementation. Please note that there was no USM requiring immediate implementation and the Substantial Amendment (i.e. Amendment 6) *should not* be implemented prior to IRB/IEC approvals.

Changes to the protocol

1. In the section, Amendment 6, Changes to the protocol, the following paragraph is deleted: "This amendment is required for patient safety (i.e. necessary to eliminate immediate hazards to the trial subjects ICH GCP 3.3.8). Therefore it will be implemented prior to IRB/IEC approval, but will be sent for approval as well."
2. In the section, Amendment 6, Changes to the protocol, the following paragraph is added: "The changes described in this amended protocol require IRB/EC approval prior to implementation. In addition, if the changes herein affect the Informed Consent, sites are required to update and submit for approval a revised Informed Consent that takes into account the changes described in this amended protocol."

Changes to specific sections of the protocol are shown in the track changes version of the protocol using ~~strike through red font for deletions~~ and <u>red underlined for insertions.</u>

A copy of this amended protocol will be sent to the Institutional Review Board (IRBs)/Independent Ethics Committee (IECs) and Health Authorities.

The changes described in this amended protocol require IRB/EC approval prior to implementation. In addition, if the changes herein affect the Informed Consent, sites are required to update and submit for approval a revised Informed Consent that takes into account the changes described in this amended protocol

Summary of previous amendments

Amendment 6

Amendment rationale

At the time of this protocol amendment, enrollment is complete. There are approximately 100 active patients being followed in the extension phase of the study. Thirteen active females have had their first menses. Twenty eight females enrolled who are still active and will turn 10 years old while on study.

This protocol is being amended in order to apply the Novartis guideline on prevention of pregnancy in clinical trials and gather more patient information on menstrual history and status.

The potential reproductive risk of RAD001 for humans is unknown. Changes in the methods of birth control have been updated from adequate to highly effective contraception methods in line with Novartis' guidance for prevention of pregnancies in clinical trials. The urine pregnancy testing for female patients of child bearing potential has changed from every 12 weeks to every 4 weeks.

In addition, female patients of child-bearing potential will provide further details of pregnancy and menstrual history as well as provide monthly updates to the investigator on menstrual status.

Changes to the protocol

1. Sections 5.1: Changed inclusion criteria to include highly effective contraceptive measures instead of adequate contraceptive measures.
2. Section 6.1.1: Added secondary amenorrhea as an identified risk of study drug. Added information on management of secondary amenorrhea.
3. Sections 7.3.2.1, 7.6.7.6: Added detailed description of highly effective contraceptive measures.
4. Section 7.3.2.4: Added completion of hormone evaluations when amenorrhea is seen between scheduled assessments.
5. Section 7: Revised Table 7-1 and 7-2. Urine pregnancy testing frequency increased from every 12 weeks to every 4 weeks.
6. Section 7: Revised Table 7-1 and 7-2. Serum pregnancy test added to End of Treatment evaluation.
7. Section 7: Revised Table 7-1 and 7-2. Additional menstrual history and pregnancy history information collected. Will conduct monthly monitoring of menstrual status.
8. Section 7: Revised Table 7-1 and 7-2. Added collection of biological parental height to assist us in the evaluation of patient's growth and development.
9. Section 7.3: Urine pregnancy testing frequency increased from every 12 weeks to every 4 weeks. Serum pregnancy test added to End of Treatment evaluation.

10. Section 7.3: Parental height subsection added. Data will be used to evaluation patient's growth and development.
11. Section 7.6.7.7: Revised endocrine testing section to include reproductive history. Additional medical information on reproductive history will be collected.
12. Section 8.2: Additional safety language for pregnancies added
13. Section 9.2: Added data collection language regarding the results from at home urine pregnancy to be recorded on patient diaries for source documentation only.

Changes to specific sections of the protocol are shown in the track changes version of the protocol using ~~strike through red font for deletions~~ and red underlined for insertions.

A copy of this amended protocol will be sent to the Institutional Review Board (IRBs)/Independent Ethics Committee (IECs) and Health Authorities.

The changes described in this amended protocol require IRB/EC approval prior to implementation. In addition, if the changes herein affect the Informed Consent, sites are required to update and submit for approval a revised Informed Consent that takes into account the changes described in this amended protocol.

Amendment 5

Amendment Rationale

At the time of this protocol amendment enrollment is complete. There are 106 active patients being followed in the extension phase of the study.

This protocol is being amended due to a post marketing health authority request to obtain more detailed pharmacokinetic information among the pediatric population. It is not being amended because of any safety concerns. The data generated will be combined with the C_{min} and C_{2hr} data already collected during the study to better determine potential pharmacokinetic differences among pediatric patients.

As part of this amendment, a full pharmacokinetic (PK) profile will be collected from patients currently active in the study. This assessment will include the impact of age, weight, BSA and co-administration of CYP3A4/PgP enzyme inducers to complement the current understanding of the disposition of everolimus in this patient group.

Additional PK samples will be collected in all patients who consent to take part in this amendment, during any ONE of their regularly scheduled study visits. Recruitment for this PK evaluation is voluntary and will be based on the consent of currently active patients to participate.

The additional PK collection time points will be: predose and at 0.5 hours post dose, 1 hour post dose, 2 hours post dose, 5 hours post dose and 24 hours post dose.

Changes to Protocol

- Synopsis, Section 4.5, Table 7-1 and Section7-2 and Section 7.7.1
  - Added information regarding new PK collection timepoints and meal intake guidelines for patients participating in full PK profile

- Section 6.4
    - Added information on how the IWRS system will be used to track the patients who participate in the full PK profile collection
- Table 7-6
    - Added table to reflect indicate the collection and sample numbers for the full PK profile collections
- Synopsis and Section 10.5.9
    - Added information to define how the full PK profile data will be analyzed

Amendment 3 and 4

Amendment rationale

Some cases of low testosterone level (age-adjusted) and luteinizing hormone (LH) >15 IU/L were reported in the transplant setting. Under the previous amendment, endocrine testing was completed every 12 weeks for those patients that did not exhibit secondary sexual characteristics by age 13 (females) or age 14 (males). This amendment allows the evaluation of any potential effects of everolimus on hormone levels of all patients enrolled into the trial. Under this amendment, all patients will have endocrine testing (Testosterone, FSH, LH and Estradiol (for females) at baseline or at their next scheduled visit (if no prior endocrine testing had been done). In addition, follow-up endocrine blood sampling will be completed annually until the patient's 10th birthday and then every 12 weeks thereafter. For patients who are 10 years old or older at the time of this amendment, endocrine blood sampling will be completed every 12 weeks.

At the time of this protocol amendment, enrollment is complete with 117 patients having been randomized. One hundred and thirteen patients are still receiving trial therapy and four patients have discontinued trial therapy.

Changes to the protocol

Amendment 4

- Synopsis, Section 4.2, Table 7-1, Table 7-2, Section 7.3.2.4, Section 7.6.7.7
    - Added endocrine blood testing (testosterone, LH, FSH and Estradiol) at screening and subsequent timepoints according to the patient's age

Amendment 3

- Synopsis
    - New time points at which Tanner Staging and endocrine testing will be assessed
    - Addition of the collection of retrospective height and weight data if it is available
    - Changed the Hepatitis C reactivation language to HCV flare
- Section 1.3.1, RAD001

    - New everolimus United States approval information regarding Zortress for the prophylaxis of organ rejection in adult patients at low-moderate immunologic risk receiving a kidney transplant and Afinitor for the treatment of SEGA associated with Tuberous Sclerosis who require therapeutic intervention but are not candidates for curative surgical resection.
- Section 1.4, History of Amendments
    - The history of amendments section has been moved to the front of the document.
- Sections 4.1, Pre-treatment phase (Screening/Baseline) and 4.2, Blinded treatment phase
    - Time points at which Tanner Staging and Endocrine testing will be assessed have been updated
    - Addition of the collection of retrospective height and weight data if it is available
    - Updated the guidelines for the management of Hepatitis B and C
- Section 4.3, Open-label treatment phase
    - Updated to only require a repeat of baseline open-label imaging and EEG procedures if they occurred more than 12 weeks prior to the start of open-label treatment.
- Table 6-3, Management of non-infectious pneumonitis
    - Updated the table to correct a typographical error from the previous version. For Grade 2 and 3 non-infectious pneumonitis, treatment should be interrupted until return to Grade 1 or lower.
- Table 7-1 and Table 7-2, Schedule of Assessments
    - Both tables were updated to reflect the new assessment time points for Tanner Staging and endocrine testing.
    - Additionally, Exam*One* was removed as a local laboratory option for patients in the United States.
    - Added that the physical exam must include documentation of sexual maturation milestone achievement (adrenarche, menarche and thelarche).
- Section 7.3, Patient demographics/other baseline assessments
    - Updated to include the collection of all historical height and weight data that is available for each patient
- Section 7.3.3.3, Endocrine testing
    - Updated the endocrine testing section with the new time points for sample collection
- Section 7.6.3.1 Tanner Staging
    - Added guidelines for how Tanner Staging should be assessed for both genders
- Section 10.5.6.3, Growth data
    - Added section to outline how the growth and sexual maturity data will be analyzed.

See Section 1.4 for a summary of previous amendments

Oncology clinical study protocol synopsis

**Protocol no.**	CRAD001M2301
**Study phase**	III
**Study title**	A randomized, double-blind, placebo-controlled study of RAD001 in the treatment of patients with subependymal giant cell astrocytomas (SEGA) associated with Tuberous Sclerosis Complex (TSC).
**Background**	TSC is an autosomal dominant genetic disorder caused by inactivating mutations in the TSC1 or TSC2 genes, and is characterized by benign, highly vascular, hamartoma growth. Lesions occur in the brain, kidneys, heart, liver, lungs and skin, leading to renal complications, pulmonary failure, autism, mental retardation, seizures and epilepsy (Gomez et al 1999; Astrinidis and Henske 2005; Inoki et al 2005; Kwiatkowski and Manning 2005). Measures of childhood prevalence range from 1 in 6,800 to 1 in 17,300 but full ascertainment is difficult to achieve (Yates 2006). Brain lesions are the primary cause of morbidity and mortality in this disorder in childhood.<br>The incidence of subependymal giant cell astrocytoma (SEGA) in TSC varies from 5 to 15%. SEGAs are slow growing lesions that are typically unapparent clinically until they reach sufficient size to produce ventricular obstruction and hydrocephalus. By the time symptoms are noted they are often irreversible even by emergent surgical intervention. They arise deep within the brain in the region of the foramen of Monro, which hampers their surgical resection, as the approach to the lesion entails removal of substantial amounts of viable cerebral tissue. Surgery, even when successful, often results in significant morbidity.<br>The TSC1/TSC2 protein complex is a negative regulator of the mammalian target of rapamycin (mTOR) pathway. Hence, mutation or loss of either of these gene products in preclinical models is associated with increased mTOR pathway activation and heightened sensitivity to mTOR inhibitors (Astrinidis and Henske 2005; Inoki et al 2005; Kwiatkowski and Manning 2005).<br>mTOR pathway upregulation has also been observed in lesions derived from TSC patients (Astrinidis and Henske 2005; Kwiatkowski and Manning 2005), and TSC1 or TSC2 defective experimental animal models exist which recapitulate the pathology, behavioral and neurological aspects of the tuberous sclerosis disease (Onda et al 1999; Kwiatkowski and Manning 2005; Uhlmann et al 2002; Kenerson et al 2005) and are sensitive to mTOR inhibition (Kenerson et al 2005; Astrinidis and Henske 2005; Kwiatkowski and Manning 2005). In this respect, experiments are in progress aimed at analyzing the effects of RAD001 in animal models of TSC (D. Kwiatkowski/Novartis collaboration). Preliminary data indicate that gross kidney lesion scores can be significantly reduced by RAD001 treatment in both TSC1+/- and TSC2+/- mouse models ($p = 0.02$ compared to untreated controls, unpaired t-test). mTOR inhibition is associated with a dramatic inhibition of the phosphorylation of the ribosomal S6 protein in treated kidney lesions (S6 phosphorylation is an established pharmacodynamic marker of mTOR pathway activation status). Furthermore, a dramatic improvement in survival has been observed in a mouse brain model of TSC (genotype : Tsc1cc syn-cre+), with a highly statistically significant improvement in survival ($p < 0.0001$) associated with RAD001 treatment (Kenerson 2005). The investigator also noted an improvement in behavior, weight gain and neurological phenotype. Assessment of brain pathology is currently ongoing. Finally, both estrogen and vascular endothelial growth factor (VEGF) signaling have been implicated in the pathogenesis and vascularization of TSC lesions (Astrinidis and Henske 2005; Kwiatkowski and Manning 2005). In this respect, RAD001 has been shown to inhibit both estrogen and VEGF-dependent signaling events (Boulay et al 2005; O'Reilly et al 2005). Taking all these data into

	account, there is a strong rationale for using RAD001 for the treatment of patients with tuberous sclerosis.
**Purpose/ rationale**	This study will evaluate the antitumor activity of RAD001 versus placebo in patients with SEGA associated with TSC.
**Objectives**	**Primary objective**:<br>To compare the SEGA response rate on RAD001 versus placebo in patients with TSC-associated SEGA.<br>**Secondary objectives**:<br>**To compare RAD001 versus placebo with respect to**:<br>1. Change from baseline in frequency of epileptiform events.<br>2. Time to SEGA progression.<br>3. Skin lesion response rate.<br>4. Change from baseline in plasma angiogenic molecules, e.g. VEGF, basic FGF, PLGF, soluble VEGF receptor1, and soluble VEGF receptor2.<br>5. Renal function assessed using calculated creatinine clearance.<br>6. Safety as assessed by the NCI Common Toxicity Criteria, version 3.0.<br>**In RAD001 treatment arm to** :<br>7. Characterize the pharmacokinetics of RAD001 in this patient population, specifically in terms of exposure.<br>8. Describe the time to SEGA response, duration of SEGA response, and the duration of skin lesion response.

Endpoints (efficacy, safety)	**Efficacy endpoints**<br>**Primary endpoints**: SEGA response rate is determined from the Independent Central Radiological Review of MRIs. SEGA response rate is defined as the proportion of patients with a reduction in SEGA volume of at least 50% relative to baseline, where SEGA volume is the sum of the volumes of all target SEGA lesions identified at baseline, and confirmed with a second scan approximately 12 weeks later (and no sooner than 8 weeks later).<br>In addition, response requires that:<br>• the non-target SEGA lesions have not unequivocally worsened<br>• no new SEGA lesions (of ≥1cm in longest diameter) are identified and<br>• the absence of new or worsening hydrocephalus (defined by central radiological assessment of ventricular configuration changes, ventricular cap signs [periventricular edema) and qualitative assessment of cerebrospinal fluid [CSF] flow dynamics).<br>SEGA response rate will be determined in the Full Analysis Set (FAS), defined as all randomized patients analyzed according to the treatment they were assigned to at randomization.<br>**Secondary endpoints**:<br>1. Absolute change from baseline in frequency of epileptiform events per 24 hours, using data obtained from the 24-hour video EEG at baseline and week 24.<br>2. Time to SEGA progression, determined from the Independent Central Radiological Review of MRIs. SEGA progression is defined as one or more of the following:<br>• an increase from nadir of 25% or more in SEGA volume to a value greater than baseline SEGA volume (where SEGA volume is the sum of the volumes of all target SEGA lesions identified at baseline and where nadir is the lowest SEGA volume achieved by the patient previously in the trial [including baseline]), or<br>• the unequivocal worsening of non-target SEGA lesions, or<br>• the appearance of a new SEGA lesion ≥ 1 cm in longest diameter, or<br>• new or worsening hydrocephalus defined by central radiological assessment of ventricular configuration changes, ventricular cap signs (periventricular edema) and qualitative assessment of CSF flow dynamics.<br>3. Skin lesion response rate using the Physician’s Global Assessment of Clinical Condition (PGA), where response is defined as complete clinical response or partial response, and where the denominator only includes patients with at least one skin lesion at baseline.<br>4. Change from baseline in angiogenesis markers.<br>**In RAD001 treatment arm**:<br>1. Exposure of RAD001 in treated patients.<br>2. Duration of SEGA response and time to SEGA response, both determined from the Independent Central Radiological Review of MRIs. Duration of SEGA response is defined as the time from the first confirmed SEGA response until SEGA progression; time to SEGA response is the time from randomization until the first SEGA response. Duration of response of skin lesions, defined as the time from the first skin lesion response until skin lesion progression, where response and progression are based on the Physician’s Global Assessment of Clinical Condition (PGA).<br>**Safety endpoints**

	Safety will be assessed by the National Cancer Institute's (NCI) Common Toxicity Criteria for Adverse Events (CTCAE), version 3.0 (http://ctep.cancer.gov/protocolDevelopment/electronic_applications/docs/ctcaev3.pdf).<br>**Notes**:<br>• Safety assessments will consist of monitoring and recording all adverse events, including serious adverse events, and the regular monitoring of vital signs, physical condition, hematology and blood chemistry, and body weight. Renal function will be assessed using an age appropriate measure; either the Cockcroft and Gault formula (Cockcroft and Gault 1976) for patients 18 years and older or the Schwartz formula for patients up to age 18 (Schwartz et al 2009).<br>• Pneumonitis is a known side-effect of mTOR inhibitors. Investigators should be vigilant for signs and symptoms of pneumonitis, such as cough, dyspnea and malaise. If the condition is suspected, investigations such as pulmonary function tests, chest CT and referral to a pulmonologist should be considered. For more detailed management advice see Table 6-3.<br>• Hematology and chemistry assessment will be done at screening and at each scheduled visit thereafter throughout the study.
**Study Design**	This is a prospective, double-blind, randomized, parallel group, placebo-controlled, multi-center phase III study evaluating treatment with RAD001 versus placebo in 99 patients with TSC-associated SEGA. The study will use an Interactive Web Response System (IWRS) for patient randomization and for medication management. The randomization ratio is 2:1 in favor of RAD001. Randomization will be stratified by the use of enzyme-inducing anti-epileptic drugs (EIAED: yes versus no). The following drugs qualify as EIAED: phenytoin (Dylantin®, Dilantin Kapseals®, Dilantin Infatabs®, Eptoin®, Epanutin®, Diphenin®, Dipheninum®, Phenytek®), mephenytoin (Mesantoin®), carbamazepine (Tegretol®, Biston®, Calepsin®, Carbatrol®, Epitol®, Equetro®, Finlepsin®, Sirtal®, Stazepine®, Telesmin®, Teril®, Timonil®, Trimonil®, Epimaz®, and Degranol®), phenobarbital (Luminal®), pentobarbital (Nembutal®), primidone (Mysoline®), and oxcarbazepine (Trileptal®).<br>**Screening/Baseline phase**: Screening/baseline evaluations will be performed within 28 days prior to treatment day 1. An MRI of the brain should be performed for the baseline tumor assessment and all SEGA lesions should be identified as either target (longest diameter of at least 1 cm) or non-target (longest diameter less than 1 cm). CT or MRI of the kidneys will also be performed for all patients to identify any angiomyolipomata. NOTE: If local or country requirements prohibit the use of CT, then only an MRI of the abdomen may be completed. Other TSC-associated lesions, e.g., tubers, SENS, skin lesions, should also be identified. A pre-baseline MRI of the brain, conducted prior to the baseline MRI, must be obtained and used by the investigator and the local radiologist to determine whether the patient satisfies the radiological criteria for entry into the trial (for additional details, see point 4 of the inclusion criteria, Section 5.1). Each participating center should assess these radiological criteria according to their own clinical practice using the best methods available to them. It is known that not all sites will have the technical capability to precisely measure SEGA volume and ventricular volume, and also that some pre-baseline MRIs may not be in digital format. In such cases qualitative assessments should be made.<br>If upon local review of the baseline brain scan, the longest diameter of the largest SEGA lesion is less than 1.5 cm (between 1.0 cm and 1.4 cm), the baseline scan must immediately be sent to the central reviewer prior to the patient being randomized. The central reviewer will confirm whether the patient's SEGA meets the minimum longest diameter criterion of 1.0 cm and provide feedback to the

	investigator within 1 week of receiving the scan. The investigator can then proceed with randomization provided all eligibility criteria have been confirmed and the central reader agrees that the patient has a SEGA lesion of at least 1.0 cm in longest diameter. If, according to central review, the patient does not have a SEGA lesion of at least 1.0 cm, the patient will be considered a screen failure and should not be randomized. The serial growth (inclusion criterion # 4) criteria will be reviewed and confirmed locally. Therefore, the prebaseline scan will not need to be sent to the central reader until after randomization.<br>For patients with longest SEGA diameter greater than 1.4 cm the decision to randomize the patient will be made based on the judgment of the investigator and local radiologist.<br>As with the local radiological review, if the pre-baseline MRI was only available on film (non-digital format), then the central reviewer should make a qualitative assessment of any increase in SEGA volume, appearance of a new SEGA lesion or new or worsening hydrocephalus.<br>There is no plan to collect radiological measurements made by the local radiologist, partly because not all sites will have the same software for measuring lesion volume, but also because all data analysis will be based on the measurements obtained from the central radiological review.<br>Once the patient is confirmed as being eligible to be randomized, a 24-hour video EEG will be conducted and sent for independent central review. Once the patient is randomized, the baseline brain MRI (if the SEGA lesion is greater than 1.4 cm in its longest diameter) and kidney CT/MRI should be sent as soon as possible by the site to the Independent Central Radiology Reviewer.<br>All patients **being treated with antiepileptics at baseline** will complete a seizure severity questionnaire at baseline, whenever possible. All of the above assessments/procedures must be conducted prior to randomization.<br>**Blinded treatment phase/Duration of treatment**: Patients will be randomized to receive either RAD001 or matching placebo. Patients will be treated with blinded study treatment until SEGA progression (as defined in Section 7.5.2), unacceptable toxicity or discontinuation for any other reason. The starting dose is 4.5 mg/m^2/day. Dose adjustments will be permitted based on safety findings and blood trough measurements. A detailed explanation of permitted dose adjustments and the process that will be implemented to maintain the blind can be found in Section 6.7.2, "Permitted Study Drug Adjustments."<br>During the blinded treatment phase, brain tumor assessments using MRI will be performed at 12, 24 and 48 weeks after start of study treatment, and annually thereafter, until SEGA progression. For patients with target angiomyolipomata (longest diameter ≥ 1.0 cm) identified at screening/baseline, kidney assessments using CT/MRI will also be performed at 12, 24 and 48 weeks after start of study treatment, and annually thereafter. For each patient, the same imaging modality must be used throughout the trial. An additional MRI should be performed to confirm SEGA response at approximately 12 weeks (and no sooner than 8 weeks) after it was first observed. For patients who respond at 12 weeks of treatment, the routine 24 week scan is sufficient to confirm response. All imaging of the brain and kidneys conducted during the blinded phase of the trial should be sent in for central radiological review within 2 days of the scan. For all patients, a 24-hour video EEG and, whenever possible, a seizure severity questionnaire (for those patients being treated with antiepileptics at baseline), will be completed at 24 weeks, or at end of treatment if the patient discontinues before 24 weeks. The 24-hour video EEG will be sent for independent central review.<br>**Open-label treatment phase**: If SEGA progression (as defined in Section 7.5.2) is documented by central radiology review during the blinded treatment phase, then

	the treating physician may proceed to unblind the patient. Central radiology review of a MRI will be completed no later than 3 weeks after its receipt by the central reviewer, and so the unblinding may not be able to take place until up to 3 weeks after the progression was actually observed. However, if progression was **unequivocal** according to the local radiologist, then the investigator should contact the Novartis Clinical Trial Head (CTH) or designee to proceed with unblinding without waiting for confirmation from central radiology review.<br><br>Following unblinding, patients who had been receiving placebo may be offered open-label treatment with RAD001 if the treating physician believes the patient could benefit from this therapy.<br><br>For the open-label phase of the study, the most recent MRI of the brain from the blinded phase of the study will be used as the baseline, and further MRIs of the brain will be conducted at 12, 24 and 48 weeks after start of open label RAD001 and annually thereafter, until SEGA progression, unacceptable toxicity or discontinuation for any other reason. If CT/MRIs of the kidneys had been conducted in the blinded phase of the study, then CT/MRIs of the kidneys will be performed in the open-label phase at the same assessment times as the MRIs of the brain, and the latest CT/MRI of the kidneys that was done in the blinded treatment phase will be used as the baseline. For each patient, the same imaging modality must continue in the open label phase of the trial as in the blinded phase. All MRIs of the brain and CT/MRIs of the kidneys obtained in the open-label phase of the trial will be sent in for central radiological review within 2 days of the scan. Patients receiving open-label treatment with RAD001 will continue having safety and efficacy assessments as in the blinded phase of the trial, with the exception of biomarker assessments which will not be done.<br><br>Open-label treatment with RAD001 will begin at 4.5 mg/m^2 with up-titration or down-titration possible to achieve the intended RAD001 concentration of 5-15 ng/ml. During the open-label phase, treatment will continue until the patient again presents with SEGA progression (second occurrence) which is either radiologically documented or a SEGA-related surgical intervention. At this point, patients will be discontinued from the study and will enter the follow-up phase.<br><br>**Follow-up (for patients who discontinue study treatment)**: Patients who have not had SEGA progression at the time of study treatment discontinuation will be followed up with MRIs of the brain (and CT/MRIs of the kidney if angiomyolipomata with longest diameter ≥ 1.0 cm were present at baseline) annually until eventual SEGA progression, or until the start of any non-study systemic anti-SEGA therapy, whichever occurs sooner. For each patient, the same imaging modality should be used throughout the trial. During this follow-up period, the site will continue to send imaging scans for central review, and use of non-study systemic anti-SEGA therapies (does not include anti-epileptic medications) will be recorded. In addition, patients will be followed for safety until at least 28 days after study treatment discontinuation and will come in for a final follow-up visit 28 days after study treatment discontinuation. Beyond these 28 days, any serious adverse events that are suspected to be related to the study drug and occur within the next 8 weeks (56 days) will also be collected. Any medications/therapies taken by the patient during the 12 weeks after study treatment discontinuation should be recorded on the CRF during this 84-day period.<br><br>**Extension phase**: The data cutoff date for the final analysis will be 6 months after the last patient is randomized. Once the final trial results are known, and if these results favor RAD001, then an extension phase will be launched. All patients still receiving study treatment at this time, as well as those being followed for post-treatment evaluation, will be given the option of starting open-label RAD001, which will be provided free of charge by Novartis. Those patients entering the extension phase who had previously only been receiving placebo, will have scheduled

	assessments beginning at Baseline according to Table 7-2 and will begin their treatment at 4.5 mg/m^2 with up-titration or down-titration possible to achieve the intended RAD001 blood concentration of 5-15 ng/ml. Accordingly, patients that had been on active RAD001 prior to beginning the extension phase will simply continue their sequence of assessments according to Table 7-1 or Table 7-2 and continue the same dose they were receiving in the blinded phase. The extension phase will run until 4 years after the last patient was randomized, ensuring all patients will be followed up between 4 and 5 years (assuming patient accrual over a 12-month period). Patients will be treated with open-label RAD001 until the end of the extension phase, or until unacceptable toxicity or any other reason for discontinuation. For all patients receiving RAD001 at the end of the extension phase, RAD001 will continue to be provided free of charge for as long as the medication is not commercialized and added to the list of reimbursed medications for patients with TSC.
**Population**	Male or female patients of any age who are diagnosed with TSC-associated SEGAs and have radiological evidence of either (1) serial growth, or (2) a new SEGA lesion ≥ 1.0 cm in its longest diameter, or (3) new or worsening hydrocephalus.
**Inclusion/ Exclusion criteria**	**Inclusion criteria**:<br>1. Male or female of any age.<br>2. Clinically definite diagnosis of tuberous sclerosis according to the modified Gomez criteria (Roach et al 1998; Hyman and Whittemore 2000, Table 5-1). Clinically definite diagnosis is defined as either of the following:<br>• Two Major Features from Table 5-1.<br>• One Major Feature plus two Minor Features from Table 5-1.<br>3. Presence of at least one SEGA lesion ≥ 1.0 cm in its longest diameter using MRI.<br>Note: SEGA lesions are only diagnosed in patients with TSC. They arise in the subependymal layer of the lateral ventricle and are usually located near the foramen of Monro and enhance homogeneously with contrast on MRI with no evidence of surrounding edema.<br>4. A recent MRI of the brain completed within 4 weeks (28 days) prior to the patient's randomization must be compared with an MRI of the brain performed at an earlier stage of patient care (pre-baseline) and should demonstrate at least one of the following:<br>a. Serial growth, defined as at least a 25% increase in SEGA volume, or<br>b. Presence of a new SEGA lesion ≥ 1 cm in its longest diameter, or<br>c. New or worsening hydrocephalus defined by assessment of ventricular configuration changes, ventricular cap signs (periventricular edema) and qualitative assessment of CSF flow dynamics.<br>**Notes:**<br>Patients who have had previous SEGA surgery are eligible provided criterion 4 has been satisfied by comparing the baseline scan to any prebaseline scan that has been conducted following the most recent SEGA surgery.<br>If a previous MRI is not available, a comparative review of two previous CT scans is also acceptable to establish any of the above mentioned three conditions. In this case, baseline/screening MRI should still be performed.<br>If only one prior CT scan was available, a second CT scan should be obtained to allow like comparison of cranial images. Again, the baseline MRI should still be performed. CT scans must be digitized and sent to the central reviewer for confirmation of eligibility.<br>If the pre-baseline MRI or CT scan was only available on film (non-digital format) or

	software for volumetric assessment was not available at the local site, the local radiologist should make a qualitative assessment of the above-mentioned three criteria. The non-digital MRI or CT scan must also be sent to Central Radiology for review.<br>5. If female and of child-bearing potential, documentation of negative pregnancy test prior to enrollment. Sexually active pre-menopausal female patients (and female partners of male patients) must use highly effective contraceptive measures while on study and for up to 8 weeks after ending treatment.<br>6. Written informed consent according to local guidelines.<br>**Exclusion criteria** :<br>1. Patients for whom SEGA-related surgery is likely to be required, in the opinion of the investigator. History of myocardial infarction, angina or stroke related to atherosclerosis.<br>2. Known impaired lung function (e.g. FEV_1 or DL_{CO} ≤ 70% of predicted).<br>3. Significant hematological or hepatic abnormality (i.e., transaminase levels > 2.5 x ULN or serum bilirubin > 1.5 x ULN, hemoglobin < 9 g/dL, platelets < 80,000/ mm^3, absolute neutrophil count < 1,000/mm^3).<br>5. Pregnancy or breast feeding.<br>6. Intercurrent infection at date of randomization.<br>7. Prior history of organ transplantation.<br>8. Recent surgery (involving entry into a body cavity or requiring sutures) within the 2 months prior to randomization.<br>9. Prior therapy with mTOR inhibitors (e.g., sirolimus, temsirolimus, everolimus).<br>10. Use of an investigational drug within the 30 days prior to randomization.<br>11. Uncontrolled hyperlipidemia: Fasting serum cholesterol > 300 mg/dL OR > 7.75 mmol/L AND Fasting triglycerides > 2.5 x ULN.<br>12. Uncontrolled diabetes mellitus as defined by fasting serum glucose > 1.5 x ULN.<br>13. Patients with bleeding diathesis or on oral anti-vitamin K medication (except low dose warfarin).<br>14. Patients with known history of HIV seropositivity.<br>15. Inability to attend scheduled clinic visits.<br>16. For the purpose of MRI assessments:<br>a. Ferromagnetic metal implants other than those approved as safe for use in MR scanner (e.g., braces, some types of aneurysm clips, shrapnel).<br>b. Patients suffering from uncontrollable claustrophobia or physically unable to fit into the machine (e.g., obesity, etc).<br>Note: patients with vagal nerve stimulators are permitted to have CT assessments of angiomyolipomas unless local or national regulations do not permit this.<br>17. Serum creatinine > 1.5 x ULN.<br>18. History of malignancy in the past two years, other than squamous or basal cell skin cancer.<br>19.Any severe and/or uncontrolled medical conditions which could cause unacceptable safety risks or compromise compliance with the protocol, such as:<br>a. ≥ Grade 3 hypercholesterolemia/hypertriglyceridemia or ≥ Grade 2 hypercholesterolemia/hypertriglyceridemia with history of coronary artery disease (despite lipid-lowering treatment if given)

	b. Impairment of gastrointestinal function or gastrointestinal disease that may significantly alter the absorption of study drug (e.g., ulcerative disease, uncontrolled nausea, vomiting, diarrhea, malabsorption syndrome).<br>c. Active skin, mucosa, ocular or GI disorders of Grade > 1
**Investigational and control drugs**	RAD001 or matching placebo
**Dose, regimen, treatment cycle**	In both arms, the starting dose will be 4.5 mg/m2 daily, rounded to the nearest mg.
**Supply, preparation, and administration**	RAD001 is formulated as tablets of 1.0 mg strength dosed on a daily basis. Placebo will be formulated to be indistinguishable from the RAD001 tablets. The blisters for RAD001 / placebo tablets should be opened only at the time of administration as the active drug is both hygroscopic and light-sensitive. Both RAD001 and matching placebo will be provided and supplied centrally by Novartis.<br>RAD001 or matching placebo will be dispensed by the study center personnel on an outpatient basis. On the days of PK sampling, RAD001 or matching placebo will be administered by the investigator (or designee). Patients who enter the open-label phase of the study will be provided with an adequate supply of RAD001.<br>RAD001 or matching placebo will be dosed starting on Treatment Day 1 (Visit 2). Patients will be instructed to take 4.5 mg/m^2 (rounded to the nearest milligram) of RAD001 or matching placebo orally once daily at the same time every day immediately after a meal. RAD001 or matching placebo tablets should be swallowed whole with a glass of water. The tablets should not be chewed or crushed. If tablets can not be swallowed, the tablets should be disintegrated in approximately 30 mL of water. Immediately prior to administration, the contents should be stirred gently until the tablets have disintegrated into a suspension. The contents should then be drunk by the patient. Afterwards, the glass should be rinsed with an additional 30 ml of liquid and drunk by the patient.<br>Any dietary habits around the time of RAD001 or matching placebo intake should be as consistent as possible throughout the study, and in particular, during those periods when samples are being taken for pharmacokinetic analyses. If vomiting occurs, no attempt should be made to replace the vomited dose.<br>At visits when blood will be drawn, patients should not take the daily study drug dose **until after** blood is drawn so that an accurate trough level of RAD001 can be obtained. At these visits, patients should bring their daily dose of medication to the clinic with them for administration after the blood work is completed.<br>The initial dose of trial therapy will be 4.5 mg/m^2/day (either RAD001 or matching placebo), rounded to the nearest mg. A PK analysis will be conducted after 2 weeks by the central laboratory to assess the blood levels of RAD001. If well tolerated, the daily dose of RAD001 will be either maintained or increased so as to achieve a blood trough level of 5-15 ng/mL. In order to maintain the blind, the central laboratory conducting the PK analysis will upload the observed blood trough level of RAD001 for each patient into the IWRS. IWRS will then send an alert to the investigator advising either to maintain or increase the dose, depending on tolerability thus far and the observed RAD001 trough level. For placebo patients, IWRS will refer to a randomization list to arbitrarily recommend either maintaining or increasing the dose. The investigator will only consider maintaining or increasing the dose if the current dose is well tolerated. **Note:** If the blood trough level is above 15 ng/mL, IWRS will advise the investigator to reduce the dose to the next lowest dose level. Blood levels of RAD001 will be assessed 1-2 weeks after any dose increase to a new level or any decrease in an enzyme-inducing drug, or any

	increase in an enzyme-inhibiting drug. In order to maintain the blind, IWRS will also arbitrarily recommend reducing the dose of placebo patients who had previously been dose-escalated, using a randomization list as above.<br>Patients who are active at the time of Amendment 5 approval and consent to participate will be asked to have additional PK blood samples collected at any ONE of their regularly scheduled visits. Other dose adjustments (reduction, interruption or possible dose re-escalation to starting dose) according to safety findings will be allowed. Regular safety reviews by a Data Monitoring Committee (DMC) will be performed.<br>Table 6-4 provides the various dose levels, as guided both by safety and RAD001 levels. The starting dose is 4.5 mg/m^2. If a trough blood level of 5-15 ng/mL is not achieved with the current dose, then a dose increase according to Table 6-4 will be undertaken, **as tolerated**. If the current dose is not tolerated, if an adverse event mandating a dose decrease occurs (Table 6-1, Table 6-2 or Table 6-3), or if the trough level exceeds 15 ng/mL, then a dose decrease according to Table 6-4 will be undertaken. Patients on placebo will have dose adjustments recommended by IWRS, in a randomized fashion, in order to maintain the blind.<br>Fourteen days after dose increases to a new level or any decrease in an enzyme-inducing drug, or any increase in an enzyme-inhibiting drug, a trough PK analysis will be conducted and, provided that there are no safety issues, the dose will be escalated until the desired trough serum level of 5-15 ng/mL is achieved or until the maximum daily dose level of 14.22 mg/m^2 is reached.<br>Patients will receive treatment with study drug until SEGA progression, unacceptable toxicity, or until the investigator or patient decides that continuation is not in the best interest of the patient. For patients who are unable to tolerate the protocol-specified dosing schedule, dose adjustments are permitted in order to keep the patient on study drug. The guidelines set forth in Table 6-1, Table 6-2 and Table 6-3 should be followed.
**Visit schedule and assessments**	Refer to Table 7-1 and Table 7-2
**Efficacy assessments**	An MRI of the brain will be performed at screening/baseline, at 12, 24 and 48 weeks after start of study treatment, and annually thereafter. A CT/MRI of the kidney will also be performed at baseline, and will be repeated at the same time points as MRIs of the brain if any angiomyolipomata with longest diameter ≥ 1.0 cm were present at baseline. For each patient, the same imaging modality should be used throughout the trial. All imaging scans of the brain and kidney will be submitted for an Independent Central Radiology Review within 2 days of the scan.<br>Investigators should ensure the safety of patients with vagal nerve stimulators by following applicable guidance (e.g. http://www.fda.gov/MedicalDevices/Safety/AlertsandNotices/PublicHealthNotifications/ucm062125.htm)<br>Plasma angiogenesis markers (e.g., VEGF, basic FGF, PLGF, soluble VEGF receptor1 and soluble VEGF receptor2) will be measured at screening/baseline, at 4, 12, 24, 36, and 48 weeks after start of treatment, and at end of treatment to measure changes while on treatment and capture any change in angiogenic markers at end of treatment.<br>Frequency of seizures will be measured using data obtained at screening/baseline and at 24 weeks from a 24-hour video EEG. The severity of seizures will be measured, for patients being treated with antiepileptics at baseline, using data obtained from a Seizure Severity Questionnaire at screening/baseline and 24 weeks.

	Skin lesions, for patients with documented skin lesions at baseline, will be photographed using a digital camera at baseline and every 12 weeks thereafter and assessed, using the Physician's Global Assessment, after every 12 weeks of treatment.<br>Neuropsychological assessments will be performed at screening/baseline and 6 months after start of study treatment in an attempt to assess the natural course of cognitive function and other neuropsychological aspects, as well as the potential effect of RAD001. One of the following will be required for children ages two and older (according to patient's age at randomization and availability in patient's native language):<br>1. Wechsler Preschool and Primary Scale of Intelligence (WPPSI) - for children aged 2 to 5<br>2. Wechsler Abbreviated Scale of Intelligence (WASI) - for patients aged 6 and older.<br>If either of the above can not be performed due to cognitive/behavioral impairments or absence of assessment in patient's native language, the Vineland Adaptive Behavior Scale (VABS) should be completed by the patient's parent or caregiver.
**Safety Assessments**	Toxicity will be assessed by the NCI Common Toxicity Criteria for Adverse Events v3.0 (CTCAE) (available on http://ctep.cancer.gov/protocolDevelopment/electronic_applications/docs/ctcaev3.pdf). The NCI CTCAE has been chosen to maintain consistency with other RAD001 trials. Safety tests will include monitoring and recording of all AEs and SAEs at every visit, and monitoring of vital signs, physical condition and body height (using a stadiometer) and weight. Laboratory assessments include hematology and blood chemistry (including serum creatinine and calculated creatinine clearance) done every two weeks for the first 8 weeks, at weeks 12, 18 and 24, and every 12 weeks thereafter. For all patients, all available prebaseline height and weight data should be collected in order to adequately represent the patient's rate of growth prior to starting the study.<br>Hepatitis Screening<br>Prior to randomization, the following three categories of patients should be tested for hepatitis B viral load and serologic markers, that is, HBV-DNA, HBsAg, HBs Ab, and HBc Ab:<br>1. All patients who currently live in (or have lived in) Asia, Africa, Central and South America, Eastern Europe, Spain, Portugal, and Greece. [http://wwwnc.cdc.gov/travel/yellowbook/2010/chapter-2/hepatitis-b.aspx#849]<br>2. Patients with any of the following risk factors:<br>• known or suspected past hepatitis B infection,<br>• blood transfusion(s) prior to 1990,<br>• current or prior IV drug users,<br>• current or prior dialysis,<br>• household contact with hepatitis B infected patient(s),<br>• current or prior high-risk sexual activity,<br>• body piercing or tattoos,<br>• mother known to have hepatitis B<br>• history suggestive of hepatitis B infection, e.g., dark urine, jaundice, right upper quadrant pain.<br>• Additional patients at the discretion of the investigator<br>If a patient tests positive, they will be considered ineligible for the study according to

	Exclusion Criterion 6. Please note that patients who test negative for HBV-DNA, HBsAg, and HBc Ab but positive for HBs Ab with prior history of vaccination against Hepatitis B will be eligible. The fact that the patient had been vaccinated should be entered into the patient's Medical History CRF.<br>Patients with any of the following risk factors for hepatitis C should be tested using quantitative RNA-PCR:<br>• known or suspected past hepatitis C infection (including patients with past interferon 'curative' treatment),<br>• blood transfusions prior to 1990,<br>• current or prior IV drug users,<br>• current or prior dialysis,<br>• household contact of hepatitis C infected patient(s),<br>• current or prior high-risk sexual activity,<br>• body piercing or tattoos<br>At the discretion of the investigator, additional patients may also be tested for hepatitis C.<br>If a patient tests positive, they will be considered ineligible for the study according to Exclusion Criterion 6 according to Exclusion Criterion 6.<br>For patients who have already been randomized and received study drug prior to the approval of amendment 2, the same screening process should be followed at the patient's next visit. If the patient tests positive for Hepatitis B, the investigator should follow the guidelines according to Table 4-1 and Table 4-2. Please refer to Table 7-1 and Table 7-2 for HCV RNA-PCR monitoring schedule for those patients with positive HCV RNA-PCR baseline tests who do not meet the flare criteria outlined in Table 4-3.If the patient tests positive Hepatitis C, and the criteria for flare according to Table 4-3 are observed, trial therapy should be discontinued and further treatment is up to the investigators discretion.<br>All patients will have additional blood samples collected during screening and annually thereafter until their 10th birthday and every 12 weeks thereafter for endocrine assessment. These assessments include:<br>• Follicle Stimulating Hormone (FSH)<br>• Luteinizing Hormone (LH)<br>• Testosterone<br>• Estradiol (female patients)<br>In addition, to assess overall sexual development, all patients will be evaluated using Tanner Staging at Screening/baseline and then annually thereafter. Growth and development milestones; age of thelarche (females), age of adrenarche (males), date of menarche (females) should also be captured. Height using a stadiometer and weight will be assessed each time a physical exam is conducted according to Table 7-1 and Table 7-2.<br>Patients will be followed for safety until at least 28 days after study treatment discontinuation. Following these 28 days, any serious adverse events that are suspected to be related to the study drug and occur within the next 8 weeks (56 days) will also be collected.<br>Pneumonitis is a known side-effect of rapamycin analogues including RAD001. Clinically significant pneumonitis is typically accompanied by non-specific symptoms including dyspnea, nonproductive cough, fatigue, and fever. Diagnosis is generally suspected in individuals receiving mTOR inhibitors who develop these symptoms or in asymptomatic individuals in whom a routine chest CT scan reveals

	a new ground glass or alveolar infiltrate. The frequency of symptomatic pulmonary toxicity (all grades) was approximately 13% in a phase III study of RAD001 in patients with metastatic renal cell carcinoma ([CRAD001C2240]). Severe (CTC grade 3) pneumonitis occurred in 4% of patients, and an occasional fatality was reported. The lung toxicity was partly or completely reversible in the majority of cases with interventions including drug interruption, discontinuation and the use of corticosteroids.<br><br>Individuals participating in this trial will be questioned at each visit as to the presence of new or changed pulmonary symptoms consistent with lung toxicity. If an investigator suspects a patient may be developing pneumonitis investigations such as pulmonary function tests, chest CT and referral to a pulmonologist should be considered. For more detailed management advice see Table 6-3.
**Potential drug interactions**	Patients must be instructed not to take any additional medications (over-the-counter or other products) during the study without prior consultation with the investigator. All medications taken within 30 days of starting study treatment should be reported on the Concomitant Medication/Significant Non-drug Therapy Prior to Start of Study Drug CRF.<br><br>**With the exception of enzyme-inducing antiepileptic drugs, which are allowed on this study**, the following guidelines must be adhered to during the study:<br>• Co-administration with moderate or strong inhibitors of CYP3A4 or inhibitors of P-glycoprotein (PgP) must be avoided (refer to Table 6-5 and Table 6-6)<br>• Co-administration with strong inducers of CYP3A4, other than antiepileptics, must be avoided<br>• Seville orange, star fruit, grapefruit and their juices affect P450 and PgP activity. Concomitant use should be avoided<br>• Investigational or commercial anti-proliferative agents other than study drug (including other mTOR inhibitors, e.g., sirolimus, temsirolimus) are prohibited.<br><br>Lists of known medications with effects on CYP3A and PgP are included Table 6-5 and Table 6-6, respectively.RAD001 may affect the response to vaccinations making the response to the vaccination less effective. Live vaccines should be avoided while a patient is treated with RAD001.<br><br>Otherwise, the use of other concomitant medication/therapy deemed necessary for the care of the patient is allowed. The investigator should instruct the patient to notify the study site about any new medications he/she takes after the start of the study drug. All medications (other than study drug) and significant non-drug therapies (including physical therapy and blood transfusions) administered after the patient starts trial therapy must be listed on the Concomitant Medications/Significant Non-drug Therapies CRF.
**PK**	A 2-mL, pre-dose blood sample will be collected for determination of RAD001 trough blood levels (C_{min}) starting on Visit 3 (week 2) and every visit thereafter. In addition, 1-2 weeks after every dose increase (if the patient is naïve to that dose), a trough sample and an additional 2-mL sample will be collected for determination of PK 2.0 hours (±30 mins, C_{2h}) after trial therapy dose administration.<br><br>In addition, 1-2 weeks following any decrease of a CYP3A4 or PgP inducer or initiation or increase of a CYP3A4 or PgP inhibitor a 2-mL trough sample and a 2-mL sample will be collected for determination of PK 2.0 hours (±30 mins, C_{2h}) after trial therapy dose administration.<br><br>Patients who are active at the time of Amendment 5 approval and consent to participate will be asked to have additional 2 mL samples collected if 10 years or older and 1 mL samples collected for younger patients. PK samples will be collected at steady-state condition at pre-dose and at 0.5, 1, 2, 5 and 24 hours after the patient's dose on any ONE of the regularly scheduled study visit days. The

	originally planned pre-dose PK sample and the 2-hour post-dose PK sample, (if required) will not be collected at this visit day. Meal intake guidelines for patients participating in the full PK profile should be followed.
**Biomarker assessments**	Plasma collection will occur at the following time points (unless local or national regulations do not permit): screening/baseline, 4, 12, 24, 36 and 48 weeks, and end of treatment. All patients' plasma samples (3 mL of blood) will be examined for RAD001 effects on angiogenesis markers (e.g., basic FGF, VEGF, PLGF, soluble VEGF receptor1, and soluble VEGF receptor2). A 3-mL pre-dose sample of whole blood will be collected for determination of mutations associated with TSC1 and TSC2 genes.
**DMC/Study Steering Committee**	A Data Monitoring Committee (DMC) will be constituted to oversee the safety of patients on this trial; the same DMC will be used in any other Novartis-sponsored trials evaluating RAD001 in patients with TSC-related diseases. The DMC will consist of at least two clinicians with expertise in TSC and one statistician. The DMC will be constituted prior to the randomization of the first patient in any of the Novartis-sponsored trials of RAD001 in TSC. The first safety review will be performed by the DMC approximately six months after the first patient in any of the Novartis-sponsored RAD001 trials in TSC has been randomized, and every six months thereafter, unless otherwise requested by the Chairman of the DMC. No interim analysis is planned. Recruitment will not be interrupted unless otherwise requested by the Chairman of the DMC. In addition, the DMC will receive safety updates on a regular basis.<br>A Study Steering Committee (SSC) will also be constituted to oversee the conduct of the study and making any necessary recommendations as appropriate. The committee will also develop study-related publications in accordance with the Novartis publication and authorship policy. The committee will be appointed by Novartis and will include two principal investigators from this trial, Novartis staff and possibly other clinical experts (after consultation with the SSC members). The committee will be chaired by one of the two Principal Investigators.
Statistical methods and data analysis	**Populations**<br>The Full Analysis Set (FAS) will consist of all randomized patients. The patients in the FAS will be analyzed in the treatment group they were assigned to at randomization.<br>The Per Protocol Set (PPS) will consist of all patients from the Full Analysis Set without any major protocol deviation, who are evaluable for efficacy and who have completed a minimum exposure requirement. However, if a patient progressed, discontinued due to an adverse event or died before the minimum exposure requirement could be met, or before he/she could become evaluable for efficacy, that patient will still be included in the Per Protocol Set. Patients will be evaluable for efficacy if they have a known SEGA response status. The minimum exposure requirement is defined as having received study treatment on at least 50% of the days in the first 12 weeks since the first day of treatment. The PPS will be used for supportive analyses of the primary endpoint.<br>The safety population will consist of all patients that received at least one dose of the study medication and had at least one post-baseline safety assessment. Patients will be analyzed according to treatment received. Note: The statement that a patient had no adverse events (on the Adverse Events CRF) constitutes a safety

	assessment.<br>**Primary analysis**<br>The primary analysis will be a comparison of the SEGA response rates in the RAD001 and placebo arms using a one-sided exact Cochran-Mantel-Haenszel (CMH) test at the 2.5% level, analyzed in the Full Analysis Set (FAS). The analysis will be performed using a data cut-off defined as 6 months after the last patient is randomized in the trial.<br>**Interim analysis**<br>Not planned.<br>**Statistical considerations**<br>SEGAs are slow growing lesions. Spontaneous regression without surgical or drug intervention has not been reported. Reduction in volume, relative to baseline assessment, will be considered treatment-related. As there is no approved treatment for SEGAs, a placebo-controlled randomized design is a logical choice for evaluating a new treatment.<br>Because of their slow progressing nature, response rate is an ideal outcome parameter. MRI imaging will be used to evaluate changes in tumor dimensions because of the high accuracy of measurement and spatial resolution and the absence of radiation associated with the technique.<br>**Statistical power and rationale for sample size**<br>The primary analysis compares SEGA response rate between the two treatment arms using an exact CMH test. The randomization is unbalanced, with two patients allocated to RAD001 for every one patient allocated to placebo. As there are no reported cases of tumor regression in patients with SEGA, the response rate on placebo is expected to be close to 0%. The SEGA response rate on RAD001 is hoped to be at least 20%. It is planned to use a one-sided test and a 2.5% significance level. Simulation was used to obtain a sample size of 99 patients (66 randomized to RAD001 and 33 randomized to placebo), which will provide 93% power to detect a treatment difference from 0% on placebo to 20% on RAD001.<br>**Pharmacokinetic analyses**<br>RAD001 trough (C_{min}) and 2.0 hour (±30 mins, C2h) levels will be summarized by means of descriptive statistics and used in future analyses along with RAD001 trough levels and steady-state full profiles from other studies, with the goal to compare the results from this protocol with an appropriate reference population (e.g., PK data from other RAD001 monotherapy protocols in patients with TSC-related conditions).<br>For the PK profile collection implemented as part of Amendment 5, the analysis will estimate the impact of age, weight, BSA, and co-administration of CYP3A4/PgP enzyme inducers on the PK of everolimus (as measured by CL/F, C_{max}, T_{max}, C_{min} and AUC) in children.<br>For each PK parameter except T_{max}, the ratio (and 90% CI) between the reference age group (≥18 years old) and the other age groups will be estimated by a linear model with log-transformed PK parameter as the dependent variable and age group as a categorical variable. The same model will be used to assess the impact of the other factors, including co-administration of CYP3A4/PgP inducers, weight and BSA (as categorical variable), restricting to the samples collected in patients aged less than 18 years. The model may be adjusted for other factors if appropriate. Categories of BSA and weight will be defined in the RAP.<br>If the distribution of age, weight and BSA does not allow estimation of ratios for comparisons of interest, for each factor separately, a regression model with each of these factors as a continuous variable (with transformation if appropriate) may be

	used to explore the relationship between PK parameters and each of these factors separately. The model may also be adjusted for other factors if appropriate.<br><br>Furthermore, a regression model including all the factors may be fitted to explore their joint effect, if appropriate considering data distribution and co-linearity between the factors. Population PK modeling may also be carried out to further explore their effects in pediatric population, in contrast to that in adult population.<br><br>All PK parameters, including T_{max} will be summarized by categories of age, weight, BSA, and co-administration of CYP3A4/PgP enzyme inducers.

1 Background

1.1 Overview of Tuberous Sclerosis Complex (TSC) and Subependymal Giant Cell Astrocytomas (SEGA)

TSC is an autosomal dominant genetic disorder caused by inactivating mutations in the TSC1 or TSC2 genes, and characterized by benign, highly vascular, hamartoma growth. Lesions occur in the brain, kidneys, heart, liver, lungs and skin, leading to seizures and epilepsy, mental retardation, autism, and renal and pulmonary complications (Gomez et al 1999; Astrinidis and Henske 2005; Inoki et al 2005; Kwiatkowski and Manning 2005). Measures of childhood prevalence range from 1 in 6,800 to 1 in 17,300 but full ascertainment is difficult to achieve (Yates 2006). Brain lesions are the primary cause of morbidity and mortality in this disorder in childhood.

The incidence of subependymal giant cell astrocytoma (SEGA) in TSC varies from 5 to 15% (Shepherd et al 1991). SEGA lesions are always usually associated with TSC. They arise in the subependymal layer of the lateral ventricle and are usually located near the foramen of Monro and enhance homogeneously with contrast on MRI with no evidence of surrounding edema. These are slowly growing lesions that are typically unapparent clinically until they reach sufficient size to produce ventricular obstruction and hydrocephalus. By the time symptoms are noted they are often irreversible even by emergent surgical intervention. They arise deep within the brain in the region of the foramen of Monro, which hampers their surgical resection, as the approach to the lesion entails removal of substantial amounts of viable cerebral tissue. Surgery, even when successful, often results in significant morbidity.

For SEGAs that exhibit serial growth, cause hydrocephalus, or produce any clinical symptoms, resection is currently the only recommended treatment for these patients (Torres et al 1998; Sinson et al 1994; Cuccia et al 2003). The lack of reported spontaneous regression or subsequent stabilization in SEGAs supports this recommendation (Franz 2007). In addition, SEGAs do not typically respond to radiation therapy or chemotherapy (Franz 2007). Given the genetic basis of tuberous sclerosis, the risk of inducing second malignancies through utilization of standard chemotherapeutic agents or radiation therapy has been noted (Matsumura et al 1998).

In the SEGA population, antiepileptic drugs are often used in an attempt to control the frequency of epileptiform events, but this class of drugs does not address the underlying pathology and has no effect on tumor burden. Mortality associated with TSC in animal models is not changed by controlling seizures (Erbayat-Altay et al 2007).

The TSC1/TSC2 protein complex is a negative regulator of the mTOR pathway. Hence, mutation or loss of either of these gene products in preclinical models is associated with increased mTOR pathway activation and heightened sensitivity to mTOR inhibitors (Astrinidis and Henske 2005; Inoki et al 2005; Kwiatkowski and Manning 2005).

mTOR pathway upregulation has also been observed in lesions derived from TSC patients (Astrinidis and Henske 2005; Kwiatkowski and Manning 2005), and TSC1 or TSC2 defective experimental animal models exist which recapitulate the pathology, behavioral and neurological aspects of the tuberous sclerosis disease (Onda et al 1999; Kwiatkowski and

Manning 2005; Uhlmann et al 2002; Kenerson et al 2005) and are sensitive to mTOR inhibition (Kenerson et al 2005; Astrinidis and Henske 2005; Kwiatkowski and Manning 2005). In this respect, experiments are in progress aimed at analyzing the effects of RAD001 in animal models of TSC (D. Kwiatkowski / Novartis collaboration). Preliminary data indicate that gross kidney lesion scores can be significantly reduced by RAD001 treatment in both TSC1+/- and TSC2+/- mouse models (p = 0.02 compared to untreated controls, unpaired t-test). Moreover, this reduction is associated with a dramatic inhibition of the phosphorylation of the ribosomal S6 protein in treated kidney lesions (S6 phosphorylation is an established pharmacodynamic marker of mTOR pathway activation status). Furthermore, a dramatic improvement in survival has been observed in a mouse brain model of TSC (genotype : $Tsc1^{cc}$ $syn\text{-}cre^{+}$), with a highly statistically significant improvement in survival ($p < 0.0001$) associated with RAD001 treatment. The investigator also noted an improvement in behavior, weight gain and neurological phenotype. Assessment of brain pathology is currently ongoing.

Finally, both estrogen and vascular endothelial growth factor (VEGF) signaling have been implicated in the pathogenesis and vascularization of TSC lesions (Astrinidis and Henske 2005; Kwiatkowski and Manning 2005). In this respect, RAD001 has been shown to inhibit both estrogen and VEGF-dependent signaling events (Boulay et al 2005; O'Reilly et al 2005). Taking all these data into account, therefore, there is a strong rationale for using RAD001 for the treatment of patients with tuberous sclerosis.

With a manageable safety profile, RAD001 may significantly reduce morbidity associated with SEGAs, based on the hypothesis that it is capable of restoring the signaling balance of TSC/mTOR-mediated pathways, and hence inhibit growth or cause regression of SEGAs in patients with TSC.

1.2 Volumetric assessment of tumor response

Assessment of tumor response to therapy is a critical component for drug development and for the clinical management of patients. Current approaches for classifying tumor response are based on anatomical measurements in either one dimension (Response Evaluation Criteria in Solid Tumors [RECIST] (Therasse et al 2000)) or two dimensions (World Health Organization [WHO] (Miller et al 1981)). However, response rates as determined from these criteria may not always be sufficiently accurate.

As tumors grow in three dimensions, shrinkage can thus be accurately defined as a decrease in tumor volume. RECIST and WHO measurements are essentially surrogates for volume. With developing state-of-the-art imaging techniques providing a 3-D information set and computer algorithm development, it is now possible to obtain accurate and true tumor measurements using volume (Twombly 2006), rather than only one or two dimensional measurements. Also, for the changes in uni- and bi-dimensional measurements to be a good surrogate for changes in volume, one should assume that target lesions are spherical in shape, which may not be true for all tumor types.

In a recent study using serial MRI examinations for 60 patients with cervical cancer (Mayr et al 2006), the researchers evaluated the tumor shape and its temporal change during radiation therapy in cervical cancer and the effect of tumor configuration changes on the correlation between region of interest based (ROI-based) and diameter-based MRI tumor measurement. The researchers reported that most cervical cancers (70%) are not oval in shape

and they become increasingly irregular during and after therapy because of non-concentric tumor shrinkage. The authors concluded that three-dimensional volumetry, which can optimally measure irregular volumes, may provide better response assessment during treatment than diameter-based measurement.

A recent breast cancer study (Partridge et al 2005) assessed the value of MRI measurements of breast tumor volume for predicting recurrence-free survival (RFS) in patients undergoing neoadjuvant chemotherapy and compared the predictive value of MRI assessments with that of established prognostic indicators. This study included 62 patients and the longest diameter and volume of each tumor were measured on MRI before and after each cycle of chemotherapy. Univariate Cox model analysis showed initial MRI volume was the strongest predictor of RFS ($p = 0.002$). Final change in MRI volume ($p = 0.015$) was more predictive than change in diameter on MRI ($p = 0.077$) or clinical examination ($p = 0.27$). Multivariate analysis showed initial MRI volume ($p = 0.005$) and final change in MRI volume ($p = 0.003$) were significant independent predictors. The authors concluded that MRI tumor volume was more predictive of RFS than tumor diameter, suggesting that volumetric changes measured using MRI may provide a more sensitive assessment of treatment efficacy.

Finally, in a public workshop on brain tumor clinical trial endpoints organized by the FDA, AACR and ASCO on January 20, 2006, it was recognized that measuring tumor diameter is probably an outdated methodology, as small percentage changes in diameter can reflect much larger changes in tumor volume. It was also mentioned that both manual and automated segmentation techniques provide more accurate measurements of tumor volume than diameter measurement. In addition, the regional distribution of the lesion was considered an important issue; a 1-mm reduction in tumor volume in a certain part of the brain might have a dramatic clinical effect whereas a larger volume reduction elsewhere in the brain might be clinically meaningless. The latter comments are of particular importance in the SEGA trial, with SEGA lesions being located close to the foramen of Munro with the potential of causing hydrocephalus.

In summary, volumetric assessment of target lesions using MRI will allow a more accurate assessment of lesion response to treatment and will provide a better approximation of actual tumor volume.

1.3 Overview of RAD001

1.3.1 RAD001

RAD001 (everolimus) is a novel derivative of rapamycin. RAD001 has been in clinical development since 1996 as an immunosuppressant in solid organ transplantation. In 2003, RAD001 was approved in Europe (trade name: Certican®) via the Mutual Recognition Procedure (MRP) for the prevention of organ rejection in patients with renal and cardiac transplantation. Certican® is also approved in Australia, South Africa, the Middle East, Central and South America, the Caribbean and some Asian countries. In 2010, RAD001 was approved in the United States under the trade name: Zortress® for the prevention of organ rejection in adults receiving kidney transplants who are at a low to moderate immunologic risk. In addition, RAD001 received accelerated approval in the United States under the trade name Afinitor® for the treatment of patients with subependymal giant cell astrocytoma

(SEGA) associated with tuberous sclerosis (TS) who require therapeutic intervention but are not candidates for curative surgical resection.

Table 1-1 Active drug substance

Chemical name:	(1R,9S,12S,15R,16E,18R,19R,21R,23S,24E,26E,28E,30S,32S,35R)-1,18-dihydroxy-12-(1R)-2-[(1S,3R,4R)-4-(2-hydroxyethoxy)-3-methoxycyclohexyl]-1-methylethyl}-19,30-dimethoxy-15,17,21,23,29,35-hexamethyl-11,36-dioxa-4-aza-tricyclo[30.3.1.0^{4,9}]hexatriaconta-16,24, 26,28-tetraene-2,3,10,14,20-pentaone
International non-proprietary name	Everolimus

Everolimus (Afinitor®) entered clinical development for numerous oncology indications in 2002. Afinitor was granted approval in the United States on 30-Mar-2009 for the treatment of patients with advanced renal cell carcinoma (RCC) after failure of treatment with sunitinib or sorafenib. Afinitor was approved by the European Commission on 03-Aug-2009. As of 07-Jan-2010, Afinitor is also approved in Argentina, Australia, Brazil, Canada, Chile, Costa Rica, Iceland, India, Malaysia, New Zealand, Norway, Singapore, South Korea, Switzerland, Uruguay and Venezuela. Applications are pending in various other countries worldwide.

Approximately 2686 patients with various malignancies have been treated in either Novartis-sponsored or non-Novartis-sponsored clinical studies as of 31Aug2007. Overall, Novartis sponsored a total of 24 studies with RAD001 administered either as a single-agent (n=947), in combination with other anti-tumor agents (n=664), and two studies of healthy volunteers (n=127).

RAD001 is being investigated as an anticancer agent based on its potential to act:

- directly on the tumor cells by inhibiting tumor cell growth and proliferation
- indirectly by inhibiting angiogenesis leading to reduced tumor vascularity (via potent inhibition of tumor cell VEGF production and VEGF-induced proliferation of endothelial cells)

1.3.2 The role of mTOR pathway tumorigenesis

An important aspect of the antitumor effect of RAD001 is its potential to act on both tumor cells directly (to inhibit growth) and indirectly (by inhibiting angiogenesis). The observation of *in vivo* sensitivity of xenografts comprised of cells demonstrating resistance to RAD001 *in vitro* is attributed to the drug's potential to act on the vascular component of the supporting peritumoral stroma. The anti-angiogenic property of RAD001 has been confirmed through experiments demonstrating the effect of RAD001 in countering VEGF-induced proliferation of human umbilical vein endothelial cells (HUVECs) *in vitro*, VEGF-driven angiogenesis in a chamber implant murine model and neovascularization in a murine orthotopic melanoma model.

At the cellular and molecular level, RAD001 acts as a signal transduction inhibitor. The target of RAD001 is mTOR (mammalian target of rapamycin), a serine-threonine kinase that is a member of the larger PI3K (phosphatidylinositol 3-kinase) family and present in all cells. RAD001 selectively inhibits mTOR which regulates cell growth, proliferation and survival.

The mTOR kinase is mainly activated via the phosphatidylinositol 3-kinase (PI3K) pathway through AKT/PKB and the tuberous sclerosis complex (TSC1/2). Mutations in these components or in PTEN, a negative regulator of PI3 kinase, may result in their dysregulation. Abnormal functioning of various components of the signaling pathways contributes to the pathophysiology of numerous human cancers. Various preclinical models have confirmed the role of the P13K/AKT pathway in tumor development (Cohen 2005).

The main known functions of mTOR include (Bjornsti 2004):

- mTOR functions as a sensor of mitogens, growth factors, energy and nutrient levels, facilitating cell-cycle progression from G1 - S phase in appropriate growth conditions.
- The PI3K (mTOR) pathway itself is frequently deregulated in many human cancers, and oncogenic transformation may sensitize tumor cells to mTOR inhibitors.
- The mTOR pathway is involved in the production of pro-angiogenic factors (e.g. VEGF) and endothelial cell growth and proliferation.
- Through inactivating eukaryotic initiation factor 4E binding proteins and activating the 40S ribosomal S6 kinases (e.g., p70S6K1), mTOR regulates protein translation.

The regulation of mTOR signaling is complex and involves positive regulators, such as AKT, that phosphorylate and inactivate negative regulators such as the Tuberous Sclerosis Complex (TSC1/TSC2).

The PI3K/AKT/mTOR pathway is known to be dysregulated in numerous proliferative disorders including cancer. Molecular epidemiological studies have also shown that activation of the PI3K/AKT/mTOR pathway is frequently associated with worsening prognosis through resistance to treatment, disease extension and disease progression. A variety of preclinical models have confirmed the role of this pathway in tumor development. It has also been demonstrated that constitutional activation of kinases such as AKT can lead to inexorable development of cancers resembling those characterized by frequent activation of the same kinases. This is complemented by the demonstration of the antitumor activity of kinase inhibitors acting on the pathway in *in vitro* and *in vivo* preclinical models.

1.3.3 Preclinical studies

RAD001 inhibits the proliferation of a range of human tumor cell lines *in vitro* including lines originating from lung, breast, prostate, colon, melanoma and glioblastoma. RAD001 also inhibits the proliferation of human umbilical vein endothelial cells (HUVECs) *in vitro*, with particular potency against VEGF-induced proliferation suggesting that RAD001 may also act as an anti-angiogenic agent. The anti-angiogenic activity of RAD001 was confirmed *in vivo*. RAD001 selectively inhibited VEGF-dependent angiogenic response at well tolerated doses. Mice with primary and metastatic tumors treated with RAD001 showed a significant reduction in blood vessel density when compared to controls.

RAD001 administered daily p.o. was a potent inhibitor of tumor growth, at well tolerated doses, in 11 different mouse xenograft models (including pancreatic, colon, epidermoid, lung and melanoma) and two syngeneic models (rat pancreatic and mouse orthotopic melanoma). These models included tumor lines considered sensitive and “relatively resistant” *in vitro*. In general, RAD001 was better tolerated in mouse xenograft models than standard cytotoxic agents (i.e., doxorubicin and 5-fluorouracil), while possessing similar anti-tumor activity.

Additionally, activity in a VEGF-impregnated s.c. implant model of angiogenesis and reduced vascularity (vessel density) of RAD001-treated tumors (murine melanoma) provided evidence of *in vivo* effects of angiogenesis.

All significant adverse events observed in toxicology studies with RAD001 in mice, rats, monkeys and mini-pigs were consistent with its anticipated pharmacological action as an anti-proliferative and immunosuppressant, and were at least in part reversible after a 2- or 4-week recovery period with the exception of the changes in male reproductive organs, most notably testes.

Brain penetration

RAD001 penetration into brain tissue has been evaluated in mice and rats. Non-tumor bearing BALB/c mice were administered RAD001 once at 5 mg/kg orally or 1 mg/kg intravenously, and blood and tissue samples were obtained at various times after drug administration. The extent of penetration (AUC_{brain}/AUC_{blood}) was 1.8% and 5.2% after oral and intravenous administration, respectively. Despite the relatively low penetration, RAD001 brain tissue concentrations (69 ng/g after oral; 8 ng/g after intravenous) are within range of the IC_{50} values for PTEN-/- glioblastomas for at least 9 hours and above the IC_{50} values for PTEN+/+ mice for at least 2 hours [Report RD-2002-03252], [Report DMPK (CH) R00-2214].

In Wistar rats, the brain RAD001 distribution was related to drug dose and time after intravenous drug administration. At RAD001 doses up to 1 mg/kg, the blood and brain tissue concentrations increased linearly; thereafter, a non-linear increase was noted in both. The higher RAD001 brain tissue concentrations at higher intravenous doses are consistent with a saturation of an efflux pump present in brain capillary endothelial cells. The kinetics of RAD001 brain uptake were established by measuring blood and brain tissue concentrations of ^{3}H RAD001 at various times after an intravenous bolus dose. RAD001 was rapidly distributed in the brain with slow efflux. At 168 hours after the dose, significant brain levels of 6 ng/g were detected without any significant blood levels. Although up to 24 hours no metabolites are noted in the brain, at 168 hours they represent 60% of total drug in the brain [Report DMPK (CH) R00-2214].

Although RAD001 penetration into the brain is low, after a 1.0-mg intravenous dose of RAD001 in Wistar rats, brain tissue concentrations exceeded the *in vitro* antiproliferative IC_{50} for HUVEC cells and a panel of PTEN-/- glioblastoma cell lines for 168 hours, and selected PTEN+/+ cell lines for 24 hours [Report RD-2001-00852, Report RD-2002-03252].

1.3.4 Clinical experience

1.3.4.1 Pharmacokinetics

The pharmacokinetic characteristics of RAD001 have been extensively investigated in the context of the drug's development as an immunosuppressant in solid organ transplantation where RAD001 was administered twice daily as a part of an immunosuppressant multi-drug regimen consistently including cyclosporine A and glucocorticoids. Recent phase I studies provide steady-state pharmacokinetics for both the weekly and daily schedules at varying dose levels in patients with advanced cancers.

RAD001 is rapidly absorbed after oral administration, with a median time to peak blood levels (t_{max}) of 1-2 hours post dose. The extent of absorption is estimated at above 11%. The area under the blood concentration-time curve (AUC) is dose-proportional over the dose range tested while maximum blood concentration (C_{2h}) appears to plateau at dose levels higher than 20 mg. The terminal half-life ($t_{1/2}$) in cancer patients averaged 30 hours, similar to that in healthy subjects. Inter-patient variability is moderate with a coefficient of variation (CV) of approximately 50%. A high-fat meal altered the absorption of RAD001 with a 1.3-hour delay in t_{max}, a 60% reduction in C_{2h}, and a 16% reduction in AUC. In whole blood, approximately 80% of RAD001 is contained in red blood cells. Of the fraction of drug contained in plasma, 74% is protein-bound. The apparent distribution volume (Vz/F) after a single dose was 4.7 L/kg. RAD001 is eliminated by metabolism, mainly by hydroxylation, then excreted into the feces at >80%.

RAD001 is mainly metabolized by CYP3A4 in the liver and to some extent in the intestinal wall. RAD001 is also a substrate of P-glycoprotein (P-gp). Therefore, absorption and subsequent elimination of systematically absorbed RAD001 may be influenced by medicinal products that interact with CYP3A4 and/or P-glycoprotein. *In vitro* studies showed that RAD001 is a competitive inhibitor of CYP3A4 and of CYP2D6 substrates, potentially increasing the concentrations of medicinal products eliminated by these enzymes. In two phase III clinical trials in patients following kidney transplantation, strong inhibitors of CYP3A4 (azoles, antifungals, cyclosporine, erythromycin) have been shown to reduce the clearance of RAD001 therapy thereby increasing RAD001 blood levels. Similarly, Rifampin, a strong inducer of CYP3A4, increases the clearance of RAD001 thereby reducing RAD001 blood levels. Caution should be exercised when co-administering RAD001 with CYP3A4 inhibitors or inducers.

Patients with SEGA are often treated with P450 enzyme-inducing antiepileptic drugs (EIAEDs) which lead to an increase of RAD001 apparent clearance. Commonly prescribed EIAEDs that are used in this population include:

1. Phenytoin (Dylantin®, Dilantin Kapseals®, Dilantin® Infatabs®, Eptoin®, Epanutin®, Diphenin®, Dipheninum®, Phenytek®)
2. Mephenytoin (Mesantoin®)
3. Carbamazepine (Tegretol®, Biston®, Calepsin®, Carbatrol®, Epitol®, Equetro®, Finlepsin®, Sirtal®, Stazepine®, Telesmin®,Teril®, Timonil®, Trimonil®, Epimaz®, and Degranol®)
4. Phenobarbital (Luminal®)
5. Pentobarbital (Nembutal®)
6. Primidone (Mysoline®)
7. Oxcarbazepine (Trileptal®)

Patients receiving EIAEDs show decreased plasma levels of several medications when co-administered at conventional doses. This may, in turn, lead to ineffective dosing.

RAD001 pharmacokinetics in transplant patients was investigated in special populations such as subjects with hepatic or renal impairment, various ethnic groups and pediatric renal transplant patients. In subjects with mild or moderate hepatic impairment, mean AUC of RAD001 is increased 2-fold while renal impairment does not affect the pharmacokinetics of RAD001. Age, weight (both over the adult range) and gender do not affect the

pharmacokinetics of RAD001 to any clinically relevant extent. Also, pharmacokinetics is not altered in Asian patients whereas black patients have a 21% higher clearance compared to non-blacks. A single, escalating-dose study in Japanese subjects did not show a significant difference in dose-normalized systemic exposure.

The pharmacokinetic parameters derived for RAD001 given daily are summarized in Table 1-2.

Table 1-2 Steady-state RAD001 pharmacokinetics (daily dosing)

Parameter	5 mg	10 mg
N	4	6
t_{max} (h)	1 (1)	1 (1-6)
C_{min}^{ss} (ng/mL)	5.4 ± 1.8	13.2 ± 7.9
C_{max}^{ss} (ng/mL)	32 ± 9	61 ± 17
C_{max}^{ss}/Dose (ng/mL/mg)	6.4 ± 1.8	6.1 ± 1.7
$AUC\tau^{ss}$ (ng·h/mL)	238 ± 77	514 ± 231
$AUC\tau^{ss}$/Dose (ng·h/mL/mg)	48 ± 15	51 ± 23
C_{avg}^{ss} (ng/mL)	9.9 ± 3.2	21.4 ± 9.6

Values are median (range) for t_{max} and mean ± standard deviation for all others.

Dose-normalized parameters are per mg. τ is 24 h

1.3.4.2 Pharmacodynamic studies

Pharmacokinetic/pharmacodynamic modeling based on inhibition in a peripheral biomarker (S6 kinase inhibition in peripheral blood mononuclear cells) suggests that 10 mg daily should be an adequate dose to produce a high-degree of sustained target inhibition. Furthermore, molecular pharmacodynamic (MPD) studies using immunohistochemistry (IHC) in biopsied tumor tissue assessed the degree of inhibition and its duration (for p-S6, p-4E-BP1 and p-Akt expression) with the daily and weekly dosing. The pathologist was blinded for the biopsy sequence and found there was almost complete inhibition of p-S6 at all doses and schedules studied (p=0.001). Preliminary results suggest a dose-related decrease in p-4E-BP1 and increase in p-Akt expression with maximal effect at 10 mg daily and ≥ 50 mg weekly.

In [Study C2107], molecular changes were subsequently investigated through serial biopsying of tumors before and while on treatment (Tabernero et al 2005). Biopsying of tumors on treatment took place at week 4 of treatment (pharmacokinetic steady-state). All patients underwent a 24-hr post-dose biopsy. Patients following the weekly regimen had a further biopsy on Day 4-5 during the same week. Molecular activity was measured by IHC. In the absence of a reliable technique for measuring mTOR phosphorylation itself, the phosphorylation status of downstream markers S6 and eIF4G, for which reliable antibodies exist, was selected as reflecting the immediate pharmacodynamic effect of RAD001. Also measured were changes in the phosphorylation status of upstream AKT and the proliferation index Ki67. The daily regimen was associated with a high inhibition of p-S6 and p-eIF4G at 5mg/d and a complete inhibition at 10 mg/d. In patients on the weekly regimen, p-S6 inhibition was complete and sustained at all dose levels while that of p-eIF4G was complete and sustained at 50 mg/d but not at 20 mg/wk. On both regimens numerous patients demonstrated apparent up-regulation of AKT which tended, however, not to persevere in

patients at 50 mg/wk. The proliferation index was reduced in most patients, recovering in some of those on the 50 mg/wk regimen.

1.3.5 Rational for targeted dose

A concern regarding the use of RAD001 in oncology is the potential for immunosuppression during treatment. Consequently, in addition to individualizing the RAD001 dose according to the patient's body surface area, this protocol requires the upward titration of the dose from 4.5 mg/m^2, subject to tolerability, with the objective of achieving trough RAD001 levels in the range of 5-15 ng/mL. In this study, patients were treated with everolimus from a starting dose of 3 mg/m^2 with subsequent upward titration to attain a RAD001 trough in the range 5-15 ng/mL. This regimen was efficacious (32% of patients had >50% shrinkage at 6 months), and showed acceptable tolerability.

The starting dose of 4.5 mg/m^2 in M2301 is higher, as separate data from a phase I pediatric oncology study (Fouladi et al 2007) concluded that the maximum tolerated dose of everolimus in children is 5 mg/m^2. In order to optimize patient safety, and to ensure that excessive exposure does not occur, regular trough monitoring is carried out throughout the study.

In addition, the randomization in this trial will be stratified by the use of EIAEDs at baseline (yes *versus* no) in an attempt to minimize any imbalance between the two treatment arms.

1.3.6 Safety in phase I to III oncology studies

Data are available from phase I clinical studies of RAD001 given as a single agent to 147 patients with advanced solid tumors. Such studies included various doses and regimens (weekly dosing range, 5-70 mg, and daily dosing 5-10 mg). Approximately 46% of patients reported rash or erythema and 40% of patients presented with stomatitis/mucositis. The most frequent adverse events suspected to be drug-related in three studies using RAD001 as a single agent are listed in Table 1-3.

Table 1-3 Adverse events suspected to be drug-related in greater or equal to 10 percent of patients with advanced cancers reported in phase I RAD001 monotherapy [Studies C2101, C2102, C2107]

	Weekly			Daily		
	**5-30 mg n=30**	**50 mg n=18**	**70 mg n=38**	**5mg n=16**	**10 mg n=45**	**Total n=147**
No. Patients with AEs						
Any event	23 (1)	17 (2)	38 (10)	14 (1)	43 (14)	135 (28)
By event						
Rash	5	8	18	10	27 (1)	68 (1)
Stomatitis/mucositis	6	8 (2)	16 (2)	6 (1)	23 (3)	59 (8)
Fatigue	8	7 (1)	14 (1)	1	17 (1)	47 (3)
Nausea	5	4	8	2	18 (1)	37 (1)
Anorexia	1	6	10	3	15	35
Diarrhea	1	7	7	-	9	24
Vomiting	4	5	5	-	10	24
Headache	7	4	6	6	4	20
Pruritus	2	1	6	3	4	16
Infections[1]	1	3	3 (1)	1	6 (2)	14 (3)
Constipation	-	1	2	2	9	14

The numbers of patients (by dose level and dose schedule) who have reported grade 3 toxicities is given in parentheses .

[1] Infections noted as drug-related included:

Herpes simplex:	5 pts (1 at 50 mg/wk; 1 at 5 mg/d; 3 at 10 mg/d)
Oral candidiasis:	5 pts (1 at 50 mg/wk; 3 at 70 mg/wk; 1 at 10 mg/d)
Pneumonia (gr3)	1 pt (10 mg/d)
Pustular rash	1 pt (20 mg/wk)
Rhinitis	2 pts (50 mg/wk)
URTI	1 pt (50 mg/wk)
Urinary Tract Infection	1 pt (50 mg/wk)

Source: [Studies C2101], [C2102], [C2107]

Reduced blood cell counts at the initiation of treatment are frequent but remain mostly within the normal range or limited to grade 1; however, more serious neutropenia and thrombocytopenia was occasionally reported. This suggest that some patients may be particularly sensitive to the myelosuppressive effect of RAD001 making it necessary to monitor carefully blood cell counts at initiation of treatment.

Metabolic changes, particularly hyperlipidemia and hyperglycemia, may be observed during treatment with RAD001, and both events may be medically managed. Hyperlipidemia has been reported as an adverse drug reaction (ADR) in 10% of patients although review of the laboratory values suggests that as many as a quarter of patients develop grade 1-2 hyperlipidemia on treatment, mostly hypercholesterolemia. Hyperglycemia has been reported as an ADR in 7% of patients. Grade 3 hyperglycemia has been observed, especially in diabetics receiving RAD001 treatment. Therefore, patients with diabetes should have their

blood glucose monitored carefully and their medications adjusted, as needed, to maintain adequate control of their blood glucose levels.

Outside the particular context of hemorrhagic gastritis in advanced gastrointestinal stromal tumor (GIST) patients treated with RAD001 and imatinib, serious, suspected drug-related hemorrhages have been exceptional. Nevertheless, RAD001 should be considered as predisposing patients to hemorrhage, potentially fatal, should they develop severe drug-related thrombocytopenia. Patients with on-going thrombocytopenic or with a known bleeding diathesis should be subject to careful evaluation and more frequent monitoring. Platelet counts should be monitored. Imatinib (Glivec® / Gleevec™), a 3A4 and Pgp substrate, has been shown to increase the AUC of RAD001 more than 3-fold, most probably the consequence of competitive inhibition.

RAD001 is an immunosuppressant and consequently patients taking the drug are at an increased risk of infection. This may be the case even if the patient has a normal white cell count. Physicians and patients/carers should be aware of this risk, and vigilant for signs and symptoms of infection. Prompt treatment with appropriate anti-infectives should be given as clinically appropriate.

Non-infectious pneumonitis is a known side effect of rapamycin analogues including RAD001. Clinically significant pneumonitis is typically accompanied by non-specific symptoms including dyspnea, nonproductive cough, fatigue, and fever. Diagnosis is generally suspected in individuals receiving mTOR inhibitors who develop these symptoms or in asymptomatic individuals in whom a routine chest CT scan reveals a new ground glass or alveolar infiltrate. The frequency of symptomatic pulmonary toxicity (all grades) was approximately 13% in a phase III study of RAD001 in patients with metastatic renal cell carcinoma ([CRAD001C2240]). Severe (CTC grade 3) pneumonitis occurred in 4% of patients, and an occasional fatality was reported. The lung toxicity was partly or completely reversible in the majority of cases with interventions including drug interruption, discontinuation and the use of corticosteroids. For the most up to date safety information, please refer to the most current edition of the Investigator's Brochure.

Adherence to the recommendations in this protocol (including Table 6-3) should ensure detection of clinically relevant pneumonitis occurring in patients and its appropriate management.

1.3.7 Phase I study of everolimus in pediatric patients with refractory solid tumor

In the phase I Study CRAD001C2413 (IND #70,714) everolimus was administered orally at a daily dose of 2.1, 3, 5 and 6.5 mg/m^2 in cohorts of three to six patients per dose level (Fouladi et al 2007). The MTD for this population was 5 mg/m^2. At the starting dose of 3 mg/m^2 readily reversible grade 3 and 4 dose-limiting toxicities (DLTs) at 3 mg/m^2 included reversible hypokalemia (n = 1, grade 4) and hypophosphatemia (n = 2, grade 3) were observed in platinum pretreated patients, resulting in dose de-escalation to 2.1 mg/ m^2 . The definition of DLT was amended to exclude grade 3 or 4 electrolyte abnormalities that resolved to ≤ grade 2 within 7 days of interrupting treatment, allowing further dosage escalation). Dose de-escalation to 2.1 mg/m^2 led to no further DLTs in three assessable patients. Three more assessable patients were then enrolled at 3 mg/m^2 and 5 mg/m^2, each with no DLTs. At 6.5

mg/m^2, the DLTs included grade 3 events of elevation of ALT (n = 1), mucositis (n = 1), and diarrhea (n = 1). Thus, three more patients were enrolled at 5 mg/m^2, with no observed DLTs, establishing 5 mg/ m^2 as the recommended MTD. No additional grade 4 events were reported. Additional grade 3 events at any time during trial therapy were ALT elevation (n=1, 5 mg/m^2), reduced hemoglobin (n=2, 3mg/m^2), infection (n=3, 3 mg/m^2), leucopenia (n=2, 3 mg/m^2), mucositis (n=1), hypokalemia (n=1, 3 mg/m^2 and 5 mg/m^2), hyponatremia (n=1, 3 mg/m^2), anorexia (n=1, 3mg/m^2), dizziness (n=1, /m^2), hyperglycemia (n=1, 2.1 mg/m^2 and 5 mg/m^2), nausea (n=1, 3 mg/m^2) and pain in the oral cavity (n=1, 5 mg/m^2). The adverse events seen were consistent with the known safety profile of RAD001 in adults (Fouladi et al 2007).

Everolimus pharmacokinetics in children was also found to be comparable to adults. Everolimus was absorbed rapidly, with maximum concentrations achieved as early as 30 minutes after administration. The maximum everolimus whole-blood concentrations and AUC at each dose level were variable, but increased with dose.

1.3.8 RAD001 phase II study in SEGA

Currently, there is an investigator-initiated study single center, open label phase II study (Clinicaltrials.gov Identifier NCT00411619) for patients with SEGA associated with TSC.

This trial is open for patients aged 3 years or older with confirmed diagnosis of TSC and radiological evidence of serial growth of at least one SEGA lesion prior to study enrollment. The starting daily dose of RAD001 is 3.0 mg/m^2, with subsequent titration to attain a target trough level of 5 - 15 ng/ ml. The primary efficacy endpoint of the trial is reduction in SEGA volume.

All patients had MRI scans with volumetric measurements of SEGA at baseline and at 3 and 6 months after start of therapy. The largest SEGA showing serial growth was defined as the primary SEGA, all other SEGA were defined as secondary. The first 6 months were defined as the core phase of the study. Patients with clinical benefit and no unacceptable toxicity in the opinion of the principal investigator were offered an opportunity to continue open label RAD001 in an extension phase where MRI scans were obtained every 6 months. A 24-hour video EEG was performed at baseline and at 24 weeks on all patients with uncontrolled epilepsy (defined as those having at least one seizure per month prior to study enrollment) and seizure frequency is also followed using patient diaries. Neuropsychometric function was assessed using a battery of age appropriate tests.

Twenty-eight patients between the ages of 3 and 34 years with TSC-associated SEGA were enrolled between 07-Jan-2007 and 18-Dec-2008.

Responses at 3 and 6 months based on primary tumor volume are summarized in Table 1-4 while those based on total tumor volume (sum of the volume of primary and secondary lesions) are summarized in Table 1-5.

Table 1-4 Primary tumor volume responses

	Everolimus					
	**Baseline n=28**	**3 months n=27**	**6 months n=23**	**12 months n=16**	**18 months n=5**	**24 months n=2**
**Mean SEGA volume (cm^3)**	2.25	1.41	1.24	1.62	1.31	1.25
**Range**	0.35-7.10	0.18-4.00	0.19-3.40	0.37-6.30	0.49-2.60	1.10-1.40
**Mean percentage reduction from baseline**		34	40	41	47	55
**Range**		0-73	3-75	5-66	27-63	52-59
**Reduction in tumor volume (n [%]) from baseline[1]**						
**≥ 50%**		8(30)	9(39)	5(31)	2(40)	2(100)
**≥ 30%**		15(56)	17(74)	12(75)	4(80)	2(100)
**> 0%**		27(100)	23(100)	16(100)	5(100)	2(100)
**Tumor growth relative to baseline**		0	0	0	0	0

[1] A ≥ 30% reduction in tumor volume was considered to reflect a clinically meaningful reduction

Table 1-5 Total tumor volume responses

	Everolimus					
	**Baseline n=28**	**3 months n=27**	**6 months n=23**	**12 months n=16**	**18 months n=5**	**24 months n=2**
Mean SEGA volume (cm^3)	2.63	1.54	1.36	1.80	1.43	1.35
Range	0.35-7.65	0.18-4.08	0.19-3.48	0.51-6.30	0.49-2.80	1.10-1.60
Mean percentage reduction from baseline		35	40	40	50	56
Range		4-78	3-78	5-72	38-63	52-60
Reduction in tumor volume (n [%]) from baseline[1]						
≥ 50%		9(33)	8(35)	5(31)	3(60)	2(100)
≥ 30%		16(59)	17(74)	11(69)	5(100)	2(100)
> 0%		27(100)	23(100)	16(100)	5(100)	2(100)
Tumor growth from baseline		0	0	0	0	0

[1] A ≥ 30% reduction in tumor volume was considered to reflect a clinically meaningful reduction

Everolimus is clearly associated with a clinically meaningful reduction in SEGA volume, the primary efficacy endpoint of the study.

One patient was taken off study 02-Jun-2009, after 18 months of therapy due to treatment success as defined by the protocol (i.e., > 75% reduction in SEGA volume). As of 16-Jun-2009, 25 patients were ongoing in this trial, all of whom had entered the extension phase. The maximum duration of treatment to this date is 29 months.

No patient developed new lesions, worsening hydrocephalus, or worsening signs or symptoms of increased intracranial pressure, and none required surgical resection or other therapy for SEGA.

A total of 18 patients met the criteria of uncontrolled epilepsy and were evaluated with 24-hour video EEG monitoring at baseline and at 6 months; to date, data are available for 12 of these patients. Results of seizure frequency analysis suggest that everolimus was associated with a clinically relevant reduction in the frequency of both subclinical seizures and interictal epileptiform discharge (IED).

Interpretation of the everolimus effect on clinical seizures was compromised by the fact that only 2 of the 12 patients had clinical seizures on the baseline EEG. Both of these patients had a lower frequency of clinical partial seizures at 6 months; however, one also had generalized seizures at baseline which had increased in frequency at the 6-month EEG (at this time it is not known how many of these generalized seizures were clinical or subclinical). Note: none of the 10 patients without clinical seizures at the baseline EEG had clinical seizures at the 6-month EEG.

In total, 187 adverse events (AEs) were reported. The most common AEs by verbatim term irrespective of relationship to treatment, in descending order of frequency, were oral mucositis, upper respiratory infection, sinusitis, otitis media, fever, gastric infection, acneiform rash, and skin infection. These events are consistent with the known safety profile of everolimus, derived from earlier studies, and primarily represent grade 1 (mild) or grade 2 (moderate) events.

Fifty-five AEs were considered to be probably or definitely attributed to the study drug by investigator assessment. The most common drug-related AEs were oral mucositis, although this tended to be mild (maximum severity grade 2), urinary tract infection, and increased serum cholesterol.

One serious AE of viral bronchitis was reported. The patient recovered and study drug was re-started. The patient was still being treated as of May-2009.

To date, 2 patients have discontinued treatment with everolimus due to adverse events of hyperkinesis (lack of compliance with antiepileptics) and infection.

These results provide strong evidence that everolimus produces clinically meaningful reductions in SEGA volume in patients with TSC- associated SEGA and suggest that everolimus was associated with a clinically relevant reduction in subclinical seizure and IED frequency. No patient developed new lesions, worsening hydrocephalus, or worsening signs or symptoms of increased intracranial pressure, and none required surgical resection or other therapy for SEGA.

The high response rates, low incidence of AEs, and absence of major safety issues suggest a favorable benefit-risk assessment, thus helping to establish everolimus as a safe and effective pharmacotherapeutic agent for this patient population, for whom there is a significant unmet medical need.

For additional efficacy and safety information, please refer to the most recent edition of the Investigator's Brochure as well as the Afinitor prescribing information revised October 2010.

1.4 History of Amendments

1.4.1 Amendment 1

- Changed the definition of SEGA progression as follows:
  - Either, an increase from nadir of 25% or more in SEGA volume to a value greater than baseline SEGA volume (where SEGA volume is the sum of the volumes of all target SEGA lesions identified at baseline and where nadir is the lowest SEGA volume achieved by the patient previously in the trial (including baseline)) , or
  - the unequivocal worsening of non-target SEGA lesions, or
  - the appearance of a new SEGA lesions $\geq$ 1cm in longest diameter, or
  - new or worsening hydrocephalus, (defined by central radiological assessment of ventricular configuration changes, ventricular cap signs (periventricular edema) and qualitative assessment of cerebrospinal fluid (CSF) flow dynamics).
- Changed the requirement of a confirmatory response scan from at least 4 weeks after the first assessment of response to approximately 12 weeks after the first assessment of response.
- Changed the confirmation of skin lesion response from at least 4 weeks after the first response assessment to approximately 12 weeks after the first response assessment.
- Removed pulmonary function tests (PFT) at baseline and at every study visit and replaced with PFTs to be performed as clinically indicated. Removed the requirement for chest CT at baseline for patients who are unable to perform PFTs. Changed pulmonary exclusion criteria language to reflect that PFTs are not mandated.
- Removed prior brain surgery as an exclusion criterion.
- Allow assessment of angiomyolipomas to be carried out by CT scan as well as by MRI.
- State that informed consent should be obtained according to local guidelines.
- Required everolimus levels to be taken after dose increases to a new level, but not after dose decreases, nor after re-escalation to a previously used level. Require everolimus levels to be taken when relevant enzyme inducers are decreased or enzyme inhibitors are increased.
- Increased from three to six weeks the amount of time off study drug (e.g. for an adverse event or surgery) which is permitted before a patient is discontinued from the study.
- Increased the screening period from 14 to 21 days. Permit screening bloods carried out within 14 days of treatment day 1 to be used for baseline values.
- Require a urine pregnancy screen at treatment day 1.
- Updated Table 6-1, Table 6-3 and added Table 6-2, to update the current guidelines for RAD001 dosing adjustments and guidelines for managing toxicities.
- Changed standard enzyme inducer and inhibitor language to reflect the fact that the majority of the study population will be taking enzyme inducers, and that the study uses a target trough level with routine therapeutic drug monitoring.
- Clarify that if a calculated dose decrease does not result in an actual decrease in the number of tablets taken due to rounding, the dose should be decreased by 1 mg/day.

- Permit patients tolerating the drug with no adverse events to have a telephonic visit and local lab draws for the 6 week visit. Permit the same for any ad hoc trough levels.
- Removed Post-Text Supplement-1
- Implemented the use of the Schwartz formula to evaluate renal function in patients under the age of 18.

1.4.2 Amendment 2

- The CT/MRI kidney scanning option was updated to clarify that the same modality should be used throughout the study for each individual patient.
- Patients who meet a prespecified criteria at baseline will now be screened for Hepatitis B and C at baseline, using the following tests: HBV-DNA, HBsAg, HBs Ab, HBc Ab, HCV-RNA-PCR.
- Hepatitis B and C management guidelines have been added for patients who are receiving study drug at the time of amendment 2 implementation.
- Harmonized the visit window language for all visits.
- Clarified that the study drug will be provided free of charge for as long as the medication is not commercialized and added to the list of reimbursed medications for TSC.
- Removed use of estrogen containing medications as an exclusion criterion and from the list of restricted medications.
- Updated the language related to management of hyperlipidemia and hyperglycemia.
- Updated the study drug dosing instructions to be consistent across the RAD001 program. The language now states that the medication can be taken following a meal.
- Updated the restricted medication list to state that moderate and strong inhibitors of CYP3A4 must be avoided. In addition, strong inducers of CYP3A4 other than antiepileptics must be avoided. Inhibitors of PgP should also be avoided during the study.
- Added a table of PgP substrates, inhibitors and inducers.
- Added follow-up urine pregnancy tests to be conducted every 12 weeks after the start of study drug.
- Added provision for an End of Treatment scan if the patient discontinued for reasons other than progression and enough time has passed since their most recent scan.
- Clarified that while confirmation of response should be done approximately 12 weeks after initial response, it should be no sooner than 8 weeks after.
- Clarified the instructions for the permitted local laboratory collections for patients in the United States for whom travel to the clinic is difficult.
- Clarified that a patient must return to the clinic for PK collections 1-2 weeks after a dose increase to a new level or an increase in a CYP3A4 or PgP inhibitor or decrease in a CYP3A4 or PgP inducer
- Updated the angiomyolipoma progression definition to align with the SEGA progression definition that was updated as part of the previous amendment.

- Added exclusion criterion that any severe and/or uncontrolled medical conditions which could cause unacceptable safety risks or compromise compliance with the protocol and provided examples.
- Modified the target RAD001 concentration range from 10-15 ng/mL to 5-15 ng/mL
- Increased the maximum possible dose to achieve the target range of 5-15 ng/mL by adding an additional 2 dose level increments; 10.67 mg/m^2 and 14.22 mg/m^2
- Removed the requirement for pregnancy outcome follow-up for pregnant partners of male participants.
- Added endocrine blood testing (testosterone, LH, FSH and Estradiol) at screening and subsequent timepoints according to the patient's age and gender
- Added Tanner staging to assess sexual maturation at screening and subsequent timepoints according to the patient's age
- Added the requirement for baseline scans to be read centrally prior to randomization, for those patients whose SEGA lesions are less than 1.5 cm at baseline.

2 Study rationale/purpose

The purpose of this study is to assess the efficacy and safety of RAD001 in treating subependymal giant cell astrocytomas (SEGAs) associated with tuberous sclerosis complex (TSC). This study will evaluate the antitumor activity and clinical benefits of RAD001 given at a starting dose of 4.5 mg/m^2 per day, with up-titration or down-titration possible to achieve the intended RAD001 concentration of 5-15 ng/ml.

mTOR pathway upregulation has been observed in lesions derived from TSC patients (Astrinidis and Henske 2005; Kwiatkowski and Manning 2005), and TSC1 or TSC2 defective experimental animal models exist which recapitulate the pathology, behavioral and neurological aspects of the tuberous sclerosis disease (Onda et al 1999; Kwiatkowski and Manning 2005; Uhlmann et al 2002; Kenerson et al 2005) and are sensitive to mTOR inhibition (Kenerson et al 2005; Astrinidis and Henske 2005; Kwiatkowski and Manning 2005). A dramatic improvement in survival has been observed in a mouse brain model of TSC (genotype: $Tsc1^{cc}$ $syn\text{-}cre^{+}$), with a highly statistically significant improvement in survival ($p < 0.0001$) associated with RAD001 treatment (Figure 2-1). The investigator also noted an improvement in behavior, weight gain and neurological phenotype. Assessment of brain pathology is currently ongoing (Data kindly provided by D Kwiatkowski May 2007).

Figure 2-1 Survival in a mouse brain model of TSC

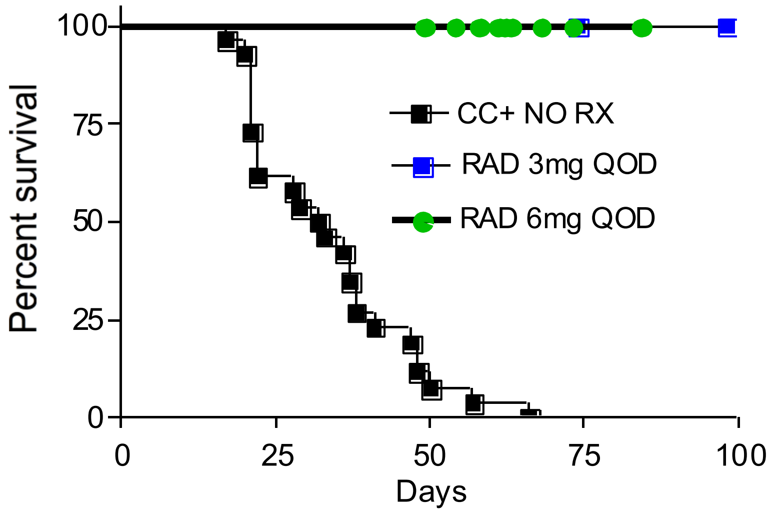

Also, both estrogen and vascular endothelial growth factor (VEGF) signaling have been implicated in the pathogenesis and vascularization of TSC lesions (Astrinidis and Henske 2005; Kwiatkowski and Manning 2005). In this respect, RAD001 has been shown to inhibit both estrogen and VEGF-dependent signaling events (Boulay et al 2005; O'Reilly et al 2005).

Finally, encouraging preliminary data from the investigator-initiated trial are discussed in Section 1.3.4.1

Taking all these data into account, there is a strong rational for using mTOR inhibitors such as RAD001 for the treatment of patients with TSC-associated SEGAs.

3 Objectives

3.1 Primary objectives

To compare SEGA response rate on RAD001 versus placebo in patients with TSC-associated SEGA.

3.2 Secondary objectives

To compare RAD001 versus placebo with respect to:

a Change from baseline in frequency of epileptiform events.

b Time to SEGA progression.

c Skin lesion response rate.

d Change from baseline in plasma angiogenic molecules, e.g., VEGF, basic FGF, PLGF, soluble VEGF receptor1, and soluble VEGF receptor2.

e Renal function assessed using calculated creatinine clearance.

f Safety as assessed by the NCI Common Toxicity Criteria, version 3.0.

In RAD001 treatment arm to:

1. Characterize the pharmacokinetics of RAD001 in this patient population, specifically in terms of exposure.
2. Describe the time to SEGA response, the duration of SEGA response, and the duration of skin lesion response.

4 Study design

This is a prospective, double-blind, randomized, parallel group, placebo-controlled, multi-center phase III study evaluating treatment with RAD001 versus placebo in 99 patients with TSC-associated SEGA.

There are four separate phases in this study: pre-treatment (screening/baseline), blinded treatment, extension and follow-up. Each of these phases is described in detail below.

4.1 Pre-treatment phase (Screening/Baseline)

At screening/baseline, the investigator or his/her designee will assign a unique number (refer to Section 6.3) to patients being considered for the study. The patient must provide a signed Informed Consent Form prior to any study screening evaluations being performed. Once the patient provides a **signed informed consent form** and **eligibility is confirmed (all inclusion/exclusion criteria have been verified)** the investigator or his/her designee can register the patient using an Interactive Web Response System (IWRS), a central patient screening/randomization system. Refer to Section 6.3, Section 6.1.3.1 and Section 6.4 for complete details.

During the 28-day screening period, all patients will have MRI of the brain and CT/MRI of the kidney performed for identification of SEGA lesions and angiomyolipomata. For each patient, the same imaging modality should be used throughout the trial. A pre-baseline MRI

of the brain, conducted prior to the baseline MRI, must be obtained and used by the investigator and the local radiologist to determine whether the patient satisfies the radiological criteria for entry into the trial. These include assessing any increase in SEGA volume, appearance of a new SEGA lesion or increase in the volume of the lateral ventricles (for complete definition, see point 4 of the inclusion criteria, Section 5.1). Each participating center should assess these radiological criteria according to their own clinical practice using the best methods available. It is known that not all sites will have the technical capability to precisely measure SEGA volume and ventricular volume, and also that some pre-baseline MRIs may not be in digital format. In such cases qualitative assessments should be made.

If upon local review of the baseline brain scan, the longest diameter of the largest SEGA lesion is less than 1.5 cm (between 1.0 cm and 1.4 cm), the baseline scan must immediately be sent to the central reviewer prior to the patient being randomized. The central reviewer will confirm whether the patient's SEGA meets the minimum longest diameter criterion of 1.0 cm and provide feedback to the investigator within 1 week of receiving the scan. The investigator can then proceed with randomization provided all eligibility criteria have been confirmed and the central reader agrees that the patient has a SEGA lesion of at least 1.0 cm in longest diameter. If, according to central review, the patient does not have a SEGA lesion of at least 1.0 cm, the patient will be considered a screen failure and should not be randomized. The serial growth (inclusion criterion # 4) criteria will be reviewed and confirmed locally. Therefore, the prebaseline scan will not need to be sent to the central reader until after randomization.

The decision to randomize the patient will be made based on the judgment of the investigator and local radiologist in coordination with the central reader (for patients with SEGA's between 1.0 cm and 1.4 cm according to local review). As with the local radiological review, if the pre-baseline MRI was only available on film (non-digital format), then the central reviewer should make a qualitative assessment of any increase in SEGA volume, appearance of a new SEGA lesion or increase in volume of the lateral ventricles.

There is no plan to collect radiological measurements made by the local radiologist, partly because not all sites will have the same methods of measurement, but also because all data analysis will be based on the measurements obtained from the central radiological review.

Hepatitis Screening

Prior to randomization, the following three categories of patients should be tested for hepatitis B viral load and serologic markers, that is, HBV-DNA, HBsAg, HBs Ab, and HBc Ab:

1. All patients who currently live in (or have lived in) Asia, Africa, Central and South America, Eastern Europe, Spain, Portugal, and Greece. [http://wwwnc.cdc.gov/travel/yellowbook/2010/chapter-2/hepatitis-b.aspx#849]
2. Patients with any of the following risk factors:
   - known or suspected past hepatitis B infection,
   - blood transfusion(s) prior to 1990,
   - current or prior IV drug users,
   - current or prior dialysis,

- household contact with hepatitis B infected patient(s),
- current or prior high-risk sexual activity,
- body piercing or tattoos,
- mother known to have hepatitis B
- history suggestive of hepatitis B infection, e.g., dark urine, jaundice, right upper quadrant pain.

3. Additional patients at the discretion of the investigator

If a patient tests positive, they will be considered ineligible for the study according to Exclusion Criterion 6. Please note that patients who test negative for HBV-DNA, HBsAg, and HBc Ab but positive for HBs Ab with prior history of vaccination against Hepatitis B will be eligible. The fact that the patient had been vaccinated should be entered into the patient's Medical History CRF.

Screening for hepatitis C

Patients with any of the following risk factors for hepatitis C should be tested using quantitative RNA-PCR:

- known or suspected past hepatitis C infection (including patients with past interferon 'curative' treatment),
- blood transfusions prior to 1990,
- current or prior IV drug users,
- current or prior dialysis,
- household contact of hepatitis C infected patient(s),
- current or prior high-risk sexual activity,
- body piercing or tattoos

At the discretion of the investigator, additional patients may also be tested for hepatitis C.

If a patient tests positive, they will be considered ineligible for the study according to Exclusion Criterion 6.

For patients who have already been randomized and received study drug prior to the approval of amendment 2, the same screening process should be followed at the patient's next visit. If the patient tests positive for Hepatitis B, the investigator should follow the guidelines according to Table 4-1 and Table 4-2. Please refer to Table 7-1 and Table 7-2 for HCV RNA-PCR monitoring schedule for those patients with positive HCV RNA-PCR baseline tests who do not meet the HCV flare criteria outlined in Table 4-3. If the patient tests positive for hepatitis C, and the criteria for HCV flare according to Table 4-3 are observed, trial therapy should be discontinued and further treatment is up to the investigator's discretion.

Table 4-1 Action to be taken for positive baseline hepatitis B results for patients that are active prior to Amendment 2 approval

Test	Result	Result	Result	Result	Result
HBV-DNA	+	+ or -	-	-	-
HBsAg	+ or -	+	-	-	-
HBs Ab	+ or -	+ or -	+<br>and no prior HBV vaccination	+ or -	-<br>or + with prior HBV vaccination
HBc Ab	+ or -	+ or -	+ or -	+	-
Recommendation	Prophylaxis treatment should be started and study drug dose interruption is recommended for 14 days<br>Monitor HBV-DNA approximately every 4-8 weeks		No prophylaxis<br>Monitor HBV-DNA approximately every 3-4 weeks		No specific action

Table 4-2 Guidelines for management of hepatitis B for patients that are active prior to Amendment 2 approval

**HBV reactivation (with or without clinical signs and symptoms)***	
For patients with baseline results:<br>Positive HBV-DNA<br>**OR**<br>positive HBsAg<br>-------------------------------------<br>reactivation is defined as:<br>**[**Increase of 1 log in HBV-DNA relative to baseline HBV-DNA value **OR** new appearance of measurable HBV-DNA**]**	**Treat**: Start a second antiviral<br>AND<br>Interrupt study drug administration until resolution:<br>• ≤ baseline HBV-DNA levels<br>**If resolution occurs within ≤ 28 days** study drug should be re-started at one dose lower, if available. (see Table 6-4 - Dose levels for dose adjustments) If the patient is already receiving the lowest dose of study drug according to the protocol, the patient should restart at the same dose after resolution. Both antiviral therapies should continue at least 4 weeks after last dose of study drug.<br>**If resolution occurs > 28 days** Patients should discontinue study drug but continue both antiviral therapies at least 4 weeks after last dose of study drug.
For patients with baseline results:<br>Negative HBV-DNA **and** HBsAg<br>**AND**<br>**[**Positive HBs Ab (with no prior history of vaccination against HBV), **OR** positive HBc Ab**]**<br>------------------------------------<br>reactivation is defined as:<br>New appearance of measurable HBV-DNA	**Treat** : Start first antiviral medication<br>AND<br>**Interrupt** study drug administration until resolution:<br>• ≤ undetectable (negative) levels<br>**If resolution occurs within ≤ 28 days** study drug should be re-started at one dose lower, if available. (see Table 6-4 - Dose levels for dose adjustments) If the patient is already receiving the lowest dose of study drug according to the protocol, the patient should restart at the same dose after resolution. Antiviral therapy should continue at least 4 weeks after last dose of study drug.<br>**If resolution occurs > 28 days** Patients should discontinue study drug but continue antiviral therapy at least 4 weeks after last dose of study drug.

**HBV reactivation (with or without clinical signs and symptoms)***
* All reactivations of hepatitis B are to be recorded as grade 3 (CTCAE v 3.0 Metabolic Laboratory/Other: Viral Re-activation), unless considered life threatening by the investigator; in which case they should be recorded as grade 4 (CTCAE v 3.0 Metabolic Laboratory/Other: Viral Re-activation). Date of viral reactivation is the date on which the rise or reappearance of HBV- DNA was recorded .

Table 4-3 Guidelines for management of hepatitis C for patients that are active prior to Amendment 2 approval

**HCV flare***	
**For patients with baseline results:**<br>**Detectable HCV-RNA**<br><br>**HCV flare is defined as:**<br>> 2 $\log_{10}$ IU/mL increase in HCV-RNA<br>**AND**<br>**ALT elevation** > **5 X ULN** or 3 x baseline level, whichever is higher.	**Discontinue study drug**
**For patients with baseline results:**<br>**Knowledge of past hepatitis C infection with no detectable HCV-RNA**<br><br>**HCV flare is defined as:**<br>**New appearance of detectable HCV-RNA**<br>**AND**<br>ALT elevation > 5 x ULN or 3 x baseline level, whichever is higher.	**Discontinue study drug**
* All flares of hepatitis C are to be recorded as grade 3 (CTCAE v 3.0 Metabolic Laboratory/Other: Viral Flare), unless considered life threatening by the investigator; in which case they should be recorded as grade 4 (CTCAE v 3.0 Metabolic Laboratory/Other: Viral Flare). Date of viral flare is the date on which both the clinical criteria described above were met. (e.g., for a patient whose HCV-RNA increased by 2 logs on 01 JAN 2011 and whose ALT reached > 5 x ULN on 22 JAN 2011, the date of viral flare is 22 JAN 2011.	

Other screening assessments include blood sampling (including endocrine evaluations), urinalysis, performance status (WHO or Lansky, depending on the age of the patient), ECG, biochemical and biomarker evaluations (unless local or national regulations do not permit), and neuropsychological assessments. For all patients, all available prebaseline height and weight data should be collected in order to adequately represent the patient's rate of growth prior to starting the study. Height and weight will continue to be assessed throughout the study according to the schedule of assessments.

If safety laboratory collections are collected more than 14 days prior to Treatment Day 1, they will need to be repeated prior to the patient's first dose of study drug. The laboratory collections that must be repeated are: hematology, endocrine, biochemistry and lipid panel, coagulation, urinalysis and pregnancy testing.

Once the patient is confirmed as being eligible for study participation, a 24-hour video EEG will be conducted, and the results sent for independent central review. In addition, patients being treated with antiepileptics at baseline will complete a seizure severity questionnaire (SSQ) at screening, whenever possible. Patients with skin lesions at baseline will have digital photos of these lesions taken during screening. Screening evaluations will also include demography, relevant medical history/current medical conditions, a physical examination (including, height (using a stadiometer) and weight, a neurological examination and Tanner Staging at the specified timepoints), evaluation for growth and development milestones (age at thelarche (females), age at adrenarche (males) and date of menarche (females)), vital signs and other additional study entry evaluations. All screening/baseline evaluations should be completed in the 28 days prior to Treatment Day 1. A complete list of screening evaluations is provided in the schedule of evaluations (Table 7-1). All of the above assessments/procedures must be conducted prior to randomization. Once the patient is randomized, the baseline kidney and brain MRIs should be sent as soon as possible by the study site to the Independent Central Radiology Reviewer.

4.2 Blinded treatment phase

Patients who meet the study eligibility criteria will be randomized to receive RAD001 or matching placebo. The randomization ratio is 2:1, with two patients being randomly assigned to RAD001 for every one patient randomly assigned to matching placebo. Randomization will be stratified by the use of enzyme-inducing anti-epileptic drugs (EIAED: yes versus no). The following drugs qualify as EIAED: phenytoin (Dylantin®, Dilantin Kapseals®, Dilantin Infatabs®, Eptoin®, Epanutin®, Diphenin®, Dipheninum®, Phenytek®), mephenytoin (Mesantoin®), carbamazepine (Tegretol®, Biston®, Calepsin®, Carbatrol®, Epitol®, Equetro®, Finlepsin®, Sirtal®, Stazepine®, Telesmin®, Teril®, Timonil®, Trimonil®, Epimaz®, and Degranol®), phenobarbital (Luminal®), pentobarbital (Nembutal®), primidone (Mysoline®), oxcarbazepine (Trileptal®). Randomization and study medication management will be done through IWRS. **Patients should start treatment as soon as possible after randomization and no more than 7 days, at the latest, after randomization.**

This study does not have a fixed treatment duration. Patients will have their first daily dose of RAD001 or matching placebo at Visit 2 (Treatment Day 1) and will continue on treatment until SEGA progression (see Section 7.5.2), unacceptable toxicity, withdrawal of consent or investigator decision to discontinue the patient from study treatment.

Each patient will start treatment at a dose of 4.5 mg/m^2/day. Dose adjustments will be permitted based on safety findings and blood trough measurements, as described in Section 6.7.2.

Safety evaluations are routinely performed at each visit. Patients must be in a fasting state (at least 12 hours) at the time of blood sampling for all laboratory evaluations including the lipid profile. Hematology and biochemistry assessments will be performed at each visit including study discontinuation. Note that hematology and biochemistry labs are performed at screening

and do not need to be repeated on Treatment Day 1. Endocrine testing (Testosterone, FSH, LH and Estradiol (for females) will be completed annually until the patient's 10th birthday and then every 12 weeks thereafter. For patients who are 10 years old or older at the time of Amendment, endocrine blood sampling will be completed every 12 weeks.

In addition, Tanner staging will be completed annually as part of the physical exam. Attainment of growth and development milestones (age at thelarche (females), age at adrenarche (males) and date of menarche (females)) should also be evaluated. Height and weight will be assessed during the physical exam at the timepoints indicated in Table 7-1 and Table 7-2. All blood samples obtained at each visit will be sent to a Central Laboratory for analysis. If laboratory results are requested on an urgent basis, the attending physician will use the local laboratory results for treatment decisions. Complete details regarding all study required safety assessments are provided in Section 7.6

During this phase of the study, all patients will have an MRI of the brain at 12, 24 and 48 weeks after start of study treatment, and annually thereafter until SEGA progression. An MRI of the brain should be repeated at the End of Treatment (EOT) visit if the patient has discontinued for any reason other than SEGA progression, and if it has been more than 8 weeks since their most recent brain MRI during the first year of treatment, or more than 6 months since their most recent brain MRI thereafter.

In addition to the MRI of the brain, patients with an angiomyolipoma having a longest diameter ≥ 1.0 cm documented at screening/baseline will also have a CT/MRI of the kidneys at 12, 24 and 48 weeks after start of study treatment, and annually thereafter until study end. For each patient, the same imaging modality should be used throughout the trial.

All MRIs of the brain and CT/MRIs of the kidneys obtained during the blinded phase will be sent to the central radiologist within 2 days of the scan for an independent centralized radiology review. The central radiologist will determine the SEGA volume and verify whether the radiological criteria for SEGA response have been met (see Section 7.5.2). Since not all study centers will have the technical capability to measure SEGA volume and ventricular volume, there is no plan to collect data from the local radiologist at each site. The central review of an MRI will be available no more than 3 weeks after its receipt by the central reviewer, and once available the results will be sent immediately to the study site. **If the patient is assessed as having a response, a repeat radiological confirmation should be performed approximately 12 weeks from the initial observation** (and no sooner than 8 weeks). For patients who respond at 12 weeks of treatment, the routine 24 week scan is sufficient to confirm response. Clinical suspicion of SEGA progression at any time requires a physical examination and radiological confirmation should be performed promptly rather than waiting for the next scheduled radiological assessment. If SEGA progression was **unequivocal** according to the local radiologist, then the investigator should contact the Novartis Clinical Trial Head (CTH) or designee for review, and without the need of waiting up to 3 weeks for confirmation from central radiology review.

Other tests that will be conducted routinely during the blinded treatment phase include laboratory tests for safety, physical examinations (including a neurological assessment), vital signs, performance status (WHO or Lansky, depending on age of patient), ECG, and neuropsychological assessments. In addition to these tests, patients with skin lesions will have digital photos of their skin lesions taken every 12 weeks. A complete list of evaluations

can be found in Table 7-1 and Table 7-2. If unforeseen circumstances (i.e., unexpected personal reasons) prevent the patient from complying with the established visit schedule, the site can re-schedule the visit (within the prescribed visit window as noted in Table 7-1 and Table 7-2). The reason(s) for any visit or treatment delays will be documented on the Comments CRF for the appropriate visit.

Pharmacokinetic assessments will be performed according to the schedules provided in Table 7-1 and Table 7-2 Trough pharmacokinetic assessments (pre-dose, C_{min}) are planned for all patients at every visit starting with Visit 3 and until discontinuation from study drug. Two weeks after any dose increase to a new level, or any decrease in an enzyme-inducing drug, or any increase in an enzyme-inhibiting drug a trough and an additional pharmacokinetic assessment will be collected 2.0 hours (± 30 mins, C_{2h}) after study drug administration.

Biomarker research studies will be performed according to the schedule provided in Table 7-1 All patients (unless local or national regulations do not permit) will have 3 mL of blood collected at screening/baseline, and at 4, 12, 24, 36, and 48 weeks, and at end of treatment. On-treatment samples will be compared to baseline samples for RAD001 effects on plasma angiogenic molecules VEGF, basic FGF, PLGF, soluble VEGF receptor1, and soluble VEGF receptor2.

All patients will donate 3 mL of whole blood at screening/baseline for genetic mutational analyses of TSC1 and TSC2 genes. No tumor material will be investigated as part of this protocol.

All patients taking antiepileptic medications or who completed the questionnaire at baseline will complete the SSQ at 24 weeks or at end of treatment if the patient discontinues before 24 weeks.

Patients who are ongoing at the time Amendment 4 is approved should have the endocrine blood tests and Tanner Staging at their next scheduled visit (if the assessment had not previously been done- considered baseline) and then annually.

4.3 Open-label treatment phase

If SEGA progression (as defined in Section 7.5.2) is documented by central radiology review during the blinded treatment phase, then the treating physician may proceed to unblind the patient. Central radiology review of an MRI will be completed no later than 3 weeks after its receipt by the central reviewer, and so the unblinding may not be able to take place until up to 3 weeks after the progression was actually observed. However, if progression was unequivocal according to the local radiologist, then the investigator should contact the Novartis Clinical Trial Head (CTH) or designee to proceed with unblinding without waiting for confirmation from central radiology review.

Following unblinding, patients who had been receiving placebo may be offered open-label treatment with RAD001 if the treating physician believes the patient could benefit from this therapy.

For the open-label phase of the study, the most recent MRI of the brain and video EEG from the blinded phase of the study will be used as the baseline if completed within 12 weeks of starting open-label treatment, and further MRIs of the brain will be conducted at 12, 24 and 48 weeks after the start of open-label RAD001, and annually thereafter, until SEGA progression,

unacceptable toxicity or discontinuation for any other reason. An additional video EEG will be collected 6 months after the patient starts open-label treatment. An additional MRI of the brain will be obtained at End of Treatment in the open-label RAD001 phase, provided discontinuation was not for SEGA progression and if it had been more than 8 weeks since the most recent brain MRI during the first year of open-label treatment, or more than 6 months since the most recent brain MRI thereafter. If CT/MRIs of the kidneys had been conducted in the blinded phase of the study, then CT/MRIs of the kidneys will be performed 12, 24 and 48 weeks after start of open-label RAD001, and annually thereafter until study end and the latest CT/MRI of the kidney from the blinded treatment phase will be used as the baseline. For each patient, the same imaging modality should be used throughout the trial. All imaging scans of the brain and kidneys obtained in the open-label phase of the trial will be sent in for central radiological review within 2 days of the scan. Patients receiving open-label treatment with RAD001 will continue having safety and efficacy assessments as in the blinded portion of the trial, as described in the Visit Evaluation Schedule provided in Table 7-2.

Open-label treatment with RAD001 will begin at 4.5 mg/m^2 with up-titration or down-titration possible to achieve the intended RAD001 blood concentration of 5-15 ng/ml. During the open-label phase, treatment will continue until the patient again presents with SEGA progression (second occurrence) which is either radiologically documented or a SEGA-related surgical intervention. At this point, patients will be discontinued from the study and will enter the follow-up phase.

The investigator or his/her designee will **not** disclose patient unblinding information to the central radiology reviewers. Due to the unblinding of a subset of patients at SEGA progression, members of the Novartis clinical trial team, including team members involved in data verification may become unblinded to individual patients' treatment during the conduct of the trial. The blinding of the central radiology review, the basis for the primary analysis of the primary endpoint of the trial, will be maintained despite the planned unblinding to investigators of individual patients.

4.4 Follow-up phase

All patients will have a follow-up visit scheduled 28 days after the last dose of study treatment to assess AEs and SAEs that may have occurred after discontinuation from the study treatment. Beyond these 28 days, any SAEs that are suspected to be related to the study drug and occur within the next 8 weeks (56 days) will also be collected. Medications/therapies given to the patient during the 12 weeks after study treatment discontinuation must be recorded on the CRFs designated to record therapies since discontinuation of the study treatment, unless the patient begins a non-study anti-SEGA therapy, at which time collection of SAEs, AEs and concomitant medications will cease.

Patients without SEGA progression at the time of discontinuation of study treatment will be followed with MRI tumor assessments annually until eventual SEGA progression, the start of any non-study systemic anti-SEGA therapy, withdrawal of consent, or until end of the study, whichever occurs first. During this follow-up period, the site will continue to send MRI scans for central review, and use of non-study systemic anti-SEGA therapies will be recorded.

4.5 Extension phase

There are two distinct parts of this trial, the core and extension phases. The core phase is from the start of the trial up to the time when the last randomized patient has been treated with RAD001 or placebo for 6 months. After the cutoff date, entry into the core phase will be closed and the data will be source data verified and retrieved for the final primary analysis of safety and efficacy. Patients should continue on the same dose and regimen of blinded study drug or open-label RAD001 that they were receiving in the core phase while this primary data analysis is being performed.

If this final data analysis fails to show superiority of RAD001 over placebo, all patients will be discontinued from the study and will have end of study treatment evaluations performed as indicated in Table 7-1 and Table 7-2.

If the final trial results favor RAD001, then an extension phase will be launched. All patients still receiving study treatment at this time, as well as those being followed for post-treatment evaluation, will be given the option of starting open-label RAD001. The extension phase will run until 4 years after the last patient was randomized, ensuring patient follow-up of 4-5 years (assuming patient accrual over a period of approximately 12 months).

Those patients entering the extension phase, who had previously only been receiving placebo, will have scheduled assessments beginning at baseline according to Table 7-2 and will begin their treatment at 4.5 mg/m^2 with up-titration or down-titration possible to achieve the intended RAD001 blood concentration of 5-15 ng/ml. Patients that had been on active RAD001 in the blinded phase prior to beginning the extension phase will simply continue their sequence of assessments according to Table 7-1 or Table 7-2 and continue the same dose they were receiving in the blinded phase. Accordingly, patients that had been on active RAD001 in the Open-label phase prior to beginning the extension phase will simply continue their sequence of assessments according to Table 7-2. Patients in the extension phase will continue to have the same safety, efficacy and pharmacokinetics assessments as in the core phase until SEGA progression, with the exception of biomarker assessments which will not be performed in the extension phase.

Patients who are active in the extension phase at the time of Amendment 5 approval and consent to participate will be asked to have additional PK blood samples collected at any ONE of their regularly scheduled visit days.

Additional PK samples (2 mL/sample for patients 10 years and older and 1 mL/sample for younger patients) will be collected at steady state condition at predose and at 0.5, 1, 2, 5 and 24 hours after the patient's dose on the day of a regularly scheduled study visit. The originally planned pre-dose (and the 2-hour post-dose sample, if required) will not be collected on this visit day.

RAD001 will be provided free of charge by Novartis during the extension phase and until such time when the patient has to stop treatment with RAD001 because of adverse event(s), abnormal laboratory value(s), SEGA progression, patient's condition no longer requires RAD001 therapy, withdrawal of consent, lost to follow-up, death, Novartis discontinues development of RAD001 for this indication, whichever comes first. For all patients receiving RAD001 at the end of the extension phase, RAD001 will continue to be provided free of

charge for as long as the medication is not commercialized and added to the list of reimbursed medications for patients with TSC.

Figure 4-1 Study Flowchart

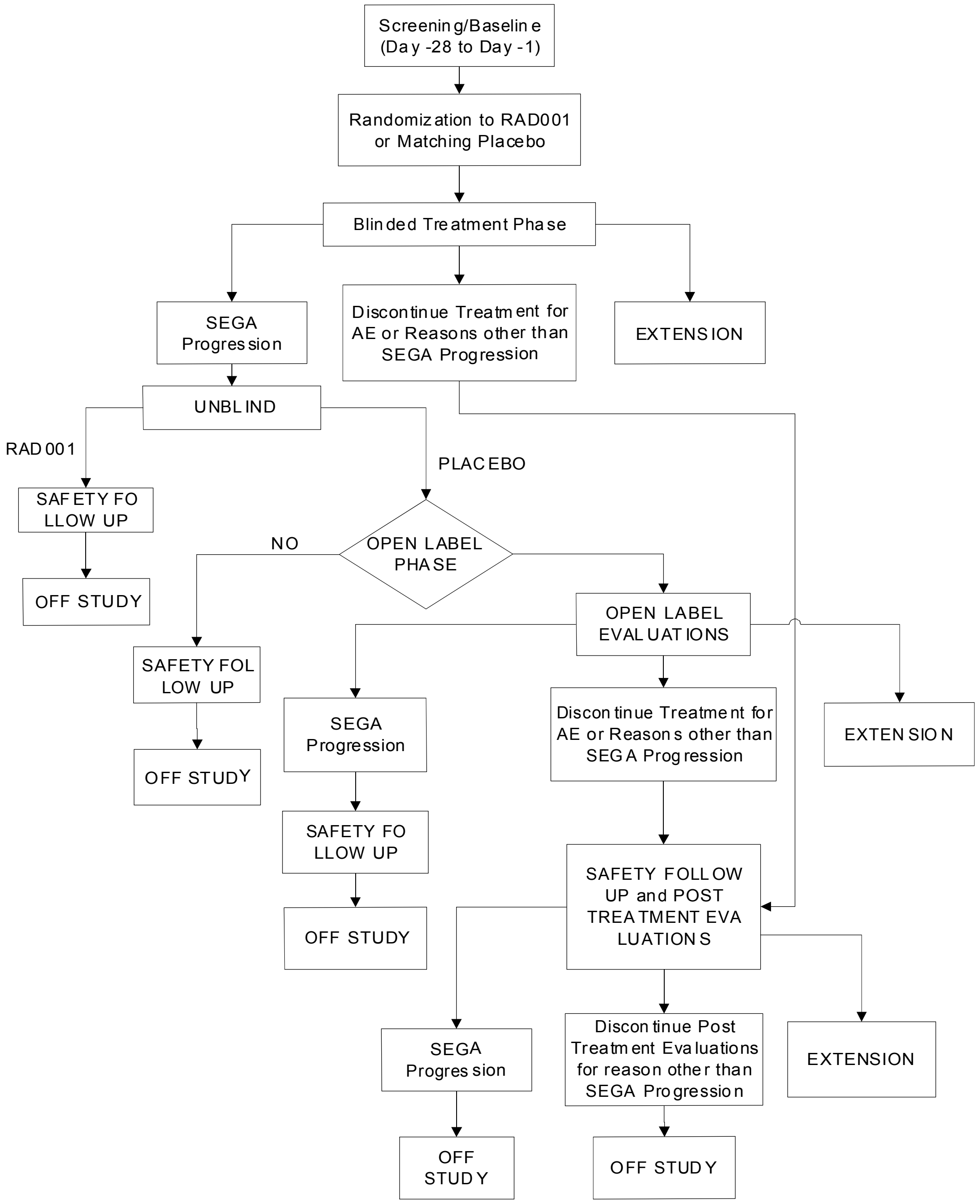

Safety Follow Up: *Collection of AEs and SAEs that occur within 28 days following treatment discontinuation. SAEs suspected to be related to study drug will be collected for an additional 8 weeks (56 days).*
Post Treatment Evaluations: *MRI of the brain (and MRI/CT of the kidneys, if applicable) annually.*
Open Label Evaluations: *MRI of the brain (and MRI/CT of the kidneys, if applicable) to be done 12, 24 and 48 weeks after the start of open label RAD001 and annually thereafter. Safety and efficacy assessments to be carried out as in the blinded treatment phase (with the exception of biomarker assessments, which will not be done in open label phase.)*

5 Population

The target population is comprised of patients of any age who have been diagnosed with TSC-associated SEGAs and have radiological evidence of one of the following three conditions prior to randomization: 1) serial growth, or 2) presence of a new SEGA lesion, or 3) new or worsening hydrocephalus. Further details can be found in the inclusion/exclusion criteria.

It is anticipated that approximately 125 subjects will need to be screened to enroll at least 99 patients. Subjects will be recruited from approximately 25 sites worldwide.

Inclusion/exclusion criteria

The investigator or his/her designee must ensure that all patients who meet the inclusion and exclusion criteria during screening are offered enrollment in the study.

No additional exclusions can be applied by the investigator, in order that the study population will be representative of all eligible patients.

Patients must have screening evaluations performed to ensure potential patients being considered by the investigator meet all inclusion and exclusion criteria. The investigator or his/her designee must review the results of all screening evaluations, to ensure that all inclusion and exclusion criteria have been satisfied prior to randomization of that patient into the study. Only laboratory results from the Central Laboratory will be used to determine patient eligibility for the study.

All study patients must be thoroughly informed about all aspects of the study, including the study visit schedule and required evaluations, and all regulatory requirements for informed consent. The written informed consent must be obtained prior to the performance of any screening evaluations. If the patient is unable to read, an impartial witness and/or the patient's parent or legal guardian must be present during the entire informed consent discussion. The following criteria apply to all patients enrolled into the study.

5.1 Inclusion criteria

5.2 Inclusion criteria

1. Male or female of any age.
2. Clinically definite diagnosis of tuberous sclerosis according to the modified Gomez criteria (Roach et al 1998; Hyman and Whittemore 2000, Table 5-1). Clinically definite diagnosis is defined as either of the following:
   a. Two Major Features from Table 5-1.
   b. One Major Feature plus two Minor Features from Table 5-1.
3. Presence of at least one SEGA lesion ≥ 1.0 cm in its longest diameter using MRI. **Note:** SEGA lesions are only diagnosed in patients with TSC. They arise in the subependymal layer of the lateral ventricle and are usually located near the foramen of Monro and enhance homogeneously with contrast on MRI with no evidence of surrounding edema.

4. A recent MRI of the brain completed within 4 weeks (28 days) of the patient's randomization must be compared with an MRI of the brain performed at an earlier stage of patient care (pre-baseline) and should demonstrate at least one of the following:
   a. Serial growth, defined as at least a 25% increase in SEGA volume, or
   b. Presence of a new SEGA lesion ≥ 1 cm in its longest diameter, or
   c. New or worsening hydrocephalus defined by assessment of ventricular configuration changes, ventricular cap signs (periventricular edema) and qualitative assessment of CSF flow dynamics.

Notes:

Patients who have had previous SEGA surgery are eligible provided criterion 4 has been satisfied by comparing the baseline scan to any prebaseline scan that has been conducted following the most recent SEGA surgery. If a previous MRI is not available, a comparative review of two previous CT scans is also acceptable to establish any of the above-mentioned three conditions. In this case, a baseline/screening MRI should still be performed.

If only one prior CT scan was available, a second CT scan should be obtained to allow like to like comparison of cranial images. Again, the baseline MRI should still be performed. CT scans must be digitized and sent to the central reviewer for confirmation of eligibility.

If the pre-baseline MRI or CT scan was only available on film (non-digital format) or software for volumetric assessment was not available at the local site, the local radiologist should make a qualitative assessment of the above-mentioned three criteria. The non-digital MRI or CT scan must also be sent to Central Radiology for review.

5. If female and of child-bearing potential, documentation of negative pregnancy test prior to enrollment. Sexually active pre-menopausal female patients must use highly effective contraceptive measures, while on study and for up to 8 weeks after ending treatment.
6. Written informed consent according to local guidelines.

Table 5-1 Diagnostic Criteria for Tuberous Sclerosis Complex

**Major Features**
1. Facial angiofibromas or forehead plaque<br>2. Nontraumatic ungual or periungual fibroma<br>3. Hypomelanotic macules (three or more)<br>4. Shagreen patch (connective tissue nevus)<br>5. Multiple retinal nodular hamartomas<br>6. Cortical tuber[a]<br>7. Subependymal nodule<br>8. Subependymal giant cell astrocytoma<br>9. Cardiac rhabdomyoma, single or multiple<br>10. Lymphangiomyomatosis[b]<br>11. Renal angiomyolipoma[b]
**Minor Features**
1. Multiple, randomly distributed pits in dental enamel<br>2. Hamartomatous rectal polyps[c]<br>3. Bone cysts[d]<br>4. Cerebral white matter radial migration lines[a,d]<br>5. Gingival fibromas<br>6. Non-renal hamartoma[c]<br>7. Retinal achromic patch<br>8. 'Confetti' skin lesions<br>9. Multiple renal cysts[c]
**Definite Tuberous Sclerosis Complex:**<br>**Either two Major Features or one Major Feature plus two Minor Features.**
a. The co-occurrence of cerebral cortical dysplasia and cerebral white matter radial migration lines should be considered as one major feature of TSC.<br>b. In patients with both lymphangiomyomatosis and renal angiomyolipoma, another feature of TSC must be identified before a **definite** diagnosis is assigned.<br>c. Histological confirmation of these features is suggested.<br>d. Radiographic confirmation of these features is sufficient.

5.3 Exclusion criteria

1. Patients for whom SEGA related surgery is likely to be required, in the opinion of the investigator.
2. History of myocardial infarction, angina or stroke related to atherosclerosis.
3. Known impaired lung function (e.g. FEV_1 or DL_{CO} $\leq$ 70% of predicted)
4. Significant hematological or hepatic abnormality (i.e., transaminase levels > 2.5 x ULN or serum bilirubin >1.5 x ULN, hemoglobin < 9 g/dL, platelets < 80,000/ mm^3, absolute neutrophil count < 1,000/mm^3).
5. Pregnancy or breast feeding.

6. Intercurrent infection at date of randomization.
7. Prior history of organ transplantation.
8. Recent surgery (involving entry into a body cavity or requiring sutures) within the 2 months prior to randomization.
9. Prior therapy with mTOR inhibitors (e.g. sirolimus, temsirolimus, everolimus).
10. Use of an investigational drug within the 30 days prior to randomization.
11. Uncontrolled hyperlipidemia: Fasting serum cholesterol > 300 mg/dL OR > 7.75 mmol/L AND Fasting triglycerides > 2.5 x ULN.
12. Uncontrolled diabetes mellitus as defined by fasting serum glucose > 1.5 x ULN.
13. Patients with bleeding diathesis or on oral anti-vitamin K medication (except low dose warfarin).
14. Patients with known history of HIV seropositivity.
15. Inability to attend scheduled clinic visits.
16. For the purpose of MRI assessments:
    a. Ferromagnetic metal implants other than those approved as safe for use in MR scanner (e.g., braces, some types of aneurysm clips, shrapnel).
    b. Patients suffering from uncontrollable claustrophobia or physically unable to fit into the machine (e.g., obesity, etc).

    Note: patients with vagal nerve stimulators are permitted to have CT assessments of angiomyolipomas unless local or national regulations do not permit this.
17. Serum creatinine > 1.5 x ULN.
18. History of malignancy in the past two years, other than squamous or basal cell skin cancer.
19. Any severe and/or uncontrolled medical conditions which could cause unacceptable safety risks or compromise compliance with the protocol, such as:
    a. ≥Grade 3 hypercholesterolemia/hypertriglyceridemia or ≥ Grade 2 hypercholesterolemia/hypertriglyceridemia with history of coronary artery disease (despite lipid-lowering treatment if given)
    b. Impairment of gastrointestinal function or gastrointestinal disease that may significantly alter the absorption of study drug (e.g., ulcerative disease, uncontrolled nausea, vomiting, diarrhea, malabsorption syndrome).
    c. Active skin, mucosa, ocular or GI disorders of Grade > 1

6 Treatment

6.1 Investigational and control drugs

The investigational drug used in the course of this trial is RAD001 (everolimus); the control drug used in this trial is matching placebo.

Definition of terms:

- Study treatment = Study drug = RAD001 or Matching Placebo

In both treatment arms, the study drug will be given by continuous oral daily dosing of one or more tablets.

Medication labels for study drug will comply with the legal requirements of each country and be printed in local language. They will supply no information about the patient. The storage conditions for study drug will be described on the medication label.

6.1.1 Known undesirable effects of study drug/treatment

Adverse events most frequently observed with RAD001 are rash, stomatitis /oral mucositis, non-infectious pneumonitis, fatigue, headache, anorexia, nausea, vomiting, diarrhea, and infections. Overall, the most frequently observed laboratory abnormalities include neutropenia, thrombocytopenia, hypercholesterolemia, and/or hypertriglyceridemia. The majority of these AEs have been of mild to moderate severity (NCI CTC grade 1-2). Recommendations for dose adjustments, should any of these treatment-related adverse events occur, are given in Table 6-1.

Management of infections

RAD001 is an immunosuppressant. Patients taking RAD001 are therefore at an increased risk of infection. In oncology patients, some infections have been severe, and rarely have had a fatal outcome. Physicians should be aware of the increased risk of infection, and should warn patients and their caregivers to be vigilant for signs and symptoms of infection, and to seek medical attention immediately should such signs or symptoms occur. Should an infection occur, anti-infectives should be prescribed as clinically appropriate, and in the case of clinically significant infection, consideration should be given to withholding study medication until resolution of the infection.

Management of stomatitis/oral mucositis/mouth ulcers

Stomatitis/oral mucositis/mouth ulcers due to RAD001 should be treated using appropriate locally available supportive care. Please note that investigators in earlier trials have described the oral toxicities associated with RAD001 as mouth ulcers, rather than mucositis or stomatitis. If your examination reveals mouth ulcers rather than a more general inflammation of the mouth, please classify the adverse event as such. Please follow the paradigm below for treatment of stomatitis/oral mucositis/mouth ulcers:

1. For mild toxicity (grade 1), use conservative measures such as **non-alcoholic mouth wash or salt water (0.9%) mouth wash** several times a day until resolution.
2. For more severe toxicity (grade 2 in which case patients have pain but are able to maintain adequate oral alimentation, or grade 3 in which case patients cannot maintain adequate oral alimentation), the suggested treatments are **topical analgesic mouth treatments (i.e., local anesthetics such as benzocaine, butyl aminobenzoate, tetracaine hydrochloride, menthol, or phenol)** with or without **topical corticosteroids,** such as triamcinolone oral paste 0.1% (Kenalog in Orabase®).
3. Agents containing hydrogen peroxide, iodine, and thyme derivatives may tend to worsen mouth ulcers. It is preferable to avoid these agents.

4. Antifungal agents must be avoided unless a fungal infection is diagnosed. In particular, systemic imidazole antifungal agents (ketoconazole, fluconazole, itraconazole, etc.) should be avoided in all patients due to their strong inhibition of RAD001 metabolism, therefore leading to higher RAD001 exposures. Therefore, topical antifungal agents are preferred if an infection is diagnosed. Similarly, antiviral agents such as acyclovir should be avoided unless a viral infection is diagnosed.

Management of hyperlipidemia and hyperglycemia

Management of hyperlipidemia should take into account the pre-treatment status and dietary habits of the patient. Blood tests to monitor hyperlipidemia must be taken in the fasting state. Hyperlipidemia and hypertriglyceridemia should be treated according to local best clinical practice. Grade 3 hypercholesterolemia (> 400 mg/dL or 10.34 mmol/L) or grade 3 hypertriglyceridemia (>5 × ULN) should be treated as clinically indicated with a 3-hydroxy-3-methyl-glutaryl (HMG)-CoA reductase inhibitor (e.g., atorvastatin, pravastatin) or appropriate lipid-lowering medication, in addition to diet. Patients should be monitored clinically and through serum biochemistry for the development of rhabdomyolysis and other adverse events as required in the product label/data sheets for HMG-CoA reductase inhibitors.

Note: Concomitant therapy with fibrates and an HMG-CoA reductase inhibitor is associated with an increased risk of a rare but serious skeletal muscle toxicity manifested by rhabdomyolysis, markedly elevated creatine phosphokinase (CPK) levels and myoglobinuria, acute renal failure and sometimes death. The risk versus benefit of using this therapy should be determined for individual patients based on their risk of cardiovascular complications of hyperlipidemia.

Hyperglycemia has been reported in clinical trials. Monitoring of fasting serum glucose is recommended prior to the start of everolimus therapy and periodically thereafter. Optimal glycemic control should be achieved before starting trial therapy.

Management of diarrhea

Diarrhea attributed to RAD001 toxicity may be treated with loperamide. Other medications for diarrhea may be used as needed.

Management of amenorrhea

Investigators should be aware of the identified risk of secondary amenorrhea in post-adolescent females. No changes in study drug treatment or treatment with concomitant medications was implemented in prior cases. Nearly all previous cases of amenorrhea for patients on study resolved without treatment action. Amenorrhea did not result in any treatment discontinuations. If amenorrhea event of at least 6 months is seen, consulations with an endocrinologist, gynecologist or other appropriate personnel are recommended.

6.1.2 Dosing modifications in case of treatment-related toxicities

Table 6-1 and Table 6-2 provide the procedure to be followed for dose modification and re-initiation of study treatment in the event of toxicities suspected to be related to the study treatment.

Table 6-1 Everolimus dose modification guidelines for non-hematologic toxicities

Toxicity	Actions
Pneumonitis	See Table 6-3
Hyperlipidemia and/or hypertriglyceridemia	Any grade:<br>Treat according to best clinical practice. No specific dose reductions are needed.
Hyperglycemia	Any grade:<br>Treat according to best clinical practice. No specific dose reductions are needed.
Stomatitis	Grade 2:<br>Interrupt study drug until resolution to ≤ grade 1. Restart at the same dose.<br>Grade 3:<br>Interrupt study drug until recovery to grade ≤1. Reintroduce study drug at the next lower dose level**. Discontinue study drug if stomatitis doesn't recover to ≤grade 1 within 4 weeks.<br>Grade 4:<br>Discontinue study drug.
Other Toxicities	Grade 2 and 3<br>Interrupt administration until resolution to ≤ grade 1.<br>Restart at the same dose.<br>Grade 4<br>Hold study drug until recovery to ≤ grade 1.<br>Reintroduce study drug at the lower dose level**, if available.
Toxicity requiring interruption for >6 weeks	Permanently discontinue treatment.
No specific dose adjustments are recommended for Grade 1 toxicity. However, physicians should always manage patients according to their medical judgment based on the particular clinical circumstances.<br>** To determine the next lowest dose level, please refer to Table 6-4. Due to rounding, the next lowest dose level may not result in an actual dose reduction (e.g. previous dose calculation was 4.4 mg resulting in a dose of 4 mg/d and the dose at the next lowest level results in a dose calculation of 3.6, still resulting in a 4 mg/d dose). In such cases, the patient's dose should be lowered by 1 mg.	

Table 6-2 Dose modification guidelines for hematologic toxicities

Toxicity	Actions
Thrombocytopenia<br>Platelet count	**≥ 75000/mm^3:** No change<br>**50000/ mm3 to 75000/ mm^3**<br>Hold study drug until recovery to **≥ 75000/mm^3**<br>Reintroduce study drug at the same dose level<br>**< 50000/ mm^3**<br>Hold study drug until recovery to **≥ 75000/mm^3**<br>Reintroduce everolimus at the next lower dose level**, if available.
Absolute Neutrophil Count (ANC)	**≥ 1000/ mm^3**: No change<br>**500/ mm3 to 1000/ mm^3**<br>Hold study drug until recovery to ≥ 1000/mm^3<br>Reintroduce study drug at the same dose level<br>**< 500/ mm^3**<br>Hold until recovery to ≥ 1000/ mm^3. Reintroduce study drug at the next lowest dose level**, if available.
Febrile neutropenia	Hold further dosing until ANC ≥ 1250/mm^3 and no fever.<br>Then resume dosing at the next lower dose level** if available.
Toxicity requiring interruption for > 6 weeks	Discontinue study treatment.
Physicians should always manage patients according to their medical judgment based on the particular clinical circumstances.	

Management of non-infectious pneumonitis

Non-infectious pneumonitis is a known side effect of rapamycin analogues including RAD001. Clinically significant pneumonitis is typically accompanied by non-specific symptoms including dyspnea, nonproductive cough, fatigue, and fever. Diagnosis is generally suspected in individuals receiving mTOR inhibitors who develop these symptoms or in asymptomatic individuals in whom a routine chest CT scan reveals a new ground glass or alveolar infiltrate. The frequency of symptomatic pulmonary toxicity (all grades) was approximately 13% in a phase III study of RAD001 in patients with metastatic renal cell carcinoma ([CRAD001C2240]). Severe (CTC grade 3) pneumonitis occurred in 4% of patients, and an occasional fatality was reported. The lung toxicity was partly or completely reversible in the majority of cases with interventions including drug interruption, discontinuation and the use of corticosteroids.

Individuals participating in this trial will be questioned at each visit as to the presence of new or changed pulmonary symptoms consistent with lung toxicity. If an investigator suspects a patient may be developing pneumonitis, the patient should be managed according to Table 6-3. Investigations such as pulmonary function tests, CT chest and referral to a pulmonologist should be considered. Pulmonary function studies will be performed and interpreted using the ATS/ERS guidelines (Brusasco et al 2005).

Table 6-3 Management of non-infectious pneumonitis

Worst Grade Pneumonitis	Required Investigations	Management of Pneumonitis	Study Treatment Dose Adjustment
Grade 1	CT scans with lung windows. Repeat CT scan at least every 12 weeks until return to within normal limits.	No specific therapy is required.	Administer 100% of study treatment dose.
Grade 2	CT scan with lung windows. Consider pulmonary function testing including spirometry, DL_{CO}, and room air O_2 saturation at rest. Repeat CT scan at least every 12 weeks until return to within normal limits. Consider bronchoscopy with biopsy and /or BAL	Symptomatic only. Consider corticosteroids if symptoms are troublesome.	Reduce study treatment dose by 1 dose level** (see Table 6-4) until recovery to ≤ Grade 1. Study treatment may also be interrupted if symptoms are troublesome. Patients will discontinue study treatment if they fail to recover to ≤ Grade 1 within 3 weeks.
Grade 3	CT scan with lung windows and pulmonary function testing including spirometry, DL_{CO}, and room air O_2 saturation at rest. Repeat at least every 8 weeks until return to within normal limits. Bronchoscopy with biopsy and/or BAL is recommended.	Consider corticosteroids if infective origin is ruled out. Taper as medically indicated.	Hold treatment until recovery to ≤ Grade 1. May restart study treatment within 3 weeks at a reduced dose (by one level**) if evidence of clinical benefit.
Grade 4	CT scan with lung windows and required pulmonary function testing including spirometry, DL_{CO}, and room air O_2 saturation at rest. Repeat at least every 8 weeks until return to within normal limits. Bronchoscopy with biopsy and/or BAL is recommended if possible.	Consider corticosteroids if infective origin is ruled out. Taper as medically indicated.	Discontinue treatment.
**To determine the next lowest dose level, please refer to Table 6-4. Due to rounding, the next lowest dose level may not result in an actual dose reduction (e.g. previous dose calculation was 4.4 mg resulting in a dose of 4 mg/d and the dose at the next lowest level results in a dose calculation of 3.6, still resulting in a 4 mg/d dose). In such cases, the patient’s dose should be lowered by 1 mg.			

Follow-up for toxicities

Patients whose treatment is interrupted or permanently discontinued due to an adverse event or abnormal laboratory value suspected to be related to study treatment must be followed at least weekly until the adverse event or abnormal laboratory resolves or returns to grade 1. If a patient requires a dose delay of ≥ 6 weeks from the intended day of the next scheduled dose, then the patient must be discontinued from the study.

All patients will be followed for adverse events and serious adverse events for 28 days following the last dose of study drug. Beyond these 28 days, any serious adverse events that are suspected to be related to the study drug and occur within the next 8 weeks (56 days) will also be collected. Any medication/therapy given during these 12 weeks will be recorded on the CRF.

6.1.3 Study drug

The study drug is RAD001 or matching placebo.

6.1.3.1 How supplied

Each study site will be supplied by Novartis with study drug in identically-appearing packaging. One component of the packaging has a 2-part label. Each part of this label contains a medication number corresponding to one of the 2 treatment groups. Investigator staff will identify the study drug package to dispense to the patient by logging onto IWRS and obtaining the medication number. Immediately before dispensing the package to the patient, investigator staff will detach the outer part of the label from the packaging and affix it to the source document (Drug Label Form) containing that patient's unique patient number.

For the duration of the trial, RAD001 or matching placebo will be supplied by Novartis at no charge to each study site. In the extension phase of the study, open-label drug will be supplied.

6.1.3.2 Preparation and storage

RAD001 is formulated as tablets of 1.0-mg strength, blister-packed under aluminum foil in units of 10 tablets and dosed on a daily basis. Matching placebo will be provided as a matching tablet and will also be blister-packed under aluminum foil in units of 10. RAD001 or matching placebo tablets should be opened only at the time of administration as active drug is both hygroscopic and light-sensitive.

6.1.3.3 Active control

Not applicable for this study.

6.2 Treatment arms

There are two treatment arms in this study: RAD001 or Matching Placebo. Each patient will be randomized into one treatment arm at the start of the study (**and patients should start treatment as soon as possible after randomization and no more than 7 days after randomization**).

6.3 Patient numbering

Each patient in the study is uniquely identified by a **9-digit patient number** which is a combination of his/her **4-digit center number** and **5-digit subject number**. The center number is assigned by Novartis to the investigative site.

When the patient has signed the informed consent form, the investigator or his/her staff will log onto the IWRS and provide the requested patient identifying information including the patient number. The assigned 5-digit subject number alone (excluding the leading zeros) should be entered on the CRF in the field labeled "Subject ID". Once assigned to a patient, the patient number will not be reused for another patient. If the patient fails to be randomized, the patient's status as a screen failure should be updated in the IWRS as soon as possible. In addition, the Screening Log should be completed for these patients. Patients who screen fail and are subsequently eligible for trial participation (i.e. now eligible due to a protocol amendment) should be rescreened under the same 9-digit patient number.

Informed consent must be obtained before any testing is performed to determine a patient's eligibility.

6.4 IWRS procedure

User Acceptance Testing of the IWRS based on test data will be performed by the project team prior to its implementation. IWRS will provide contact information and detailed instructions on registration and randomization procedures to each study site. **During the study, a fax confirmation of every transaction of the web-based system will be issued as documentation.**

- At visit 1 (screening/baseline) the investigator/study coordinator will log onto IWRS to register the patient.
- If the patient is eligible to be randomized, just prior to visit 2 (Treatment Day 1) the investigator will log onto IWRS to perform the randomization. **The investigator or his/her designee will log onto IWRS as close as possible to the initiation of therapy (Treatment Day 1, Visit 2)**. The investigator will select one of the protocol-specified EIAEDs (see Section 6.5) that the patient is taking and the randomization will be stratified based on this input.
- If a screened patient fails to be randomized, the IWRS must be updated with the patient's screen-failure status as soon as possible.
- The investigator or his/her designee will update IWRS immediately if a patient discontinues from study.
- The investigator or his/her designee will update IWRS for patient unblinding (with respect to emergency unblinding as well as for unblinding following SEGA progression [i.e., patients who are candidates for open-label RAD001 therapy]).
- No study medication should be dispensed without logging onto IWRS.
- During the trial, the central laboratory will update IWRS with trough PK information immediately after the analysis is completed. The actual drug levels will not be made available either to the investigators or to the Novartis clinical team.

- Based on PK information (and a randomized schema for patients on placebo), IWRS will immediately notify the investigator to adjust the dose, if necessary, and if the current dose was well tolerated. Patients on placebo will also have dose adjustments recommended via IWRS, in a randomized fashion, in order to maintain the blind.
- Patients who have a PK profile collected according to Amendment 5, will be logged and tracked within the IWRS system in order to monitor the number of patients in each age and co-administration of CYP3A4/PgP inducers group. The investigator will log into the appropriate module and enter the patient's ID, confirm steady state study drug dosing and use/non-use of CYP3A4/PgP inducers over the 7 days prior to the pre-dose sampling. A high level list of the CYP3A4/PgP inducers is provided in Table 6-5 and Table 6-6, but an exhaustive list of the CYP3A4/PgP inducers will be communicated to the investigators for the determination of the CYP3A4/PgP inducers status. The system will track how many patients have been entered into each age group (<5, 5-<10, 10-<14, 14-<18, ≥18 years at time of pre-dose sampling) and how many within each age group are receiving CYP3A4/PgP enzyme inducers over the 7 days prior to the pre-dose sampling.
- The investigator or his/her designee will update IWRS with any dose adjustments (increase or decrease) that occur for patients on study.
- Re-supply of trial therapy to the study sites will occur as needed according to the supply strategy defined in the IWRS URS (User Requirement Specification).
- During the trial, IWRS will immediately notify the Clinical Trial Head (CTH) or designee and the clinical monitor assigned to the site of any occurrence of emergency code breaks.
- Unblinding may occur after documented SEGA progression during the blinded treatment phase - this is to enable patients randomized to placebo to switch to open-label RAD001. Unblinding may also occur in the case of medical emergencies when the treating physician believes that the knowledge of the blinded treatment is essential. Complete details are provided in Section 6.7.6 for Emergency unblinding of treatment assignment.

6.5 Treatment assignment

On or prior to Visit 2 (Treatment Day 1) the investigator or his/her designee will log onto IWRS (after verifying that the patient fulfills all eligibility criteria) to randomize the patient. The IWRS will assign a randomization number to the patient, which will be used to link the patient to one of the two treatments, and will specify a unique medication number for the first package of study drug to be dispensed to the patient. The medication number appears on the study medication pack that will be dispensed to the patient. The randomization number will not be communicated to the caller.

The randomization will be stratified by use of enzyme-inducing anti-epileptic drugs (EIAED), with patients considered to be either EIAED-users or EIAED-non-users. An EIAED-user is defined as a patient who at randomization was receiving at least one of the following anti-epileptic drugs (considered strong inducers of CYP3A4):

1. Phenytoin (Dylantin®, Dilantin Kapseals®, Dilantin® Infatabs®, Eptoin®, Epanutin®, Diphenin®, Dipheninum®, Phenytek®)
2. Mephenytoin (Mesantoin®)

3. Carbamazepine (Tegretol®, Biston®, Calepsin®, Carbatrol®, Epitol®, Equetro®, Finlepsin®, Sirtal®, Stazepine®, Telesmin®,Teril®, Timonil®, Trimonil®, Epimaz®, and Degranol®)
4. Phenobarbital (Luminal®)
5. Pentobarbital (Nembutal®)
6. Primidone (Mysoline®)
7. Oxcarbazepine (Trileptal®)

6.6 Treatment blinding

This is a double-blind study. The study design allows for patient unblinding only in very precise circumstances. The clinical personnel at the Central Laboratory and at the Central Radiology will remain blinded to the identity of the treatment from the time of randomization until final database lock.

Randomization will be performed using the following procedures to ensure that treatment assignment is unbiased and concealed as best as possible from all individuals involved in the study: 1) randomization data are kept strictly confidential until the time of unblinding at either SEGA progression or at the time of final analysis, and will not be accessible to anyone involved in the conduct of the study with the exception of the DMC who will review safety data six months after the randomization of the first patient and every six months thereafter and, 2) the identity of the treatments will be concealed by the use of study drugs (RAD001 and Matching Placebo) that are identical in packaging, labeling, schedule of administration and in appearance. Patients on placebo will also have dose adjustments recommended via IWRS, in a randomized fashion, in order to maintain the blind.

The randomization list will be generated by the IWRS provider using a validated system that automates the random assignment of patient numbers to randomization numbers. The randomization numbers are linked to the two different treatment arms, which in turn are linked to medication numbers. The randomization scheme for patients will be reviewed and approved by a member of the Biostatistics Quality Assurance Group and will be locked and kept by them after its approval.

At the conclusion of the study, when the study data have been verified, and the protocol deviations have been determined and the database locked, the assigned blinded drug codes can be broken and made available to the sponsor for the final analysis of the study data.

6.7 Treating the patient

6.7.1 Study drug administration

RAD001 or matching placebo will be dispensed by the study center personnel on an outpatient basis. Patients will be provided with 5 weeks worth of study drug on Treatment Day 1, week 4 and week 8 for self administration at home. At later visits, trial therapy will be provided as needed. **On the days of PK sampling, RAD001 or matching placebo will only be administered by the investigator (or his/her designee) at the study site** AFTER **obtaining the trough blood PK sample.** Patients who enter the open-label phase of the study will be provided with RAD001, according to the dispensing schedule above, for self-administration at home.

The investigator should instruct the patient to take the study drug exactly as prescribed (promote compliance). All doses prescribed and dispensed to the patient and all dose changes during the study must be recorded on the Dosage Administration Record CRF.

RAD001 or matching placebo will be dosed starting on Treatment Day 1 (Visit 2). Patients will be instructed to take 4.5 mg/m^2 (rounded to the nearest milligram) of RAD001 or matching placebo orally once daily at the same time each day immediately after a meal. RAD001 or matching placebo tablets should be swallowed whole with a glass of water. The tablets should not be chewed or crushed. If the tablets can not be swallowed, the tablets should be disintegrated in approximately 30 mL of water. Immediately prior to administration, the contents should be stirred gently until the tablets have disintegrated into a suspension. The contents should then be drunk by the patient. Afterwards, the glass should be rinsed with an additional 30 ml of liquid and drunk by the patient. Any dietary habits around the time of RAD001 or matching placebo intake should be as consistent as possible throughout the study, and in particular, during those periods when blood samples are being taken for pharmacokinetic analyses. If vomiting occurs, no attempt should be made to replace the vomited dose.

Body surface area (BSA), in m^2, will be calculated by IWRS using the following formula, where weight (W) is in *kilograms* and height (H) is in *centimeters* (Dubois and Dubois 1916):

$$BSA = (W^{0.425} \times H^{0.725}) \times 0.007184$$

RAD001 or the matching placebo should be administered orally once daily at the same time every day immediately after a meal. If vomiting occurs, no attempt should be made to replace the vomited dose.

On days when blood will be drawn (scheduled visits), patients should ***not*** take the daily study drug dose **until after** blood is drawn so that an accurate trough level of RAD001 can be obtained. **On days of scheduled visits, patients should bring their daily dose of study drug into the clinic with them for administration after blood is drawn.** In the absence of any other reason for holding study drug, study drug may be continued for up to 2 weeks while awaiting central lab results, which will be used to determine if a modification of the dosing regimen is required.

Patients should be requested to bring their unused study drug, including the empty blister packs, to the clinic at each visit. Compliance should be verified by the investigator's staff through counting the number of tablets consumed between visits. The investigator (or his/her designee) will document dosage administration and all dose changes during the study on the CRF. The site must maintain an overall drug accountability log for the study, as well as individual accountability records for each patient. The dose, amount dispensed, amount received, and amount unused must be recorded in the source document. Drug accountability will be noted by the field monitor during site visits and at the completion of the study. The patient will return all unused study drug at each dispensing visit and at the end of the study.

Patients will receive treatment with study drug until SEGA progression (see definition in Section 7.5.2), the occurrence of unacceptable toxicity, or until the investigator or patient decides that continuation is not in the best interest of the patient. Interruption for toxicity should follow the instructions in Table 6-1, Table 6-2 and Table 6-3.

6.7.2 Permitted study drug adjustments

For patients who are unable to tolerate the protocol-specified dosing schedule, dose adjustments are permitted in order to keep the patient on study drug. The guidelines set forth in Table 6-1, Table 6-2 and Table 6-4 should be followed. In addition, if any surgery is planned, trial therapy should be interrupted one week prior to surgery and should be re-started as soon as possible after wound healing

If treatment is interrupted due to toxicity, study drug should not be resumed unless recovery to grade ≤1 is achieved in less than 6 weeks. Then it could be reintroduced at the initial dose or a lower dose level depending on the toxicity type and grade (see Table 6-1 and Table 6-2). These changes must be recorded on the Dosage Administration Record CRF. If treatment is interrupted for 6 weeks or more, the patient should be discontinued from the study.

The relationship between RAD001 trough concentrations and dose has been elaborated, and shown in previous studies to be linear, consistent with the dose proportionality seen with AUC (see Section 1.3.4.1), providing the quantitative basis for adjusting the RAD001 dose as needed to achieve the desired 5 - 15 ng/mL trough target concentration. Each patient will start with a dose of 4.5 mg/m^2/day. Trough (pre-dose) blood levels of RAD001 will be assessed after 2 weeks. A local lab (at the treating center) will collect the blood samples and send them to a central laboratory to perform the PK analysis. The central laboratory will upload PK information for each patient directly into the IWRS. Based on PK information (and a randomized schema for patients on placebo), IWRS will immediately notify the investigator to adjust the dose. The notification will advise the investigator to either maintain, decrease or increase the dose of trial therapy according to Table 6-4, in order to achieve a blood trough level of 5-15 ng/mL. The investigator will only consider maintaining or increasing the dose if the current dose is well tolerated. Note that if the blood trough level is above 15 ng/mL, then IWRS will advise the investigator to reduce the dose to the next lowest dose level according to Table 6-4 (which should be implemented by the investigator, even if the current dose is well tolerated). Blood levels of RAD001 will be assessed 1-2 weeks after any dose increase to a new level, or any decrease in an enzyme-inducing drug, or any increase in an enzyme-inhibiting drug.

In addition to trough blood levels (pre-dose) that will be assessed at each clinic visit, 1-2 weeks after starting an increased dose to a new level, or any decrease in an enzyme-inducing drug, or any increase in an enzyme-inhibiting drug an additional blood sample (2 mL) will be taken 2 hours (± 30 mins) after taking the trial therapy dose.

In order to maintain the blind, the drug therapy dose adjustments described above will apply to all patients, including those on placebo (in a randomized fashion through IWRS). IWRS will refer to a randomization list to arbitrarily recommend decreasing, maintaining or increasing the dose. The investigator will only consider maintaining or increasing the dose if the current dose is well tolerated.

Other dose adjustments (reduction, interruption or possible dose re-escalation to starting dose) will occur based on safety findings. When the dose is reduced due to a toxicity and then subsequently increased (to the pre-toxicity dose level) following resolution, a 2-week post increase PK collection is not required, since the patient would have already had PK assessments at this dose level. All doses taken by the patient and all dose changes during the study must be recorded on the Dosage Administration Record CRF.

Table 6-4 RAD001 dose levels for dose adjustment

Dose level	Daily dose of RAD001 or Placebo (mg/m^2)
-3 (-50% of Dose Level -2)	2.53 (every other day)
-2 (-25% of Dose level -1)	2.53
-1 (-25% of Starting Dose)	3.38
Starting Dose	4.50
+1 (+33% of Starting Dose)	6.00
+2 (+33% of Dose Level +1)	8.00
+3 (+33% of Dose Level +2)	10.67
+4 (+33% of Dose Level +3)	14.22

NOTE: Due to rounding, the next lowest dose level may not result in an actual dose reduction (e.g. previous dose calculation was 4.4 mg resulting in a dose of 4 mg/d and the dose at the next lowest level results in a dose calculation of 3.6, still resulting in a 4 mg/d dose). In such cases, the patient's dose should be lowered by 1 mg.

If a patient has already decreased 3 dose levels due to toxicity (Adverse Event), no further dose reduction is permitted. Patients requiring a fourth dose reduction due to toxicity (Adverse Event) will be required to discontinue study treatment.

6.7.3 Concomitant medications

Patients must be instructed not to take any additional medications (over-the-counter, herbal or other products) during the study without prior consultation with the investigator. All medications taken within 30 days of starting study treatment should be reported on the Concomitant Medications/Significant Non-drug Therapies CRF. The following guidelines must be adhered to during the study:

- Investigational or commercial anti-proliferative agents other than study drug (including other mTOR inhibitors, e.g., sirolimus, temsirolimus) are prohibited.
- Co-administration with moderate or strong inhibitors of CYP3A4 (e.g., ketoconazole, itraconazole, ritonavir, erythromycin, fluconazole) or inhibitors of P-glycoprotein (PgP) must be avoided (refer to Table 6-5 and Table 6-6)
- Co-administration with strong inducers of CYP3A4, other than antiepileptics, must be avoided
- Seville orange, star fruit, grapefruit and their juices affect P450 and PgP activity. Concomitant use should be avoided

Table 6-5 lists clinically relevant CYP3A inhibitors, inducers and the definition of strong and moderate inhibitors/inducers.

RAD001 may affect patient response to vaccinations making the vaccination less effective. As RAD001 is an immunosuppressant, live vaccines should be avoided while a patient is treated with RAD001.

Otherwise, the use of other concomitant medication/therapy deemed necessary for the care of the patient is allowed. The investigator should instruct the patient to notify the study site about any new medications he/she takes after the start of the study drug. All medications (other than study drug) and significant non-drug therapies (including physical therapy and blood transfusions) administered after the patient starts RAD001 and for up to 84 days (12 weeks) after study drug discontinuation must be listed on the Concomitant Medications/Significant Non-drug Therapies CRF .

Table 6-5 Clinical relevant drug interaction: inducers and inhibitors of isoenzyme CYP3A

INDUCERS
Barbiturates, carbamazepine, glucocorticoids, modafinil, oxcarbazepine, phenobarbital, phenytoin, pioglitazone, rifabutin, rifampin, St. John's wort, troglitazone, efavirenz, nevirapine, topiramate
INHIBITORS
Strong inhibitors:<br>clarithromycin, conivaptan, indinavir, itraconazole, ketoconazole, lopinavir, mibefradil, nefazodone, nelfinavir, ritonavir, saquinavir, telithromycin, troleandamycin, voriconazole,<br>Posaconazole (Krishna et al 2009)<br>Moderate inhibitors:<br>aprepitant, atazanavir, cimetidine, ciprofloxacin, darunavir, diltiazem, erythromycin, fluconazole, grapefruit juice, imatinib, tofisopam, verapamil,

Table 6-6 Clinical relevant drug interactions medicated by PgP

PgP Substrates	PgP Inhibitors in vivo	PgP Inducers
digoxin, fexofenadine, indinavir, vincristine, colchicine, topotecan, paclitaxel	amiodarone, azithromycin, captopril, carvedilol, clarithromycin, conivaptan, cyclosporine, diltiazem, elacridar, erythromycin, felodipine, (GF120918), itraconazole, ketocoanzole, lopinavir, (LY335979), mibefradil, nifedipine, nitrendipine, (PSC833), quinidine, ranolazine, ritonavir, talinolol, valspodar, verapamil	rifampin, St John's wort
Reference:<br>Internal Clinical Pharmacology Drug-drug interaction (DDI) memo, updated Dec. 2, 2009, which summarizes DDI data from three sources including the FDA's "Guidance for Industry, Drug Interaction Studies, the University of Washington's Drug Interaction Database, and Indiana University School of Medicine's Drug Interaction Table "<br>**This list of clinically relevant drug interactions is updated as of December 02, 2009**		

6.7.3.1 Concomitant Medication dose adjustments

Everolimus trough levels are taken routinely at every clinic visit.

Samples for trough (24 h after the last dose) and C_{max} (2.0 hours ± 30 mins after dosing) will be taken two weeks after the following events:

1. everolimus dose increase to a higher level than previously taken
2. reduction in the dose of a CYP3A4 or PgP inducer (e.g. reduction in anticonvulsant dose)
3. starting, or increasing the dose of, a CYP3A4 or PgP inhibitor

Trough and C_{max} are not required for dose re-escalation to a previously-used dose; or for dose decreases. Patients in these situations will have sampling done at the next routine visit.

6.7.4 Study drug interruption or discontinuation

The term "interruption" refers to a patient stopping the study medication during the course of the study, but then re-starting it at a later time in the study.

The term "discontinuation" refers to a patient's premature and permanent withdrawal from the study treatment. The reason for discontinuation from treatment will be recorded. The patient may discontinue participation in the study for any of the following reasons:

- adverse event(s)
- abnormal laboratory value(s)
- abnormal test procedure result(s)
- SEGA progression (defined in Section 7.5.2)
- protocol deviation
- subject withdrew consent
- lost to follow-up
- administrative problems
- death
- new treatment for indication under study
- treatment duration completed as per protocol (only to be used at end of extension phase)
- final primary analysis (only to be used when results of final analysis are known and the decision is made on whether to launch the extension phase)

If a patient has discontinued the study drug due to an unacceptable adverse event (AE) or an abnormal laboratory value, he/she should not have withdrawal of consent recorded as the reason for discontinuation. Instead, the reason for discontinuation must be recorded as due to an AE or abnormal laboratory value.

Patients who discontinue the study drug regardless of the reason must have end of study treatment evaluations (Refer to Table 7-1 and/or Table 7-2, End of treatment) on the Day of study treatment discontinuation or within 1 week of study treatment discontinuation. The investigator or his/her designee will proceed as follows:

- Update IWRS immediately with any patient discontinuations.

- Complete the end of treatment evaluations (additional details are provided in Table 7-1 or Table 7-2 and the End of Treatment CRF indicating the date and reason for stopping the study drug.
- All patients will have a follow-up visit 28 days after the last dose of study treatment. During this visit, AE and SAE information will be collected and recorded on the appropriate CRFs. Patients will also be followed for an additional 8 weeks (56 days) for collection and recording of any SAEs that are suspected to be related to study drug. In addition, any medication/therapy taken during these 12 weeks will be recorded on the **Concomitant medications/Significant non-drug therapies** CRF page. If the patient is unable to return to the clinic, the investigator or his/her designee will contact the patient or caregiver via telephone to collect this information.
- All patients who are discontinued from study treatment for any reason other than SEGA progression will continue to have MRIs of the brain annually until the start of any non-study systemic anti-SEGA therapy withdrawal of cons**ent,** or until end of the study, whichever occurs first. Radiological studies will be sent for central review during this follow-up period within 2 days of the scan. The investigator or his/her designee will collect information on the initiation of additional anti-SEGA therapies every month. This information may be obtained during a telephone call and will be recorded in the source documents as well as on the appropriate CRFs.

All patients must have evaluations for 28 days after the last dose of study treatment and an additional 8 weeks (56 days) for collection and recording of any SAEs that are suspected to be related to study drug. Patients lost to follow-up should be recorded as such on the CRF. Patients who require SEGA-related surgery must discontinue the study drug before the surgery. Patients who discontinue study drug before completing the study should be scheduled for a visit as soon as possible, at which time all of the assessments listed for End of Treatment visit will be performed. At a minimum, all patients who discontinue study treatment, including those who refuse to return for a final visit, will be contacted for safety evaluations at 12 weeks (84 days) after the last dose of study drug.

6.7.5 Withdrawal from the study and study evaluation completion

Patients **may** voluntarily withdraw from the study or be dropped from it at the discretion of the investigator at any time.

As a general rule, if a patient discontinues study drug and later is withdrawn from the study, the reasons for study evaluation completion may include the following:

- Protocol deviation
- Subject withdrew consent
- Lost to follow-up
- Administrative problems
- Follow-up phase completed as per protocol

  Would be selected in any of the following scenarios:

  - A patient has not yet progressed at the time of final results if the study is negative and no extension phase will be launched.

  - A Patient who has not progressed at the end of the extension phase.
  - A patient who progressed on treatment and would be completed at the 28-day safety visit.
- Death
- New treatment for indication under study
- SEGA progression (defined in Section 7.5.2)

For patients who are lost to follow-up, the investigator should show due diligence by recording in the source documents steps taken to contact the patient, e.g., dates of telephone calls, registered letters, etc.

6.7.6 Emergency unblinding of treatment assignment

In general, circumstances that might lead to emergency unblinding are rare. Most often, study drug discontinuation and knowledge of the possible treatment assignments are sufficient to treat a study patient who presents with an emergency condition. One unusual circumstance in which unblinding might be necessary is when a patient requires emergency surgery and the anesthesiologist needs to know all medications that the patient has been exposed to in order to make proper decisions about treatment and support during the surgery.

Emergency unblinding should only be done when necessary in order to treat the patient. Emergency code breaks are performed using IWRS. When the investigator telephones the system to unblind a patient, he/she must provide the requested patient identifying information. The investigator will then receive details of the drug treatment for the specified patient and a fax confirming this information. The system will automatically inform the Novartis monitor for the site and the Clinical Trial Head (CTH) or designee that the code has been broken.

It is the investigator's responsibility to ensure that there is a procedure in place to allow access to IWRS in case of emergency. The investigator will inform the patient how to contact his/her backup in cases of emergency when he/she is unavailable. The protocol number, study drug name if available, patient number and instructions for contacting the local Novartis CPO (or any entity to which it has delegated responsibility for emergency code breaks) will be provided to the patient in case emergency unblinding is required at a time when the investigator and backup are unavailable.

Study drug must be discontinued after emergency unblinding. The investigator is not allowed to place emergency unblinded patients into open-label RAD001 therapy.

6.7.7 Treatment compliance

Compliance will be assessed by the investigator or his/her designee at each visit using pill counts. This information should be captured in the source document at each visit.

- Patients will be requested to bring their unused medication including empty packaging to the clinic at each visit.
- All doses taken by the patient and all dose changes during the study must be recorded on the Dosage Administration Record CRF.
- The investigator or his/her designee must keep documentation (overall drug accountability log for the study as well as individual study drug accountability records for each patient)

of tablets administered, tablets used, dose changes, dates dispensed and intervals between visits.

- Drug accountability will be monitored by the field monitor during site visits and at the completion of the study.

7 Visit schedule and assessments

Table 7-1 and Table 7-2 list all of the assessments and indicate with an "X" the visits when they are to be performed. All data obtained from these assessments must be supported in the patient's source documentation. The table indicates which data are entered into the database (D) or remain in source documents only (S). Assessments that generate data for database entry and are recorded on CRFs are listed using the CRF name. Assessments that are transferred to the database electronically (e.g., laboratory data) are listed by test name.

Tests, procedures and visits should occur on schedule whenever possible. However, tests, procedures, and visits that occur within the prescribed allowable windows indicated in Table 7-1 and Table 7-2 will not constitute protocol deviations

Table 7-1 Blinded phase visit evaluation schedule

Note: To be followed for all patients at the beginning the study until SEGA progression or discontinuation

**Assessment**	**Screening/ Baseline**	**Treatment Day 1**	**2 wks (± 2 Days)**	**4 wks (± 2 Days)**	**6 wks† (± 2 Days)**	**8 wks (± 2 Days)**	**12 wks (± 7 Days)**	**18 wks (± 7 Days)**	**24 wks (± 7 Days)**	**Every 4 wks thereafter (± 7 Days)**	**Every 12 wks thereafter (± 7 Days)**	**Every 24 wks thereafter (± 7 Days)**	**Annually (± 7 Days)**	**End of treatment (28 days after last dose)**	**Follow-Up**	**Study Completion**
**Time point (days)**	**-28 to -1**	**0**	**14**	**28**	**42**	**56**	**84**	**126**	**168**							**Last**
**Visit no.**	**1**	**2**	**3**	**4**	**5**	**6**	**7**	**8**	**9**					**777 or 779**		**780**
Written Informed Consent (S)	X															
Inclusion/Exclusion Criteria (D)	X	X														
IWRS Registration[a] (S)	X															
Demography (D)	X															
Medical History/Current Medical Conditions (D)	X	X														
HIV History (D)	X	X														
HBV-DNA, HBsAg, HBc Ab, HBs Ab, HCV-RNA-PCR∞(D)	X															
Serum pregnancy test[b] (D)	X													X		

Assessment	Screening/ Base-line	Treatment Day 1	2 wks (± 2 Days)	4 wks (± 2 Days)	6 wks[†] (± 2 Days)	8 wks (± 2 Days)	12 wks (± 7 Days)	18 wks (± 7 Days)	24 wks (± 7 Days)	Every 4 wks there-after (± 7 Days)	Every 12 wks there-after (± 7 Days)	Every 24 wks there-after (± 7 Days)	Ann-ually (± 7 Days)	End of treatment (28 days after last dose)	Follow-Up	Study Completion
**Time point (days)**	**-28 to -1**	**0**	**14**	**28**	**42**	**56**	**84**	**126**	**168**							**Last**
**Visit no.**	**1**	**2**	**3**	**4**	**5**	**6**	**7**	**8**	**9**					**777 or 779**		**780**
Urine pregnancy test[b] (D)		X		X		X	X		X	X	X					
Vital signs (D)	X	X		X		X	X	X	X		X			X		
Physical exam (including Neurological Exam)[c] (S)	X	X		X		X	X	X	X		X			X		
Endocrine Assessments$^{\Psi}$ (D)	X$^{\Psi}$						X$^{\Psi}$		X$^{\Psi}$		X$^{\Psi}$		X$^{\Psi}$	X$^{\Psi}$		
Performance status (WHO or Lansky)[d] (D)	X	X		X		X	X	X	X		X			X		
MRI of the Brain[e] (D)	X						X		X		X**			X		
CT/MRI of the Kidney[f] (D)	X						X		X		X**			X		
ECG[g] (D)	X													X		
Fasting Chemistry/Hematology[h] (D)	X	X***	X	X	X	X	X	X	X		X			X		
Fasting Coagulation Studies (PTT/INR)[i]	X	X***					X		X		X					

Assessment	Screening/ Base-line	Treatment Day 1	2 wks (± 2 Days)	4 wks (± 2 Days)	6 wks† (± 2 Days)	8 wks (± 2 Days)	12 wks (± 7 Days)	18 wks (± 7 Days)	24 wks (± 7 Days)	Every 4 wks there-after (± 7 Days)	Every 12 wks there-after (± 7 Days)	Every 24 wks there-after (± 7 Days)	Ann-ually (± 7 Days)	End of treatment (28 days after last dose)	Follow-Up	Study Completion
**Time point (days)**	**-28 to -1**	**0**	**14**	**28**	**42**	**56**	**84**	**126**	**168**							**Last**
**Visit no.**	**1**	**2**	**3**	**4**	**5**	**6**	**7**	**8**	**9**					**777 or 779**		**780**
(D)																
Fasting Serum Lipid Profile[j] (D)	X	X***					X		X		X					
Urinalysis[k] (D)	X	X***		X		X	X	X	X		X			X		
Prior concomitant medications[l] (D)	X															
Current concomitant medications[l] (D)	X	X	X	X	X	X	X	X	X		X			X	X	X
Adverse events[m] (D)	X	X	X	X	X	X	X	X	X		X			X		
Serious Adverse Events[m] (D)	X	X	X	X	X	X	X	X	X		X			X	X	X
Neuropsychological Assessments[n] (D)	X								X			X		X		
24 hour video EEG[o] (D)	X								X							
Seizure Severity Questionnaire[p] (D)	X								X							
IWRS Randomization[q] (D)		X														
Dispense Study Drug [r] (S)		X		X		X	X	X	X		X					

**Assessment**	**Screening/ Base-line**	**Treatment Day 1**	**2 wks (± 2 Days)**	**4 wks (± 2 Days)**	**6 wks**† **(± 2 Days)**	**8 wks (± 2 Days)**	**12 wks (± 7 Days)**	**18 wks (± 7 Days)**	**24 wks (± 7 Days)**	**Every 4 wks there-after (± 7 Days)**	**Every 12 wks there-after (± 7 Days)**	**Every 24 wks there-after (± 7 Days)**	**Ann-ually (± 7 Days)**	**End of treatment (28 days after last dose)**	**Follow-Up**	**Study Completion**
**Time point (days)**	**-28 to -1**	**0**	**14**	**28**	**42**	**56**	**84**	**126**	**168**							**Last**
**Visit no.**	**1**	**2**	**3**	**4**	**5**	**6**	**7**	**8**	**9**					**777 or 779**		**780**
Study Drug Compliance[s] (S)			X	X	X	X	X	X	X		X			X		
Biomarkers blood sampling[t] (D)	X			X			X		X		X *			X		
TSC1 and *TSC2* genetic analysis (D)	X															
PK blood sampling[u] (D)			X	X	X	X	X	X	X		X					
Digital Photographs of Skin lesions[v] (D)	X						X		X		X			X		
Physician's Global Assessment of Skin Lesions[w] (D)							X		X		X			X		
Tanner Staging/Development milestones[x] (D)	X												X			
Pregnancy History, Menstrual History and Monitoring [bb] (D)										X	X					
Parental height [cc]		To be collected at the next available study visit														
Full PK Profile (D)[y]		To be conducted at any one regular study visit under Amendment 5														

Assessment	Screening/ Baseline	Treatment Day 1	2 wks (± 2 Days)	4 wks (± 2 Days)	6 wks[†] (± 2 Days)	8 wks (± 2 Days)	12 wks (± 7 Days)	18 wks (± 7 Days)	24 wks (± 7 Days)	Every 4 wks there-after (± 7 Days)	Every 12 wks there-after (± 7 Days)	Every 24 wks there-after (± 7 Days)	Ann-ually (± 7 Days)	End of treatment (28 days after last dose)	Follow-Up	Study Completion
**Time point (days)**	**-28 to -1**	**0**	**14**	**28**	**42**	**56**	**84**	**126**	**168**							**Last**
**Visit no.**	**1**	**2**	**3**	**4**	**5**	**6**	**7**	**8**	**9**					**777 or 779**		**780**
HCV RNA-PCR[z]				X			X	X			X					
Pulmonary function tests[aa](D)			Only to be done if clinically indicated													
Chest CT[aa] (D)			Only to be done if clinically indicated													

* Biomarker samples will only be collected through week 48 (unless local or national regulations do not permit). An additional biomarker sample will be collected at the End of Treatment visit.

**MRI of the brain and CT or MRI of the kidneys will be completed for all patients at baseline, and MRI of the brain for all patients and CT/MRI of the kidney (for patients with Angiomyolipomata ≥ 1 cm in longest diameter) at 12, 24 and 48 weeks after the start of treatment, and annually thereafter. For each patient, the same imaging modality must be used throughout the trial.

***Screening safety laboratories (biochemistry, hematology, lipid panel, urinalysis, serum pregnancy (if applicable) and coagulation) only need to be repeated if they were collected more than 14 days prior to treatment Day 1.

∞ Only patients who meet the criteria specified in Section 4.1 for Hepatitis B and C testing will require these tests. Any patient who tests positive must be excluded with the exception of Hepatitis B antibody positives caused by prior vaccination.

[Ψ] Endocrine testing; testosterone, FSH, LH and estradiol (for females) will be collected annually until the patient's 10th birthday and then every 12 weeks thereafter..

α An MRI of the brain or MRI/CT of the kidney would be repeated at the End of Treatment visit if the patient has discontinued for reasons other than radiological progression and it has been more than 8 weeks since their most recent scan during the first year of treatment or more than 6 months since their most recent scan thereafter.

† For Visit 5 (6 week visit), patients for whom travel is difficult, who have tolerated study medication and have no adverse events may, at the discretion of the investigator, attend a local laboratory for blood draws (if such facility is available) and have a telephone consultation with the investigator rather than a clinic visit.

Visits 3, 4, 5 and 6 must be completed within 2 days of the scheduled visit. All other visits with the exception of Visit 2, must be completed within ± 7 days of scheduled visit, with the exception of the baseline visit which must be conducted no more than 28 days after the screening visit. All tests and procedures (i.e., MRIs, hematology

Assessment	Screening/ Base-line	Treatment Day 1	2 wks (± 2 Days)	4 wks (± 2 Days)	6 wks† (± 2 Days)	8 wks (± 2 Days)	12 wks (± 7 Days)	18 wks (± 7 Days)	24 wks (± 7 Days)	Every 4 wks there-after (± 7 Days)	Every 12 wks there-after (± 7 Days)	Every 24 wks there-after (± 7 Days)	Ann-ually (± 7 Days)	End of treatment (28 days after last dose)	Follow-Up	Study Completion
**Time point (days)**	**-28 to -1**	**0**	**14**	**28**	**42**	**56**	**84**	**126**	**168**							**Last**
**Visit no.**	**1**	**2**	**3**	**4**	**5**	**6**	**7**	**8**	**9**					**777 or 779**		**780**

labs, biochemistry labs) that occur within the allotted windows will not constitute a protocol deviation.

a. Patients should be registered through IWRS after informed consent is signed.

b. Serum Pregnancy test only to be performed in females of child-bearing potential at central lab within 14 days of randomization. A serum pregnancy test will be conducted locally at baseline and at end of treatment in all females of child-bearing potential. After Protocol Amendment # 6 implementation, a urine pregnancy test will be repeated every 4 weeks at patient's home according to study visit schedule until study drug is discontinued. **Note:** Urine pregnancy test will be conducted at clinical site in place of patient's home when a visit is scheduled. Results of at home urine pregnancy test will be recorded in patient diaries for source documentation only. .

c. Significant findings from physical examination will be noted on the Relevant Medical History or Adverse Events CRF pages. Physical exams should include Tanner Staging at the prescribed timepoints (at screening/baseline and annually) and evaluation for attainment of growth and development milestones (age at thelarche (females), age at adrenarche (males), date of menarche (females)).

d. Performance status should be assessed using the WHO Performance Scale for patients aged 13 years or older, and the Lansky Play Performance Status for patients aged from 0 to 12 years inclusive (at randomization). Patients assessed by Lansky Play Performance Status at screening should continue to be assessed with this tool throughout the trial, even after they have reached the age of 13 years (note that the tool has been designed for children up to the age of 18).

e. MRI of the brain should be performed at screening/baseline, at 12, 24 and 48 weeks after start of study treatment, and annually thereafter, unless observation of SEGA response warrants a confirmation approximately 12 weeks later (and no sooner than 8 weeks). For patients who respond at 12 weeks of treatment, the routine 24 week scan is sufficient to confirm response. MRI is not required at 18 weeks.

f. CT or MRI of the kidneys should be performed at screening/baseline for all patients. For all patients with at least one angiomyolipoma with longest diameter ≥ 1.0 cm at screening, CT/MRI of the kidneys should be repeated at 12, 24 and 48 weeks after start of study treatment, and annually thereafter, unless observation of response warrants a confirmation approximately 12 weeks later (and no sooner than 8 weeks later). CT/MRI of the kidneys is not required at 18 weeks. For each patient, the same imaging modality must be used throughout the trial.

g. ECG may be repeated at the investigator's discretion if there are signs or symptoms of cardiotoxicity. Significant findings will be noted on the Relevant Medical History or Adverse Events CRF pages.

Assessment	Screening/ Base-line	Treatment Day 1	2 wks (± 2 Days)	4 wks (± 2 Days)	6 wks† (± 2 Days)	8 wks (± 2 Days)	12 wks (± 7 Days)	18 wks (± 7 Days)	24 wks (± 7 Days)	Every 4 wks there-after (± 7 Days)	Every 12 wks there-after (± 7 Days)	Every 24 wks there-after (± 7 Days)	Ann-ually (± 7 Days)	End of treatment (28 days after last dose)	Follow-Up	Study Completion
**Time point (days)**	-28 to -1	0	14	28	42	56	84	126	168							Last
**Visit no.**	1	2	3	4	5	6	7	8	9					777 or 779		780

h. Fasting hematology must include: hemoglobin, hematocrit, platelets, red blood cell count (RBC) total white blood cell count (WBC) absolute & differential including neutrophils, lymphocytes, monocytes, eosinophils and basophils. Absolute Neutrophil Count (ANC) will be calculated by the laboratory. Fasting serum chemistry must include: total LDH, fasting glucose, sodium, magnesium, phosphate, potassium, chloride, bicarbonate, creatinine, BUN, albumin, total protein, SGOT (AST), SGPT (ALT), total bilirubin, alkaline phosphatase, uric acid, calcium.

i. Fasting Prothrombin time (PT) will be determined at screening and every 12 weeks while on study (at 12 and 24 weeks and every 12 weeks thereafter); it will be reported as international normalized ratio (INR). In addition, fibrinogen and partial thromboplastin (PTT) will be determined at screening and every 12 weeks while on study.

j. Fasting Serum lipid profile must include: total cholesterol, triglycerides, LDL, and HDL. Assessment should be repeated every 12 weeks.

k. Standard urinalysis dipstick assessment must include: pH, protein, glucose, blood, ketones, and leukocytes.

l. Medications taken within the 30 days prior to starting treatment and up to 84 days after last dose of study drug should be documented on the appropriate CRF.

m. AEs should be recorded on the Adverse Events CRF from the time of starting study treatment and up to 28 days after last dose (until the follow-up visit). All SAEs occurring within 28 days of study treatment discontinuation (until the follow-up visit), regardless of causality, should be captured on Adverse Events CRF. SAEs with suspected causality to study drug should be captured for an additional 8 weeks (56 days) after follow-up visit for a total of 12 weeks (84 days (12 weeks)) after treatment discontinuation.

n. One of the following assessments must be conducted: Wechsler Pre-School and Primary Scale of Intelligence, Wechsler Abbreviated Scale of Intelligence or the Vineland Adaptive Behavior Scale. The test that is administered will depend on the patient's age at randomization, the patient's cognitive/behavioral status, and whether the assessment is available in the patient's native language.

o. 24-hour video EEG to be conducted at screening/baseline and week 24 (or End of Treatment if the patient discontinues prior to week 24), and sent for an independent central review (screening/baseline EEG should only be performed once it has been confirmed that the patient is eligible to be randomized).

p. Seizure Severity Questionnaire to be filled out for patients being treated with antiepileptics at baseline, whenever available in the patient's native language.

q. Patients should be randomized through IWRS, after all eligibility criteria have been confirmed.

r. Study drug will be dispensed at the indicated visits. At each dispensing visit, site personnel will log into IWRS to obtain the patient's study drug assignment to last

Assessment	Screening/ Base-line	Treatment Day 1	2 wks (± 2 Days)	4 wks (± 2 Days)	6 wks† (± 2 Days)	8 wks (± 2 Days)	12 wks (± 7 Days)	18 wks (± 7 Days)	24 wks (± 7 Days)	Every 4 wks there-after (± 7 Days)	Every 12 wks there-after (± 7 Days)	Every 24 wks there-after (± 7 Days)	Ann-ually (± 7 Days)	End of treatment (28 days after last dose)	Follow-Up	Study Completion
Time point (days)	-28 to -1	0	14	28	42	56	84	126	168							Last
Visit no.	1	2	3	4	5	6	7	8	9					777 or 779		780

until the next dispensing visit including sufficient overage.

s. At each study visit, site personnel will review the patient's returned study drug, foil packs (used and unused) to ensure the patient is compliant

t. A biomarker blood sample will be collected (unless local or national regulations do not permit) at screening/baseline, at 4, 12, 24, 36, and 48 weeks, and at end of treatment. No additional biomarker samples will be collected.

u. Blood samples for trough RAD001 levels will be collected from all patients pre-dose at every visit starting at week 2 (Visit 3) and until discontinuation of study drug. In addition to a trough collection, a blood sample for C_{max} will be collected 2.0 hours (± 30 mins) after dosing at week 2 (Visit 3) and 1-2 weeks after any dose increase to a new level, or any decrease in an enzyme-inducing drug, or any increase in an enzyme-inhibiting drug.

v. For patients with skin lesions at screening/baseline: Skin lesions will be photographed using a digital camera and measured using a disposable ruler. Skin lesion photographs will be taken at baseline, 12 and 24 weeks after start of study treatment, every 12 weeks thereafter and End of treatment. Unless observation of response warrants a confirmation approximately 12 weeks later (and no sooner than 8 weeks).

w. A physician's Global Assessment evaluation of the patient's skin lesions will be conducted beginning at week 12 and every 12 weeks thereafter. Unless observation of response warrants a confirmation approximately 12 weeks later (and no sooner than 8 weeks). The assessment will be conducted according to Table 7-3.

x. Tanner Staging at the prescribed timepoints (at screening/baseline and annually) and evaluation for attainment of growth and development milestones (age at thelarche(females), age at adrenarche (males), date of menarche (females)).

y. For those patients who have consented to have the additional samples collected as part of Amendment 5 (predose, 0.5 hr, 1 hr, 2 hr, 5, hr and 24 hr), these would be collected at any ONE of the patient's regularly scheduled visit. The originally planned predose sample and the 2 hr post-dose sample, (if required) will not be collected on this visit day. Patients participating in this full PK profile assessment should follow the meal intake guidelines specified in Section 7.7.1 prior to and after receiving their everolimus dose on the PK collection visit day.

z. If an active patient tests positive to Hepatitis C antibody and does not meet the criteria for HCV flare according to Table 4-3, Hepatitis RNA-PCR should be tested according to the table above.

aa. If an investigator suspects a patient may be developing pneumonitis, investigations such as pulmonary function tests, CT chest and referral to a pulmonologist should be considered. For more detailed management advice see Table 6-3. Chest CT scan should be performed as clinically indicated.

Assessment	Screening/ Base-line	Treatment Day 1	2 wks (± 2 Days)	4 wks (± 2 Days)	6 wks† (± 2 Days)	8 wks (± 2 Days)	12 wks (± 7 Days)	18 wks (± 7 Days)	24 wks (± 7 Days)	Every 4 wks there-after (± 7 Days)	Every 12 wks there-after (± 7 Days)	Every 24 wks there-after (± 7 Days)	Ann-ually (± 7 Days)	End of treatment (28 days after last dose)	Follow-Up	Study Completion
**Time point (days)**	-28 to -1	0	14	28	42	56	84	126	168							Last
**Visit no.**	1	2	3	4	5	6	7	8	9					777 or 779		780

bb. Additional menstrual history (previous cases of amenorrhea or menstrual disorders, biological mother's age at menopause) and pregnancy history (Pregnancies, full-term gestations, abortions, live births, miscarriages) will be collected one time after implementation of protocol amendment 6. Following implementation of amendment 6, menstrual status will be collected monthly via patient diary. Then every 12 weeks during patient's scheduled visit, data from patient diary will be collected at clinical site for CRF. It is recommended that study coordinators contact patients monthly for the first 3 months to remind patients to document menstrual status in patient diary.

cc. Parental height will be used to estimate the patient height for all pediatric patients, to monitor growth and development.

Table 7-2 Open-label and extension phase visit evaluation schedule

NOTE: To be followed for patients who are initiating RAD001 for the first time

Assessment	Baseline	Treatment Day 1	2 weeks (± 2 Days)	4 weeks (± 2 Days)	6 weeks† (± 2 Days)	8 weeks (± 2 Days)	12 weeks (± 7 Days)	18 weeks (± 7 Days)	24 weeks (± 7 Days)	Every 4 weeks there-after (± 7 Days)	Every 12 weeks there after (± 7 Days)	Every 24 weeks there after (± 7 Days)	Annually (± 7 Days)	End of treat-ment (28 days after last dose)	Follow-Up	Study Completion
**Time point (days)**	-28 to -1	0	14	28	42	56	84	126	168							Last
**Visit no.**	101	102	103	104	105	106	107	108	109					778 or 779		780
Vital signs (D)	X	X		X		X	X	X	X		X			X		
Physical exam (including Neurological Exam)[a] (S)	X	X		X		X	X	X	X		X			X		
Sexual Maturation/Endocrine Assessments$^{\psi}$ (D)	X^{ψ}						X^{ψ}		X^{ψ}		X^{ψ}	X^{ψ}		X^{ψ}		
Performance status (WHO or Lansky)[b] (D)	X	X		X		X	X	X	X		X			X		
MRI of the Brain[c] (D)	X*						X		X		X*			Xα		
CT/MRI of the Kidney[d] (D)	X*						X		X		X*			Xα		
ECG[e] (D)	X													X		

Assessment	Baseline	Treatment Day 1	2 weeks (± 2 Days)	4 weeks (± 2 Days)	6 weeks[†] (± 2 Days)	8 weeks (± 2 Days)	12 weeks (± 7 Days)	18 weeks (± 7 Days)	24 weeks (± 7 Days)	Every 4 weeks there-after (± 7 Days)	Every 12 weeks there after (± 7 Days)	Every 24 weeks there after (± 7 Days)	Annually (± 7 Days)	End of treat-ment (28 days after last dose)	Follow-Up	Study Completion
**Time point (days)**	-28 to -1	0	14	28	42	56	84	126	168							Last
**Visit no.**	101	102	103	104	105	106	107	108	109					778 or 779		780
Fasting serum chemistry/ Hematology[f] (D)	X		X	X	X	X	X	X	X		X			X		
Fasting Coagulation Studies (PTT/INR)[g] (D)	X						X		X		X					
Fasting Serum Lipid Profile[h] (D)	X						X		X		X					
Urinalysis[i](D)	X			X		X	X	X	X		X			X		
Prior concomitant medications[j] (D)	X															
Current concomitant medications[j] (D)	X	X	X	X	X	X	X	X	X		X			X	X	X
Adverse Events[k] (D)	X	X	X	X	X	X	X	X	X		X			X	X	X
Serious	X	X	X	X	X	X	X	X	X		X			X	X	X

Assessment	Baseline	Treatment Day 1	2 weeks (± 2 Days)	4 weeks (± 2 Days)	6 weeks † (± 2 Days)	8 weeks (± 2 Days)	12 weeks (± 7 Days)	18 weeks (± 7 Days)	24 weeks (± 7 Days)	Every 4 weeks there-after (± 7 Days)	Every 12 weeks there after (± 7 Days)	Every 24 weeks there after (± 7 Days)	Annually (± 7 Days)	End of treat-ment (28 days after last dose)	Follow-Up	Study Completion
**Time point (days)**	-28 to -1	0	14	28	42	56	84	126	168							Last
**Visit no.**	101	102	103	104	105	106	107	108	109					778 or 779		780
Adverse events[k] (D)																
Neuropsychological [l]Assessments[m] (D)	X								X			X		X		
24 hour video EEG[m] (D)	X*								X							
Seizure Severity Questionnaire[n] (D)	X								X							
Dispense Study Drug [o] (S)		X		X		X	X	X	X		X					
Study Drug Compliance[p] (S)			X	X	X	X	X	X	X		X			X		
PK blood sampling[q] (D)			X	X	X	X	X	X	X		X			X		
Digital Photographs of Skin lesions[r]	X						X		X		X			X		

Assessment	Baseline	Treatment Day 1	2 weeks (± 2 Days)	4 weeks (± 2 Days)	6 weeks[†] (± 2 Days)	8 weeks (± 2 Days)	12 weeks (± 7 Days)	18 weeks (± 7 Days)	24 weeks (± 7 Days)	Every 4 weeks there-after (± 7 Days)	Every 12 weeks there after (± 7 Days)	Every 24 weeks there after (± 7 Days)	Annually (± 7 Days)	End of treat-ment (28 days after last dose)	Follow-Up	Study Completion
**Time point (days)**	-28 to -1	0	14	28	42	56	84	126	168							Last
**Visit no.**	101	102	103	104	105	106	107	108	109					778 or 779		780
(D)																
Physician's Global Assessment of Skin Lesions[s] (D)							X		X		X			X		
Tanner Staging/Development milestones[t] (D)	X												X			
Urine pregnancy test[x] (D)		X		X		X	X		X	X	X					
Serum Pregnancy Test[x] (D)														X		
Pregnancy History, Menstrual History and Monitoring[y] (D)										X	X					
Parental height[z]		To be collected at the next available study visit														

Assessment	Baseline	Treatment Day 1	2 weeks (± 2 Days)	4 weeks (± 2 Days)	6 weeks † (± 2 Days)	8 weeks (± 2 Days)	12 weeks (± 7 Days)	18 weeks (± 7 Days)	24 weeks (± 7 Days)	Every 4 weeks there-after (± 7 Days)	Every 12 weeks there after (± 7 Days)	Every 24 weeks there after (± 7 Days)	Annually (± 7 Days)	End of treat-ment (28 days after last dose)	Follow-Up	Study Completion
**Time point (days)**	-28 to -1	0	14	28	42	56	84	126	168							Last
**Visit no.**	101	102	103	104	105	106	107	108	109					778 or 779		780
Full PK Profile[u] (D)		To be conducted at any one regular study visit under Amendment 5														
HCV RNA-PCR[v]				X			X	X			X					
Pulmonary function tests[w] (D)			Only to be done if clinically indicated													
Chest CT[w] (D)			Only to be done if clinically indicated													

*The most recent MRI of the brain and CT/MRI of the kidney (if followed) from the blinded phase of the study will be used as the baseline (if completed within 12 weeks of initiating RAD001). Additional MRIs of the brain will be conducted at 12, 24 and 48 weeks after start of open-label RAD001, and annually thereafter. CT/MRI of the kidneys would only be conducted if the patient had angiomyolipoma with longest diameter ≥ 1.0 cm at screening during the blinded phase. For each patient, the same imaging modality must be used throughout the trial.

**All Open label baseline laboratories must be collected with 14 days of Open Label Treatment day 1.

† For visit 105 (6 week visit), patients for whom travel is difficult, who have tolerated study medication and have no adverse events may, at the discretion of the investigator, attend a local laboratory for blood draws (if such facility is available) and have a telephone consultation with the investigator rather than a clinic visit. Visits 103, 104, 105 and 106 must be completed within 2 days of the scheduled visit. All other visits with the exception of Visit 102, must be completed within ± 7 days of scheduled visit, with the exception of the baseline visit which must be conducted no more than 28 days after the blinded End of Treatment Visit. All tests and procedures (i.e., MRIs, hematology labs, biochemistry labs) that occur within the allotted time before or after the scheduled visit will not constitute a protocol deviation.

α An MRI of the brain or MRI/CT of the kidney would be repeated at the End of Treatment visit if the patient has discontinued for reasons other than radiological progression and it has been more than 8 weeks since their most recent scan during the first year of treatment or more than 6 months since their most recent scan thereafter.

[Ψ] Endocrine testing (testosterone, FSH, LH and estradiol (for females) will be completed annually until the patient's 10th birthday and then every 12 weeks thereafter.. In addition, Tanner Staging will continue annually as part of the physical exam.

[a] Significant changes from last Physical Examination conducted in the blinded phase will be noted on the Adverse Events pages. Physical exams should include.

Assessment	Baseline	Treatment Day 1	2 weeks (± 2 Days)	4 weeks (± 2 Days)	6 weeks † (± 2 Days)	8 weeks (± 2 Days)	12 weeks (± 7 Days)	18 weeks (± 7 Days)	24 weeks (± 7 Days)	Every 4 weeks there-after (± 7 Days)	Every 12 weeks there after (± 7 Days)	Every 24 weeks there after (± 7 Days)	Annually (± 7 Days)	End of treat-ment (28 days after last dose)	Follow-Up	Study Completion
**Time point (days)**	-28 to -1	0	14	28	42	56	84	126	168							Last
**Visit no.**	101	102	103	104	105	106	107	108	109					778 or 779		780

b. Performance status should be assessed using the WHO Performance Status for patients aged 13 years or older, and the Lansky Play Performance Status for patients aged from 0 to 12 years inclusive (at randomization). Patients assessed by Lansky Play Performance Status at baseline during the blinded phase should continue to be assessed with this tool throughout the trial, even after they have reached the age of 13 years (note that the tool has been designed for children up to the age of 18).

c. MRI of the brain should be performed at 12, 24 and 48 weeks after start of open label treatment, and annually thereafter unless observation of SEGA response warrants a confirmation approximately 12 weeks later (and no sooner than 8 weeks). For patients who respond at 12 weeks of treatment, the routine 24 week scan is sufficient to confirm response. MRI is not required at 18 weeks.

d. For all patients with at least one angiomyolipoma with longest diameter ≥ 1.0 cm at screening of the blinded phase, CT/MRI of the kidneys should be repeated at 12, 24 and 48 weeks after start of open label treatment, and annually thereafter unless observation of response warrants a confirmation approximately 12 weeks later (and no sooner than 8 weeks). CT/MRI of the kidneys is not required at 18 weeks. For each patient, the same imaging modality must be used throughout the trial

e. ECG may be repeated at the investigator's discretion if there are signs or symptoms of cardiotoxicity. Significant findings will be noted in the Relevant Medical History or Adverse Events CRF pages.

f. Fasting hematology must include: hemoglobin, hematocrit, platelets, red blood cell count (RBC) total white blood cell count (WBC) absolute & differential including neutrophils, lymphocytes, monocytes, eosinophils and basophils. Absolute Neutrophil Count (ANC) will be calculated by the laboratory. Fasting serum chemistry must include: total LDH, fasting glucose, sodium, magnesium, phosphate, potassium, chloride, bicarbonate, creatinine, BUN, albumin, total protein, SGOT (AST), SGPT (ALT), total bilirubin, alkaline phosphatase, uric acid, calcium.

g. Fasting prothrombin time (PT) will be determined at screening and every 12 weeks while on study (at 12 and 24 weeks and every 12 weeks thereafter); it will be reported as international normalized ratio (INR). In addition, fibrinogen and partial thromboplastin (PTT) will be determined at screening and every 12 weeks while on study.

h. Fasting serum Lipid profile must include: total cholesterol, triglycerides, LDL, and HDL. Assessment should be repeated every 12 weeks.

i. Standard urinalysis dipstick assessment must include: pH, protein, glucose, blood, ketones, and leukocytes.

j. Medications taken up to 84 days (12 weeks) after last dose of study drug should be documented on the appropriate CRF.

k. AEs will continue to be recorded on the Adverse Events CRF up to 28 days after last dose (until the follow-up visit). All SAEs occurring within 28 days of study treatment discontinuation (until the follow-up visit), regardless of causality, should be captured on Adverse Events CRF. SAEs with suspected causality to study drug should be

Assessment	Baseline	Treatment Day 1	2 weeks (± 2 Days)	4 weeks (± 2 Days)	6 weeks † (± 2 Days)	8 weeks (± 2 Days)	12 weeks (± 7 Days)	18 weeks (± 7 Days)	24 weeks (± 7 Days)	Every 4 weeks there-after (± 7 Days)	Every 12 weeks there after (± 7 Days)	Every 24 weeks there after (± 7 Days)	Annually (± 7 Days)	End of treat-ment (28 days after last dose)	Follow-Up	Study Completion
**Time point (days)**	-28 to -1	0	14	28	42	56	84	126	168							Last
**Visit no.**	101	102	103	104	105	106	107	108	109					778 or 779		780

captured for an additional 8 weeks (56 days) after follow-up visit for a total of 12 weeks (84 days) after treatment discontinuation.

l. One of the following assessments must be conducted: Wechsler Pre-School and Primary Scale of Intelligence, Wechsler Abbreviated Scale of Intelligence or the Vineland Adaptive Behavior Scale. The test that is administered will depend on the patient's age at randomization, the patient's cognitive/behavioral status, and whether the assessment is available in the patient's native language.

m. 24-hour video EEG to be conducted at baseline (if more than 12 weeks has passed since the patient's previous video EEG) and week 24 (or End of Treatment if the patient discontinues prior to week 24), and sent for an independent central review.

n. Seizure Severity Questionnaire to be filled out for patients being treated with antiepileptics at baseline, whenever available in the patient's native language.

o. Study drug will be dispensed at the indicated visits. At each dispensing visit, site personnel will log into IWRS to obtain the patient's drug assignment to last until the next dispensing visit including sufficient overage.

p. At each study visit, site personnel will review the patient's returned study drug, foil packs (used and unused) to ensure the patient is compliant

q. Blood samples for trough RAD001 levels will be collected from all patients pre-dose at every visit starting at week 2 (Visit 103) and until discontinuation of study drug. In addition to a trough sample collection, a blood sample for C_{max} will be collected 2.0 hours (± 30 mins) after dosing at week 2 (Visit 103) and 1-2 weeks after any dose increase.

r. For patients whose skin lesions were followed during the blinded phase, Skin lesions will be photographed using a digital camera and measured using a disposable ruler. Skin lesion photographs will be taken at baseline, 12 and 24 weeks after start of open label treatment, every 12 weeks thereafter and at end of treatment**.** Unless observation of response warrants a confirmation approximately 12 weeks later (and no sooner than 8 weeks later).

s. A physician's Global Assessment evaluation of the patient's skin lesions will be conducted beginning at week 12 and every 12 weeks thereafter. Unless observation of response warrants a confirmation approximately 12 weeks later (and no sooner than 8 weeks later). The assessment will be conducted according to Table 7-3.

t Tanner Staging at the prescribed timepoints and evaluation for the attainment of growth and development milestones (age at thelarche (females), age at adrenarche (males), date of menarche (females)).

u For those patients who have consented to have the additional samples collected as part of Amendment 5 (predose, 0.5 hr, 1 hr, 2 hr, 5, hr and 24 hr), these would be collected at any ONE of the patient's regularly scheduled visit. The originally planned predose sample and the 2 hr post-dose sample, (if required) will not be collected on this visit day.

Assessment	Baseline	Treatment Day 1	2 weeks (± 2 Days)	4 weeks (± 2 Days)	6 weeks † (± 2 Days)	8 weeks (± 2 Days)	12 weeks (± 7 Days)	18 weeks (± 7 Days)	24 weeks (± 7 Days)	Every 4 weeks there-after (± 7 Days)	Every 12 weeks there after (± 7 Days)	Every 24 weeks there after (± 7 Days)	Annually (± 7 Days)	End of treat-ment (28 days after last dose)	Follow-Up	Study Completion
**Time point (days)**	-28 to -1	0	14	28	42	56	84	126	168							Last
**Visit no.**	101	102	103	104	105	106	107	108	109					778 or 779		780

[v] If an active patient tests positive to Hepatitis C antibody and does not meet the criteria for HCV flare according to Table 4-3, Hepatitis RNA-PCR should be tested according to the table above.

[w] If an investigator suspects a patient may be developing pneumonitis investigations such as pulmonary function tests, CT chest and referral to a pulmonologist should be considered. For more detailed management advice see Table 6-3. Chest CT scan should be performed as clinically indicated.

[x] A urine pregnancy test will be conducted locally at baseline in all females of child-bearing potential. After Protocol Amendment # 6 implementation, a urine pregnancy test will be repeated every 4 weeks at patient's home according to study visit schedule after study drug is discontinued. **Note:** Urine pregnancy test will be conducted at clinical site in place of patient's home when a visit is scheduled. A serum pregnancy test will be conducted at end of treatment. Results of at home urine pregnancy test will be recorded in patient diaries for source documentation only.

[y] Additional menstrual history (previous cases of amenorrhea or menstrual disorders, biological mother's age at menopause) and pregnancy history (Pregnancies, full-term gestations, abortions, live births, miscarriages) will be collected one time after implementation of protocol amendment 6. Following implementation of amendment 6, menstrual status will be collected monthly via patient diary. Then every 12 weeks during patient's scheduled visit, data from patient diary will be collected at clinical site for CRF. It is recommended that study coordinators contact patients monthly for the first 3 months to remind patients to document menstrual status in patient diary.

[z] Parental height will be used to estimate the patient height for all pediatric patients, to monitor growth and development.

7.1 Information to be collected on screening failures

Patients who complete the informed consent process and do **not** meet all entry criteria and therefore who do not receive RAD001 or matching placebo will be considered screen failures. Screen failures should be entered into the Screen Failure Log. The screening failure data will be entered in the clinical database.

7.2 Inclusion/exclusion criteria

Information regarding eligibility criteria will be collected on the Inclusion/Exclusion CRF. Patients who do not meet all entry criteria should not be entered into the study.

7.3 Patient demographics/other baseline assessments

Data will be collected on patient characteristics including demographic information (age, sex, race, weight, height) and other background or relevant medical history (disease history, family history of disease, prior anti-SEGA therapies, hepatitis screening (for patients meeting criteria outlined in Section 4.1) and HIV history), and any other assessments that are done for the purpose of determining eligibility for inclusion in the study [i.e., performance status (WHO or Lansky, depending on age of patient), complete physical examination (including Tanner Staging), vital signs, hematology, blood chemistries including coagulation studies and a serum lipid profile, urinalysis, pregnancy test for women of child-bearing potential, MRI, ECG]. For all patients, all available prebaseline height and weight data should be collected in order to adequately represent the patient's rate of growth prior to starting the study.

Medical history will include family history of disease. Serum pregnancy test is required at screening/baseline to be followed by a urine pregnancy test on Treatment day 1 (prior to dosing) for eligibility. Urine pregnancy testing will then be repeated every 4 weeks until study drug discontinuation, and a serum pregnancy test will be repeated at end of study visit.

Parental height

The height for the biological parents will be collected at the next available visit. Parental height will be used to evaluate the growth and development of the pediatric patient population with TSC in this study,

7.3.1 Baseline assessments of SEGAs and angiomyolipomata

An MRI assessment of the brain will be performed for all patients at baseline to identify all SEGA lesions, and a baseline CT/MRI of the kidney will be performed to identify any angiomyolipomata with longest diameter ≥ 1.0 cm, respectively. For information regarding the scan acquisition protocol, please refer to the Independent Review Charter. These images will be sent for an independent central radiology review within 2 days of the scan for estimation of volume at each time point as specified in the Independent Review Charter.

7.3.2 Special laboratory tests

7.3.2.1 Pregnancy test

Women of child-bearing potential, defined as all women physiologically capable of becoming pregnant, must use highly effective contraception during the study and for 8 weeks after stopping treatment. Highly effective contraception is defined as either:

- Total abstinence: When this is in line with the preferred and usual lifestyle of the subject. [Periodic abstinence (e.g., calendar, ovulation, symptothermal, post-ovulation methods) and withdrawal are not acceptable methods of contraception].
- Sterilization: Have had surgical bilateral oophorectomy (with or without hysterectomy) or tubal ligation at least six weeks before taking study treatment. In case of oophorectomy alone, only when the reproductive status of the woman has been confirmed by follow up hormone level assessment.
- Male partner sterilization (with the appropriate post-vasectomy documentation of the absence of sperm in the ejaculate). [for female subjects on the study, the vasectomised male partner should be the sole partner for that subject].
- Use of a combination of any two of the following (a+b or a+c or b+c):
    a. Use of oral, injected or implanted hormonal methods of contraception
    b. Placement of an intrauterine device (IUD) or intrauterine system (IUS)
    c. Barrier methods of contraception: Condom or Occlusive cap (diaphragm or cervical/vault caps) with spermicidal foam/gel/film/cream/vaginal suppository

In case of use of oral contraception women should have been stabile on the same pill for a minimum of 3 months before taking study treatment.

Sexually active males must use a condom during intercourse while taking the drug and for 8 weeks after stopping treatment and should not father a child in this period.

A condom is required to be used also by vasectomised men (also during intercourse with a male partner) in order to prevent delivery of the drug via seminal fluid.

Female partners of male patients enrolled in the study must be advised to also use one of the following contraception methods: Use of (1) oral, injected or implanted hormonal methods of contraception, or (2) intrauterine device (IUD) or intrauterine system (IUS), or (3) prior male/female sterilization.

A serum pregnancy test must be completed within 14 days of randomization and a urine pregnancy test follow-up completed prior to randomization on Treatment Day 1. Female patients who are able to become pregnant will have a repeat urine pregnancy test every 4 weeks while receiving study drug until study drug discontinuation. Pregnancy testing is required at screening and monthly until the end of the study. Serum pregnancy testing should be performed at screening and at the end of the study. Urine pregnancy testing will be performed every 4 weeks at the patient's home, or at clinical site when a visit is scheduled until study drug is discontinued. Urine pregnancy test will be conducted at clinical site in place of patient's home when a visit is scheduled.

Patient should be instructed to inform site of a positive urine pregnancy result. Repeat serum pregnancy testing will be performed for confirmation of a positive urine pregnancy test.

7.3.2.2 HBV testing

Prior to randomization, the categories of patients listed in Section 4.1 should be tested for hepatitis B serologic markers and viral load: HBV-DNA HBsAg, HBc Ab, and HBs Ab. Patients with a positive result must be excluded from the study unless the result was caused by prior Hepatitis B vaccinations**.**

For patients who are active prior to the approval of Amendment 2, the above screening hepatitis B tests should be completed at the patient's next study visit. Should the patient test positive, the investigator should follow the guidelines provided in Table 4-1 and Table 4-2.

7.3.2.3 HCV testing

Patients with hepatitis C risk factors and additional patients at the discretion of the investigator should be tested for HCV RNA-PCR test at baseline. For a list of hepatitis C risk factors, refer to Section 4.1. Patients with a positive result must be excluded from the study.

For patients who are active prior to the approval of Amendment 2, the above screening hepatitis C tests should be completed at the patient's next study visit. If the patient tests positive for Hepatitis B, the investigator should follow the guidelines according to Table 4-1 and Table 4-2. Please refer to Table 7-1 and Table 7-2 for HCV RNA-PCR monitoring schedule for those patients with positive HCV RNA-PCR baseline tests who do not meet the HCV flare criteria outlined in Table 4-3. If the patient tests positive for hepatitis C, and the criteria for HCV flare according to Table 4-3 are observed, trial therapy should be discontinued and further treatment is up to the investigators discretion.

7.3.2.4 Endocrine Testing

A blood sample for analysis of total testosterone, FSH and LH and estradiol (females) will be collected during screening and annually until the patient's 10th birthday and then every 12 weeks thereafter for endocrine assessment. In the event that amenorrhea is reported in between scheduled assessments, hormone evaluations should be completed at that time.

For patients who are active prior to the approval of Amendment 4 the above screening should be completed at the next scheduled visit, if it had not been collected previously (considered baseline) at the intervals appropriate for their age as indicated above.

7.4 Treatments

Patients will start study treatment at Visit 2 (Treatment Day 1) and continue to be treated per protocol until documentation of SEGA progression, unacceptable toxicity, withdrawal of consent or investigator decision to stop the study. However, study treatment may prematurely be discontinued for other reasons as well. Please refer to Section 6.7.4

Compliance will be assessed by the investigator and/or study personnel at each visit using pill counts and information provided by the caregiver. This information should be captured in the source document at each visit.

7.5 Efficacy

7.5.1 Radiological evaluation

For instruction regarding baseline assessments of SEGA lesions, please refer to Table 7-1 and Section 7.3.1

The same method of assessment and the same technique should be used to characterize each identified and reported SEGA lesion at baseline, 12, 24 and 48 weeks after start of study treatment, and annually thereafter.

Each center must have a designated radiologist or other physician who is responsible for the interpretation of multiphase MRI. The same radiologist/physician should perform the evaluation for the entire duration of the study. All radiology evaluations will be performed initially by the local radiologist, but designation of response and progression will be based only on the evaluations made by the Independent Central Radiology Review. Following receipt of each MRI, the Central Radiology Review will be completed and the results will be communicated back to the participating center within 3 weeks.

If an initial observation of response is made, a confirmation scan should be obtained approximately 12 weeks after the initial observation (and no sooner than 8 weeks after).

All patients being discontinued from the study for SEGA progression must have their progression documented using the criteria specified in Section 7.5.2 In particular, a discontinuation reason of "SEGA progression" will not be sufficient to establish that SEGA progression actually occurred.

7.5.2 SEGA response evaluation

7.5.3 SEGA

SEGA response and progression evaluation will be performed according to the criteria outlined below.

Screening/baseline requirement:

All measurable SEGA lesions should be identified and recorded at baseline, and all patients should have at least one measurable target SEGA lesion with longest diameter ≥ 1.0 cm, as confirmed by multiphase MRI. Baseline evaluations should be performed as close as possible to the beginning of treatment and never more than 28 days before the beginning of treatment.

SEGA response assessment:

SEGA response will be defined as a reduction in SEGA volume of at least 50% relative to baseline, where SEGA volume is the sum of the volumes of all target SEGA lesions identified at baseline, and confirmed with a second scan performed approximately 12 weeks later (and no sooner than 8 weeks later). For patients who respond at 12 weeks of treatment, the routine 24 week scan is sufficient to confirm response. In addition, SEGA response requires that the non-target SEGA lesions have not unequivocally worsened, that no new SEGA lesions (≥ 1 cm in longest diameter) are identified, and the absence of new or worsening hydrocephalus

[defined by central radiological assessment of ventricular configuration changes, ventricular cap signs (periventricular edema) and qualitative assessment of CSF flow dynamics].

SEGA progression will be defined as either (1) an increase from nadir of 25% or more in SEGA volume to a value greater than baseline SEGA volume (where SEGA volume is the sum of the volumes of all target SEGA lesions identified at baseline and where nadir is the lowest SEGA volume achieved by the patient previously in the trial (including baseline)), or (2) the unequivocal worsening of non-target SEGA lesions, or (3) the appearance of a new SEGA lesion ≥ 1.0 cm in longest diameter, or (4) new or worsening hydrocephalus [defined by central radiological assessment of ventricular configuration changes, ventricular cap signs (periventricular edema) and qualitative assessment of CSF flow dynamics].

Note: In some instances disease that is measurable as a target lesion at baseline and appears to be one mass can split to become two or more smaller sub-lesions. When this occurs the image review system allows the reviewer to comment that the lesion has split, and to identify the separate sub-lesions as unique and non-overlapping so that the volume of each sub-lesion can be determined. The combined volumes of the sub-lesions will be reported as the volume of the lesion that has split, and only this lesion volume will be reported to the sites; linear measurements of the lesion that has split will not be reported. The individual split lesions will not be considered as new lesions, and will not automatically trigger a SEGA progression.

Conversely, it is also possible that two or more lesions which were distinctly separate at baseline become confluent at subsequent visits. When this occurs the image review system allows the reviewer to comment on which lesions have joined together, and to capture the required linear and volumetric measurements of each lesion that has joined to form the confluent mass. The overall dimensions and volume of the confluent mass will not be reported.

7.5.4 Angiomyolipoma response evaluation

Angiomyolipoma response and progression evaluation will be performed according to the criteria outlined below.

Screening/baseline requirement:

At baseline, all measurable angiomyolipomata with longest diameter ≥ 1.0 cm should be identified from each kidney; only patients with at least one angiomyolipoma ≥ 1.0 cm in longest diameter will be followed for angiomyolipoma response during the trial. Baseline evaluations should be performed as close as possible to the beginning of treatment and never more than 28 days before the beginning of treatment. For each patient, the same imaging modality must be used throughout the trial.

Target angiomyolipomata:

Up to five of the largest measurable lesions on each kidney seen at baseline, where measurable means at least 1.0 cm in longest diameter, should be identified as target angiomyolipomata. The volume of these lesions will be measured at each CT/MRI assessment of the kidney during the trial. The same imaging modality must be used throughout the trial. Angiomyolipoma volume is defined as the sum of the volumes of the individual target angiomyolipomata, and it is angiomyolipoma volume that is used directly in the definition of angiomyolipoma response and angiomyolipoma progression.

Kidney volume:

All other angiomyolipomata (i.e., angiomyolipomata other than the target angiomyolipomata defined above) present at baseline are non-target angiomyolipomata. In some cases there may be many non-target angiomyolipomata (e.g., >20), including non-measurable lesions (i.e., with longest diameter < 1.0 cm). Instead of attempting to individually assess each non-target angiomyolipoma during the trial, the volume of each kidney will be measured. Increases in the volume of either kidney will then be taken as evidence of worsening angiomyolipoma. This is expected to be particularly useful when target angiomyolipomata are relatively stable, but non-target angiomyolipomata are clearly progressing.

Angiomyolipoma response assessment:

Angiomyolipoma response will be defined as a reduction in angiomyolipoma volume of at least 50% relative to baseline, where angiomyolipoma volume is the sum of the volumes of all target angiomyolipomata identified at baseline, and confirmed with a second scan performed approximately 12 weeks later (and no sooner than 8 weeks later). In addition, angiomyolipoma response requires that no new angiomyolipomata ≥ 1.0 cm in longest diameter are identified, that neither kidney increases in volume by more than 20% from nadir (where nadir is the lowest kidney volume obtained for the patient, separately for each kidney, previously in the trial [including baseline]), and that the patient does not have any angiomyolipoma-related bleeding of grade ≥2 (as defined by NCI CTCAE, version 3.0).

Angiomyolipoma progression will be defined as either (1) an increase from nadir of 25% or more in angiomyolipoma volume to a value greater than baseline angiomyolipoma volume (where angiomyolipoma volume is the sum of the volumes of all target angiomyolipomata identified at baseline and where nadir is the lowest angiomyolipoma volume achieved by the patient previously in the trial (including baseline)), or (2) the appearance of a new angiomyolipoma ≥ 1.0 cm in longest diameter, or (3) an increase from nadir of 20% or more in the volume of either kidney to a value greater than baseline, where nadir is the lowest kidney volume obtained for the patient, separately for each kidney, previously in the trial (including baseline), or (4) angiomyolipoma-related bleeding of grade ≥2 as defined by NCI CTCAE, version 3.0.

In the unlikely event that a patient meets the criteria for angiomyolipoma progression and does not meet the criteria for SEGA progression, it will be up to the investigator to determine if the patient should remain on study or be discontinued. If a patient is discontinued due to meeting the criteria for angiomyolipoma progression and does not simultaneously meet the

criteria for SEGA progression, he/she is not eligible to be enrolled into the open-label phase of the study.

7.5.5 24-hour video EEG

The 24-hour video EEG will be performed for all patients at screening/baseline prior to randomization (but after confirmation that the patient is eligible to be randomized) and will be repeated after 24 weeks of treatment (or at end of treatment if patient discontinues treatment before 24 weeks). The video EEG recordings will be sent to a Central Reader for interpretation and recording of seizure frequency/type. Seizure frequency per 24 hours is defined as the number of seizures in the EEG divided by the number of hours in the EEG, multiplied by 24. The types of seizures that will be documented in this study include Complex Partial Seizures (including localization), Generalized Tonic Clonic Seizures (partial and generalized onset), Atonic / Tonic Seizures, Atypical Absence Seizures, Absence Seizures, Epileptic Spasms and Myoclonic Seizures. The Central Reader, who is an independent pediatric neurologist not involved with the conduct of the study, will be blinded to the patient’s treatment. The data provided by the Central Reader will be recorded and used in the efficacy analysis. Video EEG seizures should also be identified locally and recorded in the patient’s source documents. Guidelines for recording and shipping of the EEGs can be found in the EEG Patient Scanning Guide provided by the central reader.

7.5.6 Skin lesion

Skin lesions resulting from TSC include hypomelanotic macules, the shagreen patch, periungual or subungual fibromas, facial angiofibromas and/or forehead plaques. The types and locations of these skin lesions that are identified at screening/baseline should be recorded on the Tuberous Sclerosis Diagnosis CRF pages and followed throughout the study. Descriptions of each are given below.

Hypomelanotic macules are flat areas of skin that appear lighter than the surrounding skin. They can be any size or shape or may be the classic “ash-leaf” shape. Skin cells in this area of the skin contain less pigment, so the area appears lighter than the surrounding skin.

The shagreen patch is a patch of skin that is similar in color to surrounding skin, but may be tough and dimpled like an orange peel. The shagreen patch is usually found on the lower back and nape of the neck, but they may also be seen on other parts of the body.

Periungual or subungual fibromas are small fibrous growths that appear around the fingernails or toenails and are usually not seen until adult life.

Facial angiofibromas are benign tumors of the face that often appear across the cheeks and nose and on the chin. They are initially small reddish spots or bumps that may increase in size with age.

Lastly, a forehead plaque is similar to the angiofibroma but is found on the forehead and scalp. These flesh colored plaques are soft or compressible of doughy to hard lesions.

Digital photographs of all skin lesions will be taken every 12 weeks throughout the study.

7.5.6.1 Skin lesion response evaluation

7.5.6.1.1 Physician's Global Assessment of Clinical Condition (PGA)

The Physician's Global Assessment of Clinical Condition (PGA) is a 7-point grading scale that allows the investigator to evaluate the overall extent of improvement or worsening of the patient's skin disease as compared to baseline (see Table 7-3). This scale has been previously used in assessing skin lesions in other phase III trials (Duvic et al 2001; Heald et al 2003). Whenever possible, the same investigator should perform all skin evaluations on a patient in order to avoid inter-assessor variability. This assessment is designed to consider skin lesions as a whole. Responses must be confirmed by at least two assessments separated in time by approximately 12 weeks (and no less than 8 weeks). A complete clinical response (CCR) requires a grading of 0 indicating the absence of disease (histological confirmation is not required). Grades 1, 2, and 3 constitute partial response, indicating improvement of at least 50 percent, but less than 100 percent improvement.

Table 7-3 Physician's Global Assessment of Clinical Condition (PGA)

Grade	Description	Response
0 Completely clear	No evidence of disease; 100% improvement	CCR
1 Almost clear	Very significant clearance (≥90%-<100%); only traces of disease remains	PR
2 Marked improvement	Significant improvement (≥75%-<90%); some evidence of disease remains	PR
3 Moderate improvement	Intermediate between slight and marked improvement; (>50 %-< 75%)	PR
4 Slight improvement	Some improvement (>25%-<50%); however, significant evidence of disease remains	SD
5 No change	Disease has not changed from baseline condition (+<25%)	SD
6 Worse	Disease is worse than at baseline evaluation by >25% or more	PD

CCR, Clinical complete response; PR, Partial response; SD, Stable disease; PD, Progressive disease.

7.5.6.2 Digital photographs

Digital photographs of all skin lesions should be taken at baseline, using a high resolution digital camera (≥3 megapixels). The photographs will be repeated every 12 weeks after the start of study treatment. If a patient demonstrates a CCR or PR evaluated by PGA assessment, further photographs should be taken approximately 12 weeks later to confirm the response (and no sooner than 8 weeks later). These digital photographs will be used solely to document the response of these skin lesions to study treatment and should be sent to the central review facility for archiving.

7.6 Safety

Safety assessments will consist of monitoring and recording all AEs, including SAEs, the regular monitoring of hematology and blood chemistry, regular monitoring of vital signs and physical condition.

These assessments should be performed within the prescribed window of the scheduled day of assessment (Table 7-1 and Table 7-2) except for AEs and concomitant medications that will be evaluated and recorded continuously throughout the study.

7.6.1 Adverse events

An adverse event for the purposes of this protocol is the appearance of (or worsening of any pre-existing) undesirable sign(s), symptom(s), or medical condition(s) occurring after signing the informed consent even if the event is not considered to be related to the study drug(s).

Please refer to Section 6.1 for the protocol-specific definitions of study drug and study treatment.

Adverse events (but not serious adverse events) occurring before starting study treatment but after signing the informed consent form are recorded on the Medical History/Current Medical Conditions Electronic CRF. Abnormal laboratory values or test results constitute adverse events only if they induce clinical signs or symptoms, or are considered clinically significant or require therapy (e.g., any hematological abnormality that requires transfusion or cytokine treatment), and should be recorded on the Adverse Events CRF under the signs, symptoms or diagnosis associated with them. In addition, isolated abnormal laboratory values that are considered clinically significant (e.g., cause study drug discontinuation or constitutes in and of itself a Serious Adverse Event) should be recorded on the Adverse Events CRF. SAEs occurring after signing the Informed Consent and prior to starting study treatment are recorded on the Medical History CRF if the patient continues on with the study or the Adverse Events CRF if the patient withdraws from the study prior to starting study treatment.

Adverse events will be assessed according to the NCI Common Toxicity Criteria for Adverse Events (CTCAE) version 3.0. If CTCAE grading does not exist for an adverse event, the severity of mild, moderate, severe, and life-threatening, corresponding to grades 1 - 4, will be used. CTCAE grade 5 (death) will not be used in this study; rather, this information will be collected on the End of Treatment and Study Evaluation Completion CRF pages. Adverse event monitoring should be continued for at least 28 days following the last dose of study treatment.

The occurrence of adverse events should be sought by non-directive questioning of the patient at each visit during the study. Adverse events also may be detected when they are volunteered by the patient during or between visits or through physical examination, laboratory test, or other assessments. As far as possible, each adverse event should be evaluated to determine:

1. The severity grade, CTCAE grade 1-4
2. Its relationship to study drug (suspected/not suspected)
3. Its duration (start and end dates or if continuing at final exam)
4. Action taken (no action taken; study drug dosage adjusted/temporarily interrupted; study drug permanently discontinued due to this adverse event; concomitant medication taken; non-drug therapy given; hospitalization/prolonged hospitalization)
5. Whether it is serious, where a serious adverse event (SAE) is defined as one which:
    - Is fatal or life-threatening
    - Results in persistent or significant disability/incapacity

- Constitutes a congenital anomaly/birth defect
- Requires inpatient hospitalization or prolongation of existing hospitalization, unless hospitalization is for:
  - Routine treatment or monitoring of the studied indication, not associated with any deterioration in condition
  - Elective or pre-planned treatment for a pre-existing condition that is unrelated to the indication under study and has not worsened since signing the informed consent
  - Treatment on an emergency outpatient basis for an event not fulfilling any of the definitions of an SAE given above and not resulting in hospital admission
  - Social reasons and respite care in the absence of any deterioration in the patient's general condition

6. Is medically significant, i.e., defined as an event that jeopardizes the patient or may require medical or surgical intervention to prevent one of the outcomes listed above

Unlike routine safety assessments, SAEs are monitored continuously and have special reporting requirements; see Section 8.1.

All adverse events should be treated appropriately. Such treatment may include changes in study drug treatment including possible interruption or discontinuation, starting or stopping concomitant treatments, changes in the frequency or nature of assessments, hospitalization, or any other medically required intervention. Once an adverse event is detected, it should be followed until its resolution, and an assessment should be made at each visit (or more frequently, if necessary) of any changes in its severity, its suspected relationship to the study drug(s), any of the interventions required to treat it, and its outcome.

Information about common side effects already known about the investigational drug can be found in the most recent version of the Investigator's Brochure (IB) and, in addition, will be communicated between IB updates in the form of Investigator Notifications. This information will be included in the patient informed consent and should be discussed with the patient during the study as needed.

7.6.2 Chest CT scan

Non-infectious pneumonitis is a known side effect of rapamycin analogues including RAD001. Clinically significant pneumonitis is typically accompanied by non-specific symptoms including dyspnea, nonproductive cough, fatigue, and fever. Diagnosis is generally suspected in individuals receiving mTOR inhibitors who develop these symptoms or in asymptomatic individuals in whom a routine chest CT scan reveals a new ground glass or alveolar infiltrate. The frequency of symptomatic pulmonary toxicity (all grades) was approximately 13% in a phase III study of RAD001 in patients with metastatic renal cell carcinoma ([CRAD001C2240]). Severe (CTC grade 3) pneumonitis occurred in 4% of patients, and an occasional fatality was reported. The lung toxicity was partly or completely reversible in the majority of cases with interventions including drug interruption, discontinuation and the use of corticosteroids.

Adherence to the recommendations in this protocol (including Table 6-3) should ensure detection of clinically relevant pneumonitis occurring in patients and its appropriate management. Pulmonary function tests, CT chest and referral to a pulmonologist should be considered at the investigator's discretion, if clinically indicated.

7.6.3 Physical examination

Physical examination must include a total body examination (general appearance, skin, neck, including thyroid, eyes, ears, nose, throat, lungs, heart, abdomen, back, lymph nodes, Tanner Staging, extremities and a basic nervous system neurological exam). Physical exams should also evaluate the attainment of growth and development milestones (age at thelarche (females), age at adrenarche (males) and age at menarche (females)). Significant findings from physical examination must be recorded either as Relevant Medical History/Current Medical Conditions (if present before treatment) or as Adverse Events (if newly occurring or worsening since starting treatment).

7.6.3.1 Tanner staging

(Marshal and Tanner 1969, 1970)

7.6.3.1.1 Males

Genitalia stages:

Stage 1: Pre-adolescent. Testes, scrotum, and penis are of about the same size and proportion as in early childhood.

Stage 2: The scrotum and testes have enlarged and there is a change in the texture of the scrotal skin. There is also some reddening of the scrotal skin.

Stage 3: Growth of the penis has occurred, at first mainly in length but with some increase in breadth. There has been further growth of testes and scrotum.

Stage 4: Penis further enlarged in length and breadth with development of glans. Testes and scrotum further enlarged. There is also further darkening of the scrotal skin.

Stage 5: Genitalia adult in size and shape. No further enlargement takes place after Stage 5 is reached.

Pubic Hair Stages:

Stage 1: Pre-adolescent. The velus over the pubesis no further developed than that over the abdominal wall, i.e. no pubic hair.

Stage 2: Sparse growth of long, slightly pigmented, downy hair, straight or only slightly curled, appearing chiefly at the base of the penis.

Stage 3: Considerably darker, coarser, and more curled. The hair spreads sparsely over the junction of the pubes.

Stage 4: Hair is now adult in type, but the area covered by it is still considerably smaller than in most adults. There is no spread to the medial surface of the thighs.

Stage 5: Adult in quantity and type, distributed as an inverse triangle of the classically feminine pattern. Spread to the medial surface of the thighs but not up the linea alba or elsewhere above the base of the inverse triangle.

7.6.3.1.2 Females

Breast stages:

Stage 1: Pre-adolescent; elevation of papilla only.

Stage 2: Breast bud stage; elevation of breast and papilla as a small mound, enlargement of areola diameter.

Stage 3: Further enlargement of breast and areola, with no separation of their contours.

Stage 4: Projection of areola and papilla to form a secondary mound above the level of the breast.

Stage 5: Mature stage; projection of papilla only, due to recession of the areola to the general contour of the breast.

Pubic Hair Stages:

Stage 1: Pre-adolescent; the vellus over the pubes is not further developed than that over the anterior abdominal wall, i.e. no pubic hair.

Stage 2. Sparse growth of long, slightly pigmented, downy hair, straight or only slightly curled, appearing chiefly along the labia.

Stage 3: Considerably darker, coarser, and more curled. The hair spreads sparsely over the junction of the pubes.

Stage 4: Hair is now adult in type, but the area covered by it is still considerably smaller than in most adults. There is no spread to the medial surface of the thighs.

Stage 5: Adult in quantity and type, distributed as an inverse triangle of the classically feminine pattern. Spread to the medial surface of the thighs, but not up the linea alba or elsewhere above the base of the inverse triangle.

7.6.4 Vital signs

Pulse, respiration rate, blood pressure and temperature, and height and weight will be measured as indicated in the Assessment schedule (Table 7-1 and Table 7-2) and will be recorded on source documents, and entered on CRF pages.

Blood pressure, pulse and respiration rate should be measured on patients after at least 3 minutes in the sitting position.

7.6.5 Body surface area

Body surface area (BSA) will be calculated by IWRS on Treatment Day 1, at 4, 8, 12, 18, and 24 weeks, and every 12 weeks thereafter as indicated in the Assessment schedules (Table 7-1 and Table 7-2). BSA (in m^2) will be calculated using the following formula where weight (W) is in *kilograms* and height (H) is in *centimeters* (Dubois and Dubois 1916):

$$BSA= (W^{0.425} x\ H^{0.725})\ x\ 0.007184$$

The most recent BSA value should be used to determine actual total daily dose of study treatment (in mg) each time study medication is dispensed.

7.6.6 Performance status scale

7.6.6.1 Lansky Play Performance Scale

Lansky Play Performance Status will be measured as indicated in the assessment schedules (Table 7-1 and Table 7-2), for patients aged 1 month to 12 years, at randomization. Patients assessed by Lansky Play Performance Scale at screening should continue to be assessed with this tool throughout the trial, even after they have reached the age of 13 years (note that the tool has been designed for children up to the age of 18). The Lansky Play Performance Status should be recorded on the CRF and in the source documents.

Lansky play performance scale

Grade 100:	Fully active, normal.
Grade 90:	Minor restrictions in physically strenuous activity.
Grade 80:	Active, but tires more quickly.
Grade 70:	Both greater restriction of, and less time spent in, active play.
Grade 60:	Up and around, but minimal active play, keeps busy with quieter activities.
Grade 50:	Gets dressed, but lies around much of the day; no active play; able to participate in all quiet play and activities.
Grade 40:	Mostly in bed; participates in quiet activities.
Grade 30:	In bed; needs assistance even for quiet play.
Grade 20:	Often sleeping; play entirely limited to very passive activities.
Grade 10:	No play; does not get out of bed. Moribund.

7.6.6.2 WHO performance status

WHO performance status will be measured as indicated in the assessment schedules (Table 7-1 and Table 7-2) for patients aged 13 and older. For these patients, WHO performance status should be recorded on the CRF and in the source documents.

Performance status WHO grade:

Grade 0:	Fully active, able to carry out all normal activity without restriction.
Grade 1:	Restricted in physically strenuous activity but ambulatory and able to carry out work of a light or sedentary nature, e.g., light house work, office work.
Grade 2:	Ambulatory and capable of all selfcare but unable to carry out any work activities. Up and about more than 50% of waking hours
Grade 3:	Capable of only limited selfcare, confined to bed or chair more than 50% of waking hours
Grade 4:	Completely disabled. Cannot carry on any selfcare. Totally confined to bed or chair
Grade 5:	Dead.

7.6.7 Laboratory evaluations

All standard clinical laboratory analyses described below are to be performed by the central laboratory, according to the Visit Schedules outlined in Table 7-1 and Table 7-2. The name of the central laboratory can be found in the investigator binder supplied to the site. Details about all central laboratory procedures including collection, shipment of samples, reporting of results and alerting of extreme values are given in the manual provided by the central laboratory. Notable values will be provided in the laboratory manual.

For clinically relevant laboratory values from local labs, please record actual laboratory value on the CRF and provide Lab Normal Range sheet, as appropriate. Unscheduled abnormal laboratory evaluations which are clinically relevant (e.g., require dose modification and/or interruption of study drug, indicate changes in previously abnormal values) must be recorded on the Adverse Events CRF.

Screening safety laboratories (hematology, biochemistry, lipid panel, urinalysis and coagulation) will need to be repeated at Treatment Day 1 if they were collected more than 14 days prior. For the week 6 visit (visit 5 in the blinded portion and visit 105 in the open label/extension portion), patients for whom travel is difficult, who have tolerated study medication and have no adverse events may, at the discretion of the investigator, attend a local laboratory for blood draws (if such facility is available) and have a telephone consultation with the investigator rather than a clinic visit (detailed instructions are provided in the Quest Diagnostics [Laboratory Manual]).

7.6.7.1 Hepatitis screen

Patients who have a positive test for hepatitis B or C at screening must be excluded from the study unless the positive Hepatitis B antibody test resulted from prior vaccination. Hepatitis testing will be done only at screening (see Table 7-1) for patients that meet the criteria outlined in Section 4.1. Guidelines for those patients that are active on study prior to the approval of amendment 2 are located in Table 4-1 and Table 4-2.

If an already randomized patient (at the time of Amendment 2 approval) tests positive for Hepatitis C, please refer to Table 7-1 and Table 7-2 for HCV RNA-PCR monitoring schedule for those patients with positive HCV RNA-PCR baseline tests who do not meet the HCV flare criteria outlined in Table 4-3. If the patient tests positive for hepatitis C, and the criteria for HCV flare according to Table 4-3 are observed, trial therapy should be discontinued and further treatment is up to the investigators discretion.

7.6.7.2 Hematology

Hematology tests are to be performed at each scheduled visit as indicated in Table 7-1 and Table 7-2. These must include: hemoglobin, hematocrit, platelets, red blood cell count (RBC), total white blood cell count (WBC) and absolute & differential (including neutrophils, lymphocytes, monocytes, eosinophils, basophils). Absolute neutrophil count (ANC) will be calculated by the laboratory.

7.6.7.3 Coagulation

Prothrombin time (PT) will be determined at screening and every 12 weeks while on study (at 12 and 24 weeks and every 12 weeks thereafter); it will be reported as international normalized ratio (INR). In addition, fibrinogen and partial thromboplastin (PTT) will be determined at screening and every 12 weeks while on study.

7.6.7.4 Biochemistry and lipid profile

The following tests will be performed at each scheduled visit as indicated in Table 7-1 and Table 7-2 and will include sodium, potassium, chloride, bicarbonate, creatinine, albumin, total protein, SGOT (AST), SGPT (ALT), total bilirubin, alkaline phosphatase, uric acid, BUN, calcium, magnesium, phosphate, total LDH, and fasting glucose.

In order to assess renal function during the trial, an age appropriate measure; either the Cockcroft-Gault formula for patients 18 years and older or the Schwartz formula for patients up to age 18 (Schwartz, et al, 2009) will be used. The Cockcroft-Gault formula is shown below, where creatinine clearance is “*x*” (in mL/min), age is measured in years, weight in kg, creatinine in µmol/L, and the constant is 1.23 for men and 1.04 for women (Cockcroft-Gault 1976):

$$x = \frac{(140 - age) \times weight \times constant}{creatinine}$$

The Schwartz formula is shown below, where Glomerular Filtration Rate (GFR) is “*x*” (in mL/min/1.73 m^2), height is measured in cm and serum creatinine in mg/dL (Schwartz et al, 2009).

$$x = (0.41 \text{ x } Height) / Serum\ creatinine$$

A lipid profile (cholesterol, triglycerides, LDL, HDL) will be determined at screening and repeated every 12 weeks while receiving study drug (at 12 and 24 weeks and every 12 weeks thereafter). The patient must be in a fasting state at the time of blood sampling for this evaluation.

7.6.7.5 Urinalysis

During screening, a standard urinalysis assessment (pH, protein, glucose, blood, ketones, and leukocytes) should be performed and submitted to the central laboratory. Urine dipstick will be performed routinely thereafter at each study visit (excluding visits 2, 3 and 5). This must be supplemented with central laboratory quantification of any potentially relevant abnormalities.

7.6.7.6 Pregnancy test

All females of child-bearing potential must have a negative serum pregnancy test at screening/baseline as well as a negative urine pregnancy test (performed locally) prior to treatment on Treatment Day 1. Urine pregnancy tests will then be repeated every 4 weeks until discontinuation of study drug and serum pregnancy tests will be repeated at the end of the study. Patient should be instructed to inform site of a positive urine pregnancy result. Repeat serum pregnancy testing will be performed for confirmation of a positive urine pregnancy test. It is recommended that postmenopausal women be amenorrheic for at least 12

months or have a serum follicle-stimulating hormone (FSH) of >40 mIU/ml to be considered "of non-childbearing potential" or 6 weeks post surgical bilateral oophorectomy with or without hysterectomy.

Highly effective contraception, must be used on-study and for up to 8 weeks after ending treatment (definition of highly effective contraception is detailed in Section 7.3.2.1).

To ensure patient safety, each pregnancy in a patient on study drug must be reported to Novartis within 24 hours of learning of its occurrence. The pregnancy should be followed up to determine outcome, including spontaneous or voluntary termination, details of the birth, and the presence or absence of any birth defects, congenital abnormalities, or maternal and/or newborn complications.

Pregnancy should be recorded on a Clinical Trial Pregnancy Form and reported by the investigator to the local Novartis Integrated Medical Safety ("IMS") Department. Pregnancy follow-up should be recorded on the same form and should include an assessment of the possible relationship to the Novartis study drug of any pregnancy outcome. Any SAE experienced during pregnancy must be reported on the SAE Report Form.

7.6.7.7 Reproductive history and endocrine testing

In order to provide additional follow-up on cases of amenorrhea, increased hormonal evaluation will be performed and supplemental medical history will be collected.

Additional menstrual history (previous cases of amenorrhea or menstrual disorders, biological mother's age at menopause) and pregnancy history (pregnancies, full-term gestations, abortions, live births, miscarriages) will be collected one time after implementation of protocol amendment 6. Following implementation of amendment 6, menstrual status will be collected monthly via patient diary. Every 12 weeks, data from patient diary will be collected during patient's visit at clinical site for CRF entry. It is recommended that study coordinators contact patients monthly for the first 3 months to remind patients to document menstrual status in patient diary.

A blood sample for analysis of total testosterone, FSH and LH and estradiol (females) will be collected annually until the patient's 10th birthday and then every 12 weeks thereafter for endocrine assessment. In the event that amenorrhea is reported in between scheduled assessments, hormone evaluations should be completed at that time.

For patients who are active prior to the approval of Amendment 4 the above screening should be completed at the next scheduled visit, if it had not been collected previously (considered baseline) at the intervals appropriate for their age as indicated above.

7.6.8 Pulmonary function tests

Individuals participating in this trial will be questioned at each visit as to the presence of new or changed pulmonary symptoms consistent with lung toxicity. If an investigator suspects a patient may be developing pneumonitis investigations such as pulmonary function tests, CT chest and referral to a pulmonologist should be considered.

A bronchoscopy with biopsy and/or a bronchoalveolar lavage (BAL) will be performed only when medically necessary for ensuring patient care (details are provided in Table 6-3) Electrocardiogram (ECG)

A standard 12-lead ECG is to be performed during screening. Tracings must be dated and signed by the investigator (or his/her designee) and filed with the subject's source documentation. Significant findings must be recorded as Relevant Medical History / Current Medical Conditions (if present before treatment). ECG may be repeated at the discretion of the investigator at any time during the study and as clinically indicated; any clinically relevant findings should be recorded on the Adverse Events CRF.

7.7 Pharmacokinetics

Biofluid concentrations will be expressed in mass per volume units. All concentrations below the limit of quantification or missing data will be labeled as such in the concentration data listings. Concentrations below the limit of quantification will be treated as zero in summary statistics.

Patients will be advised to fast overnight on the days of blood sampling for RAD001 prior to the sampling period (note: patients are allowed to consume water during this time). A meal may be taken after the predose sample has been collected, but prior to ingestion of the daily RAD001 dose; patients should be advised to avoid fatty meals.

7.7.1 Pharmacokinetic (PK) blood sample collection and handling

Pre-dose trough blood samples for determination of RAD001 concentration (C_{min}) will be collected immediately prior to dosing starting on Visit 3 (Week 2) and then at every visit thereafter until discontinuation of study drug. **A sample of blood collected 22-26 hours for daily dosing (or 46-50 hours for every other day dosing) after the patient's last dose of study drug following 5 days of consistent (daily dose and timing of dose) dosing is a trough PK blood sample. Any sample collected outside of these parameters is not considered a true trough and therefore should not be used as a basis for adjusting the patient's dose.** Pre-dose trough blood samples will be collected in both treatment arms during regularly scheduled visits by either direct venipuncture or an inserted indwelling cannula. Please refer to Table 7-4 and Table 7-5 for amount of blood to be drawn for each trough PK assessment and the PK sample number that should be assigned to each sample.

Each patient will start with a dose of 4.5 mg/m^2/day. Trough (pre-dose) blood levels of RAD001 will be assessed after 2 weeks. A local lab (at the treating center) will collect the blood samples and send them to a central laboratory to perform the PK analysis. In addition,

trough blood levels of RAD001 will be assessed 1-2 weeks after increasing the dose to a level which has not previously been taken.

On Visit 3 (Week 2) and at two weeks after any dose adjustment, a PK blood sample (2 mL) will be taken at 2 hours (± 30 mins, C_{2h}) after trial therapy dose administration. Table 7-4 and Table 7-5 shows the PK sample number that should be assigned to each of these samples.

Everolimus trough levels are taken routinely at every clinic visit.

Samples for trough (24 h after the last dose) and C_{max} (2.0 hours ± 30 mins after dosing) will be taken two weeks after the following events:

1. everolimus dose increase to a higher level than previously taken
2. reduction in the dose of a CYP3A4 or PgP inducer (e.g. reduction in anticonvulsant dose)
3. starting, or increasing the dose of, a CYP3A4 or PgP inhibitor

Trough and C_{max} are not required for dose re-escalation to a previously-used dose; or for dose decreases. Patients in these situations will have sampling done at the next routine visit.

PK sampling which is not undertaken at a routine clinic visit (i.e. two week post dose increase) may, if such facility is available, be undertaken at a local laboratory (Detailed instructions are provided in the Quest Laboratory Manual).

- For the 6 week visit (visit 5 in the blinded portion and visit 105 in the open label/extension portion), patients for whom travel is difficult, who have tolerated study medication and have no adverse events may, at the discretion of the investigator, attend a local laboratory for blood draws (if such facility is available) and have a telephone consultation with the investigator rather than a clinic visit (detailed instructions are provided in the Quest Diagnostics Laboratory Manual).
- Patients who are active in the extension phase at the time of Amendment 5 approval and consent to participate will be asked to have additional PK blood samples collected at any ONE of their regularly scheduled visits. Patients will be divided among the following age groups:
  - < 5 years old
  - ≥5 and <10 years old
  - ≥10 and <14 years old
  - ≥14 and <18 years old and
  - ≥18 years old

Additional PK samples (2 mL/sample for patients 10 years and older and 1 mL/sample for younger patients) will be collected at steady-state condition at pre-dose and at 0.5, 1, 2, 5 and 24 hours after the patient's dose on the day of a study visit. The originally planned predose and 2-hour post-dose samples (if required) will not be collected. The time window is 22-26h after the last dose for the pre-dose sample, ± 10 minutes for the 0.5-, 1-, and 2-hour samples, ± 1 hour for the 5-hour sample, and ± 2 hours for the 24-hour sample. Steady-state condition is defined as continuous administration of the same dose of study drug to the patient in the previous 5 days and on the day of the collection of the PK samples and the patient has not vomited within 4 hours of the last dosing.. The samples should not be collected if a patient is not at steady-state,

High fat food intake has been shown to effect concentration levels of everolimus. Patients participating in the full PK profile assessment should follow the following meal intake guidelines prior to and after receiving their everolimus dose. Patients are required to be in fasting state when they arrive to the clinic for study visits. Following the blood collection of safety labs and the predose PK sample, a light, low fat meal should be provided and then the patient should receive their everolimus dose immediately after consuming the meal. Recommendations for a low fat meal are as follows (portions will be patient/age dependent):

- Cereal with low fat milk, a piece of toast with jam and/or butter, a glass of orange juice, and a banana

No high fat meal should be consumed within 4 hours after dose administration on the day of full PK profile sampling. Following this time frame, the patient can eat as normal.

Table 7-4 PK Sample log table for visits 1-99 (Refer to Table 7-1)

Study Day	Study Week	Time	PK Collection Number	PK Sample Number	Sample Volume (mL)
Day 14*	Week 2	Pre-dose	101	101	2 mL
Day 14*	Week 2	2 h (± 30 min)	102	102	2 mL
Day 28*	Week 4	Pre-dose	103	103	2 mL
Day 28*	Week 4	2 h (± 30 min)	104	104	2 mL
Day 42*	Week 6	Pre-dose	105	105	2 mL
Day 42*	Week 6	2 h (± 30 min)	106	106	2 mL
Day 56*	Week 8	Pre-dose	107	107	2 mL
Day 56*	Week 8	2 h (± 30 min)	108	108	2 mL
Day 70	Week 10	Pre-dose	109	109	2 mL
Day 70	Week 10	2 h (± 30 min)	110	110	2 mL
Day 84*	Week 12	Pre-dose	111	111	2 mL
Day 84*	Week 12	2 h (± 30 min)	112	112	2 mL
Day 98	Week 14	Pre-dose	113	113	2 mL
Day 98	Week 14	2 h (± 30 min)	114	114	2 mL
Day 112	Week 16	Pre-dose	115	115	2 mL
Day 112	Week 16	2 h (± 30 min)	116	116	2 mL
Day 126*	Week 18	Pre-dose	117	117	2 mL
Day 126*	Week 18	2 h (± 30 min)	118	118	2 mL
Day 140	Week 20	Pre-dose	119	119	2 mL
Day 140	Week 20	2 h (± 30 min)	120	120	2 mL
Day 154	Week 22	Pre-dose	121	121	2 mL
Day 154	Week 22	2 h (± 30 min)	122	122	2 mL
Day 168*	Week 24	Pre-dose	123	123	2 mL
Day 168*	Week 24	2 h (± 30 min)	124	124	2 mL
Day 182	Week 26	Pre-dose	125	125	2 mL
Day 182	Week 26	2 h (± 30 min)	126	126	2 mL
Day 196	Week 28	Pre-dose	127	127	2 mL
Day 196	Week 28	2 h (± 30 min)	128	128	2 mL
Day 210	Week 30	Pre-dose	129	129	2 mL

Study Day	Study Week	Time	PK Collection Number	PK Sample Number	Sample Volume (mL)
Day 210	Week 30	2 h (± 30 min)	130	130	2 mL
Day 224	Week 32	Pre-dose	131	131	2 mL
Day 224	Week 32	2 h (± 30 min)	132	132	2 mL
Day 238	Week 34	Pre-dose	133	133	2 mL
Day 238	Week 34	2 h (± 30 min)	134	134	2 mL
Day 252*	Week 36	Pre-dose	135	135	2 mL
Day 252*	Week 36	2 h (± 30 min)	136	136	2 mL
Day 266	Week 38	Pre-dose	137	137	2 mL
Day 266	Week 38	2 h (± 30 min)	138	138	2 mL
Day 280	Week 40	Pre-dose	139	139	2 mL
Day 280	Week 40	2 h (± 30 min)	140	140	2 mL
Day 294	Week 42	Pre-dose	141	141	2 mL
Day 294	Week 42	2 h (± 30 min)	142	142	2 mL
Day 308	Week 44	Pre-dose	143	143	2 mL
Day 308	Week 44	2 h (± 30 min)	144	144	2 mL
Day 322	Week 46	Pre-dose	145	145	2 mL
Day 322	Week 46	2 h (± 30 min)	146	146	2 mL
Day 336*	Week 48	Pre-dose	147	147	2 mL
Day 336*	Week 48	2 h (± 30 min)	148	148	2 mL
	Every 2 weeks after	Pre-dose	149, 151, 153, 155….	149, 151, 153, 155….	2 mL
	Every 12 weeks after*	Pre-dose	159, 171, 183, 195, 207, 219, 231….	159, 171, 183, 195, 207, 219, 231….	2 mL
	Every 2 weeks after	2 h (± 30 min)	150, 152, 154, 156….	150, 152, 154, 156….	2 mL
	Every 12 weeks after*	2 h (± 30 min)	160, 172, 184, 196, 208, 220, 232….	160, 172, 184, 196, 208, 220, 232….	2 mL

*Denotes required PK collection visits as reflected in Table 7-1 and Table 7-2. All other visits listed in Table 7-5 are to be referenced for entry into the Case Report Form in cases where the patient needs to return for a 2-week post medication change visit (unscheduled) due to one of the scenarios listed above (i.e. study drug dose increase). If a patient does not need to return in between regularly scheduled visits, the information for the visits in between should be skipped. The PK information above is specifically assigned to a potential visit and is not to be used for sequential PK collections.

Note: For PK blood collections after week 48, please refer to the detailed PK Sample Log that is provided in the CRF and included in the Investigator Portal.

Table 7-5 PK sample log table for visits 101-199 (Refer to Table 7-2)

Study day	Study Week	Time	PK Collection Number	PK Sample Number	(mL)
Day 14*	Week 2	Pre-dose	501	501	2 mL
Day 14*	Week 2	2 h (± 30 min)	502	502	2 mL
Day 28*	Week 4	Pre-dose	503	503	2 mL
Day 28*	Week 4	2 h (± 30 min)	504	504	2 mL
Day 42*	Week 6	Pre-dose	505	505	2 mL
Day 42*	Week 6	2 h (± 30 min)	506	506	2 mL
Day 56*	Week 8	Pre-dose	507	507	2 mL
Day 56*	Week 8	2 h (± 30 min)	508	508	2 mL
Day 70	Week 10	Pre-dose	509	509	2 mL
Day 70	Week 10	2 h (± 30 min)	510	510	2 mL
Day 84*	Week 12	Pre-dose	511	511	2 mL
Day 84*	Week 12	2 h (± 30 min)	512	512	2 mL
Day 98	Week 14	Pre-dose	513	513	2 mL
Day 98	Week 14	2 h (± 30 min)	514	514	2 mL
Day 112	Week 16	Pre-dose	515	515	2 mL
Day 112	Week 16	2 h (± 30 min)	516	516	2 mL
Day 126*	Week 18	Pre-dose	517	517	2 mL
Day 126*	Week 18	2 h (± 30 min)	518	518	2 mL
Day 140	Week 20	Pre-dose	519	519	2 mL
Day 140	Week 20	2 h (± 30 min)	520	520	2 mL
Day 154	Week 22	Pre-dose	521	521	2 mL
Day 154	Week 22	2 h (± 30 min)	522	522	2 mL
Day 168*	Week 24	Pre-dose	523	523	2 mL
Day 168*	Week 24	2 h (± 30 min)	524	524	2 mL
Day 182	Week 26	Pre-dose	525	525	2 mL
Day 182	Week 26	2 h (± 30 min)	526	526	2 mL
Day 196	Week 28	Pre-dose	527	527	2 mL
Day 196	Week 28	2 h (± 30 min)	528	528	2 mL
Day 210	Week 30	Pre-dose	529	529	2 mL
Day 210	Week 30	2 h (± 30 min)	530	530	2 mL
Day 224	Week 32	Pre-dose	531	531	2 mL
Day 224	Week 32	2 h (± 30 min)	532	532	2 mL
Day 238	Week 34	Pre-dose	533	533	2 mL

Study day	Study Week	Time	PK Collection Number	PK Sample Number	(mL)
Day 238	Week 34	2 h (± 30 min)	534	534	2 mL
Day 252*	Week 36	Pre-dose	535	535	2 mL
Day 252*	Week 36	2 h (± 30 min)	536	536	2 mL
Day 266	Week 38	Pre-dose	537	537	2 mL
Day 266	Week 38	2 h (± 30 min)	538	538	2 mL
Day 280	Week 40	Pre-dose	539	539	2 mL
Day 280	Week 40	2 h (± 30 min)	540	540	2 mL
Day 294	Week 42	Pre-dose	541	541	2 mL
Day 294	Week 42	2 h (± 30 min)	542	542	2 mL
Day 308	Week 44	Pre-dose	543	543	2 mL
Day 308	Week 44	2 h (± 30 min)	544	544	2 mL
Day 322	Week 46	Pre-dose	545	545	2 mL
Day 322	Week 46	2 h (± 30 min)	546	546	2 mL
Day 336*	Week 48	Pre-dose	547	547	2 mL
Day 336*	Week 48	2 h (± 30 min)	548	548	2 mL
	Every 2 weeks thereafter	Pre-dose	549, 551, 553, 555….	549, 551, 553, 555….	2 mL
	Every 12 weeks thereafter*	Pre-dose	559, 571, 583, 595, 607, 619, 631….	559, 571, 583, 595, 607, 619, 631….	2 mL
	Every 2 weeks thereafter	2 h (± 30 min)	550, 552, 554, 556….	550, 552, 554, 556….	2 mL
	Every 12 weeks thereafter*	2 h (± 30 min)	560, 572, 584, 596, 608, 620, 632….	560, 572, 584, 596, 608, 620, 632….	2 mL

*Denotes required PK collection visits as reflected in Table 7-1 and Table 7-2. All other visits listed in Table 7-5 are to be referenced for entry into the Case Report Form in cases where the patient needs to return for a 2-week post medication change visit (unscheduled) due to one of the scenarios listed above (i.e. study drug dose increase). If a patient does not need to return in between regularly scheduled visits, the information for the visits in between should be skipped. The PK information above is specifically assigned to a potential visit and is not to be used for sequential PK collections.

Note: For PK blood collections after week 48, please refer to the detailed PK Sample Log that is provided in the CRF and included in the Investigator Portal.

Table 7-6 PK sample log for full PK profile (Amendment 5)

Time	PK Collection Number	PK Sample Number	Volume (mL)*
Predose	401	401	2 mL
0.5 h	402	402	2 mL
1 h	402	403	2 mL

Time	PK Collection Number	PK Sample Number	Volume (mL)*
2 h	402	404	2 mL
5 h	402	405	2 mL
24 h	402	406	2 mL

* 1 mL blood sample for patients < 10 years of age.

RAD001 blood sample collection

A 2-mL venous blood sample will be drawn for RAD001 blood concentration determination from a forearm vein into the tubes containing EDTA. The tube will be inverted several times to mix contents (e.g., anti-coagulant) of the tube immediately after collection of the blood sample. Prolonged contact must be avoided with rubber stopper. The whole blood sample will be transferred to a labeled polypropylene screw cap tube and frozen at - 20°C or below within 60 minutes of venipuncture. An example of the label that should be attached to the tube is shown below. The respective PK sample number shown in Table 7-4, Table 7-5 or Table 7-6 should be entered on each label next to "sample number."

PK labels will be designed as follows:

Study number	CRAD001M2301
Subject initials and number	_ _ _ / _ _ _ _ - _ _ _ _ _
Analyte	Everolimus
Sample number	_ _ _

Sample collection handling

The actual collection time of all samples must be documented on the PK Blood Collection CRF pages. **The date and actual time of the last dose of study drug and date and time of the dose of study drug taken on the day of the sampling must be recorded on the PK Blood Collection CRF. In addition, the date and times of blood samples must be entered on the PK Blood Collection CRF**. Any sampling problems (i.e., patient took study drug before a trough [pre-dose]) must be noted in the comments section of the CRF.

In order to assure compliance with sampling procedures, **on days of drug level and PK assessment, drug administration should be supervised by study center personnel.**

If the patient's dose has been interrupted for more than 48 hours prior to when a PK blood collection is required, it is unnecessary to collect the PK sample. If the patient vomits within the first 4 hours following study-drug administration on the day of PK blood sampling, the time (using the 24-hour clock) of vomiting should be recorded on the Dosage Administration Record visit level CRFs. No additional study drug should be taken that day to replace the material that has been vomited.

7.7.2 RAD001 pharmacokinetic sample shipment

All samples must be carefully packed in suitable packing material containing sufficient dry ice to keep them frozen during shipment.

A list of all samples, including the date, subject number, and time of sampling should be sent with the shipment. Any missing samples should be notified on the list.

Samples will be sent to Clinical Reference Laboratory 11842 W 85th St., Lenexa, KS 66215. Shipments should all be sent on Monday, Tuesday, Wednesday, Thursday or Friday using a carrier guaranteeing overnight delivery (e.g., World Courier).

For details regarding PK sample shipment, please refer to the laboratory manual.

7.7.3 Analytical method

RAD001 blood concentrations in whole blood will be determined by a LC-MS method. The method has a lower limit of quantification (LLOQ) of 0.3 ng/mL.

7.8 Biomarkers

Biomarker studies are proposed using patients' plasma samples. These studies will focus on measuring the effect of RAD001 on soluble markers of angiogenesis. mTOR inhibitors have been shown to have an inhibitory effect on tumor growth and angiogenesis both in vitro and in vivo. VEGF and its family members are essential mediators of tumor angiogenesis.

We plan to examine the effects of RAD001 on tumor vascularization through the measurement of these angiogenic growth factors and their corresponding soluble receptors. Data from these studies will be used to formulate hypotheses for future studies of RAD001 as an anti-angiogenic agent.

All patients will donate 3 mL of blood for plasma at the following time points (also refer to Table 7-1) (unless local or national regulations do not permit):

- screening/baseline
- 4 weeks
- 12 weeks
- 24 weeks
- 36 weeks
- 48 weeks
- End of Treatment

The blood samples for biomarker assessments should be collected immediately prior to drug administration. On-treatment samples will be compared to baseline samples for RAD001 effects on plasma angiogenic molecules, e.g., basic FGF, VEGF, PLGF, soluble VEGF receptor1 and soluble VEGF receptor2. Analysis will be performed using standard ELISA technology as well as multiplexed MSD platform formatted as 4plex and 2plex combinations, following validation of assay according to manufacturer's specifications.

8 Safety monitoring

8.1 Serious adverse event reporting

To ensure patient safety, every SAE, **regardless of suspected causality**, occurring after the patient has provided informed consent and until 4 weeks after the patient has stopped study treatment/participation must be reported to Novartis within 24 hours of learning of its occurrence. Any SAEs experienced after this 4-week period **(for up to 84 days (12 weeks))** should only be reported to Novartis if the investigator suspects a causal relationship to the study drug. Recurrent episodes, complications, or progression of the initial SAE must be reported as follow-up to the original episode within 24 hours of the investigator receiving the follow-up information. An SAE occurring at a different time interval or otherwise considered completely unrelated to a previously reported one should be reported separately as a new event.

Information about all SAEs is collected and recorded on the Serious Adverse Event Report Form. The investigator must assess and record the relationship of each SAE to each specific study drug (if there is more than one study drug), complete the SAE Report Form in English, and send the completed, signed form by fax within 24 hours to the local Novartis affiliate.

The telephone and fax numbers of the contact persons in the local Novartis Integrated Medical Safety Department, specific to the site, are listed in the investigator folder provided to each site. The original copy of the SAE Report Form and the fax confirmation sheet must be kept with the CRF documentation at the study site.

Follow-up information is sent to the same person to whom the original SAE Report Form was sent, using a new SAE Report Form stating that this is a follow-up to the previously reported SAE and giving the date of the original report. Each recurrence, complication, or progression of the original event should be reported as a follow-up to that event regardless of when it occurs. The follow-up information should describe whether the event has resolved or continues, if and how it was treated, whether the blind was broken or not, and whether the patient continued or withdrew from study participation.

If the SAE is not previously documented in the current version of the Investigator's Brochure, or Package Insert (new occurrence) and is thought to be related to the Novartis study drug, an Integrated Medical Safety associate may urgently require further information from the investigator for Health Authority reporting. Novartis may need to issue an Investigator Notification (IN), to inform all investigators involved in any study with the same drug that this SAE has been reported.

8.2 Pregnancies

To ensure patient safety, each pregnancy in a patient on study drug must be reported to Novartis within 24 hours of learning of its occurrence. The pregnancy should be followed up to determine outcome, including spontaneous or voluntary termination, details of the birth, and the presence or absence of any birth defects, congenital abnormalities, or maternal and/or newborn complications.

Pregnancy should be recorded on a Clinical Trial Pregnancy Form and reported by the investigator to the oncology Novartis Integrated Medical Safety (IMS) department at the local Novartis office. Pregnancy follow-up should be recorded on the same form and should include an assessment of the possible relationship to the Novartis study drug of any pregnancy outcome. Any SAE experienced during pregnancy must be reported on the SAE Report Form.

Preclinical data regarding reproductive toxicity is described in the most recent Investigator Brochure. The potential reproductive risk for humans is unknown.

Women of childbearing potential should be advised to use highly effective contraception methods while they are receiving everolimus and up to 8 weeks after treatment has been stopped.

If a pregnancy occurs while on study treatment, the newborn will be followed for at least 12 months.

8.3 Data Monitoring Committee

An Data Monitoring Committee (DMC) will be established prior to the randomization of the first patient; the same DMC will be used in any other Novartis-sponsored trials evaluating RAD001 in TSC-related diseases. The DMC is an external independent group including at least two physicians with expertise in TSC and one statistician. The DMC will perform the first safety review approximately 6 months after randomization of the first patient in any of the Novartis-sponsored RAD001 trials in TSC, and every 6 months thereafter, unless otherwise requested by the Chairman of the DMC. The DMC will also receive reports on a regular basis on all SAEs reported for this trial. No interim analysis is planned. Recruitment will not be interrupted unless otherwise requested by the Chairman of the DMC.

The responsibilities of the DMC include:

- minimize the exposure of patients to an unsafe therapy or dose
- make recommendations for changes in study processes where appropriate
- advise on the need for dose adjustments because of safety issues
- endorse continuation of the study

Details on the membership, responsibilities and working procedures of the DMC are described in the Data Monitoring Committee Charter.

8.4 Steering Committee

The general role of the steering committee is to provide guidance on study conduct, to help ensure delivery of study data and to develop study-related publications in accordance with the Novartis publication and authorship policy. The steering committee will support the Novartis clinical team on a continuous basis when questions arise in the trial. The steering committee will monitor and supervise the progress of the trial towards its objectives. The committee will be appointed by Novartis and will include two principal investigators from this trial, Novartis staff and possibly other clinical experts. The committee will be chaired by one of the two

9 Data review and data management

9.1 Site monitoring

Before study initiation, at a site initiation visit or at an investigator’s meeting, a Novartis representative will review the protocol and CRFs with the investigators and their staff. During the study, the field monitor will visit the site regularly to check the completeness of patient records, the accuracy of entries on the CRFs, the adherence to the protocol and to Good Clinical Practice, the progress of enrollment, and to ensure that study drug is being stored, dispensed, and accounted for according to specifications. Key study personnel must be available to assist the field monitor during these visits.

The investigator must maintain source documents for each patient in the study, consisting of case and visit notes (hospital or clinic medical records) containing demographic and medical information, laboratory data, electrocardiograms, and the results of any other tests or assessments. All information on CRFs must be traceable to these source documents in the patient's file. The investigator must also keep the original informed consent form signed by patient (a copy is given to the patient).

The investigator must give the monitor access to all relevant source documents to confirm their consistency with the CRF entries. Novartis monitoring standards require full verification of the presence of informed consent, adherence to the inclusion/exclusion criteria, documentation of SAEs, and the recording of data that will be used for all primary and safety variables. Additional checks of the consistency of the source data with the CRFs are performed according to the study-specific monitoring plan. No information in source documents about the identity of the patients will be disclosed.

9.2 Data collection

Designated investigator staff must enter the information required by the protocol onto the Novartis CRFs that are printed on 3-part, non-carbon-required paper. The PK summary pages of the CRF will also include an additional non-carbon required page, which will be sent to the bioanalytics pharmacokinetics group by the field monitor. All other summary pages will also include an additional non-carbon page that will be sent to Novartis by the field monitor. Field monitors will review the CRFs for completeness and accuracy and instruct site personnel to make any required corrections or additions. The CRFs are forwarded to the Contract Research Organization (CRO) by field monitors or by the investigational site, one copy being retained at the investigational site. Once the CRFs are received by the CRO, their receipt is recorded, the original copy is placed in Central Files, and the non-carbon-required copy is forwarded to the responsible data management staff for processing.

Once the data from the CRFs are entered into the database, automatic validation programs check for data discrepancies in the CRFs and generate appropriate error messages (queries) which will be sent to the site for resolution by the designated CRO. The responses to these queries will be used to update the database.

The investigator must certify that the data are complete and accurate by signing a memo that will be sent to him/her by the CRO after the last transfer of the data prior to analysis. After database lock, the investigator will receive a memo detailing the obvious corrections that were made to the data.

Blood samples for laboratory data and biomarkers will be collected (unless local or national regulations do not permit) by sites and sent to a central laboratory for processing. The laboratory results will be sent electronically to the designated CRO.

Results from at home urine pregnancy will be recorded on patient diaries for source documentation only. Following implementation of Amendment 6, menstrual status will be collected monthly via patient diary for source documentation. Every 12 weeks during patient's scheduled visit, data from patient diary will be collected at clinical site for CRF. It is recommended that study coordinators contact patients monthly for the first 3 months to remind patients to document menstrual status in patient diary.

9.3 Database management and quality control

The designated CRO staff will review the CRFs entered by investigational staff for completeness and accuracy and instruct the site personnel to make any required corrections or additions. Obvious errors will be corrected by the designated CRO personnel. Queries will be sent (faxed) to the investigational site using a paper Data Query Form. Designated investigator site staff should respond to the query and make any necessary changes to the data. Site personnel will complete and sign the faxed copy and fax it back to the CRO staff who will make the correction to the database. The signed copy of the Data Query Form is kept at the investigator site.

Concomitant medications entered into the database will be coded using the WHO Drug Reference List, which employs the Anatomical Therapeutic Chemical (ATC) classification system. Medical history/current medical conditions and adverse events will be coded using the Medical Dictionary for Regulatory Activities (MedDRA) terminology.

Laboratory samples will be processed centrally and the results will be sent electronically to the designated CRO.

Randomization codes and data about all study drug dispensed to the patient will be tracked using IWRS. The system will be supplied by a vendor, who will also manage the database. The database will be sent electronically to the designated CRO.

PK blood samples collected by sites will be shipped to a central laboratory for processing. The PK blood collection times will be entered by sites onto the CRFs. The PK blood sample results will be merged with the CRF PK blood collection times and analyzed by Novartis and in accordance with internal Novartis procedures.

Blood samples for biomarkers will be collected (unless local or national regulations do not permit) and sent to a central laboratory for processing. The results will be sent electronically to the designated CRO.

At the conclusion of the study, the occurrence of any emergency code breaks will be determined after return of all code break reports and unused drug supplies to Novartis. The occurrence of any protocol deviations will be determined. After these actions have been completed and the database has been declared to be complete and accurate, it will be locked and the treatment codes will be unblinded and made available for data analysis. Any changes to the database after that time can only be made by joint written agreement between the Global Head of Biostatistics and Statistical Reporting and the Global Therapeutic Area Head.

10 Statistical methods and data analysis

It is planned that the data from all centers that participate in this protocol will be used so that an adequate number of patients will be available for analysis.

The data will be analyzed by Novartis. Any data analysis carried out independently by the investigator should be submitted to Novartis before publication or presentation.

10.1 Populations for analysis

The **Full Analysis Set (FAS)** consists of all randomized patients. Following the intent-to-treat principle, patients are analyzed according to the treatment and stratum that they were assigned to at randomization. The FAS will be the primary population in the assessment of efficacy.

The **Per Protocol Set (PPS)** will consist of all patients from the FAS without any major protocol deviation, who are evaluable for efficacy and who have completed a minimum exposure requirement. However, if a patient had SEGA progression, discontinued for adverse event or died before the minimum exposure requirement could be met, or before he/she was evaluated for efficacy, that patient will still be included in the PPS. Patients will be evaluable for efficacy if they have a known SEGA response status. The minimum exposure requirement is defined as having received treatment on at least 50% of the days in the first 12 weeks since the first day of treatment. The PPS will be used for secondary analyses of efficacy.

The **Safety Population** will consist of all patients who received at least one dose of study treatment and had at least one post-baseline safety assessment (where the statement that a patient had no adverse event (on the Adverse Events CRF) constitutes a safety assessment). Patients will be analyzed according to treatment received.

10.2 Patient demographics/other baseline characteristics

Demographic and other baseline characteristics will be listed and summarized by treatment group using the FAS.

Qualitative data (e.g., gender, race, performance status) will be summarized by means of contingency tables for each treatment group, and quantitative data (e.g., age, body weight) will be summarized by appropriate descriptive statistics (mean, standard deviation, median, minimum and maximum) for each treatment group.

10.3 Treatments (study drug, concomitant therapies, compliance)

10.3.1 Study medication

Duration of study treatment exposure, cumulative dose and dose intensity will be summarized by treatment group. The number of patients with dose changes/interruptions will be presented by treatment group, along with reasons for the dose change.

10.3.2 Concomitant therapies

Concomitant medications and significant non-drug therapies taken concurrently with the study drugs will be listed and summarized by ATC class, preferred term and treatment arm by means of frequency counts and percentages.

These summaries will include medications starting on or after the start of study treatment or medications starting prior to the start of study treatment and continuing after the start of study treatment.

Any prior concomitant medications or significant non-drug therapies starting and ending prior to the start of study treatment will be listed.

The safety population will be used for all above-mentioned concomitant medication tables and listings.

10.4 Primary objective

The primary objective is to compare the SEGA response rate in patients with TSC-associated SEGA on RAD001 versus placebo.

10.4.1 Variable

The primary endpoint of this study, SEGA response rate, is defined as the proportion of patients with a SEGA response, where SEGA response is as defined in Section 7.5.2, and using data from the Independent Central Radiological Review of MRIs.

10.4.2 Statistical hypothesis, model, and method of analysis

The primary analysis will be a comparison of the SEGA response rates in the RAD001 and placebo arms using an exact Cochran-Mantel-Haenszel (CMH) (Agresti 2002) test at the one-sided 2.5% level, analyzed in the Full Analysis Set. The test will be stratified by the protocol stratification factor, that is, use of enzyme-inducing antiepileptic drugs (EIAED-users versus EIAED-non-users).

The statistical hypotheses are

$$H_0: RR_{RAD} \le RR_{PLB} \quad \text{versus} \quad H_1: RR_{RAD} > RR_{PLB},$$

where RR_{RAD} is the probability of SEGA response on RAD001 and RR_{PLB} is the probability of SEGA response on placebo.

SEGA response rates will be provided with exact 95% confidence intervals (Clopper and Pearson 1934). The analysis will be performed using data up to the data cut-off date of the trial, which will be six months after the last patient is randomized.

10.4.3 Handling of missing values/censoring/discontinuations

Patients with unknown SEGA response status will be treated as non-responders in the calculation of the SEGA response rate in the FAS at the end of the trial.

Other missing data will simply be noted as missing on appropriate tables/listings.

10.4.4 Supportive analyses

Potential effect of covariates (prognostic factors) will be investigated by using exact logistic regression. The objective of this analysis will be to explore the sensitivity of the statistical significance of treatment effect on SEGA response rate after adjusting for main prognostic factors. Odds ratios will be used as a measure of association between treatment and response, presented with exact 95% confidence limits. These analyses are considered as supportive.

The first model will include treatment group and the protocol stratification factor as covariates (the stratification factor is use of enzyme-inducing antiepileptic drugs [EIAED-users versus EIAED-non-users]). In a second model, the consistency of the treatment effect will be examined in the presence of possible prognostic factors measured at baseline, as described in Table 10-1, in addition to the stratification factor.

Table 10-1 Definition of potential prognostic factors

Prognostic Factor*	Definition	Model covariate definition
Parenchymal invasion	Deep, superficial or none<br>(Based on the deepest SEGA lesion)	DEEP (1=deep, 0=not deep)<br>SUPERFICIAL (1=superficial, 0=not superficial)
Bilateral lesion	At least one bilateral SEGA lesion	BILATERAL (1=yes, 0=no)
Inferior growth	At least one SEGA lesion growing inferiorly	INFERIOR (1=yes, 0=no)
Overall SEGA volume	SEGA volume at baseline	VOLUME (continuous)
Hydrocephalus	Presence of hydrocephalus at baseline	HYDRO (1=present, 0=absent)

* All factors as assessed at the Independent Central Radiological Review using the baseline MRI

The largest percentage change from baseline in the sum of volumes of target SEGA lesions will be presented graphically by means of a waterfall plot, shown separately for each treatment group.

The primary analysis of SEGA response rate will be repeated in the Per Protocol Set.

10.5 Secondary objectives

The secondary efficacy objectives were to compare RAD001 against placebo with respect to frequency of epileptiform events, time to SEGA progression and skin lesion response rate. In order to be able to make a claim on any of these three endpoints, a multiplicity adjustment will be implemented (see Section 10.5.4).

10.5.1 Frequency of epileptiform events

The frequency of epileptiform events will be obtained from the 24-hour video EEGs, as described in Section 7.5.4. The efficacy variable of main interest is the absolute change from baseline in the number of seizures per 24 hours obtained by video EEG, computed as described in Section 7.5.4. This variable will be compared between the RAD001 and placebo arms in the Full Analysis Set using rank analysis of covariance (rank ANCOVA, Stokes et al 2001), with baseline seizure frequency per 24 hours as a covariate. Rank ANCOVA is a non-parametric approach and was preferred to classical parametric ANCOVA because the data may not be even approximately normally distributed. However, in case it is approximately normal, it has been shown that rank ANCOVA does not lead to much loss of power versus parametric ANCOVA (Canover 1999). The model will be stratified by use of enzyme-inducing antiepileptic drugs (EIAED-users versus EIAED-non-users). The treatment effect will be declared significant if the p-value is less than or equal to 0.025. The mean number of seizures per 24 hours and the mean change from baseline in seizure frequency will be presented for each treatment group with 95% confidence intervals.

Within each treatment group, the proportion of patients receiving EIAEDs will be compared between baseline and at the time of the post-baseline video EEG.

10.5.2 Time to SEGA progression

Time to SEGA progression (TTSP) is determined using data from the Independent Central Radiological Review of MRIs. TTSP is defined as the time from the date of randomization to the date of the first documented SEGA progression, where SEGA progression is defined in Section 7.5.2. TTSP will be censored if SEGA progression is not observed before the first to occur out of (i) the cut-off date for the final analysis, or (ii) the date when a non-study systemic anti-SEGA therapy is started, or (iii) the date of death. The censoring date will be the date of the most recent MRI assessment before the first of any of these three events occurred.

If SEGA progression is observed after two or more missing or non-evaluable MRI assessments, then the date of SEGA progression will be censored at the latest occurring MRI. For SEGA progression observed after a single missing or non-evaluable MRI, the actual date of SEGA progression will be used.

TTSP will be compared between the RAD001 and placebo arms in the Full Analysis Set using a one-sided logrank test stratified by use of enzyme-inducing antiepileptic drugs (EIAED-users versus EIAED-non-users), and with a Type I error rate as described in Section 10.5.4 below. The TTSP distributions will be presented descriptively in the FAS using Kaplan-Meier curves. Summary statistics from the Kaplan-Meier distributions will be determined, including the median TTSP and the proportions of patients remaining progression-free at 6 and 12 months. These statistics will be given as point estimates with 95% confidence intervals.

The hazard ratio with a 95% confidence interval will be derived from the Cox proportional hazards model, stratified by use of EIAED (EIAED-users versus EIAED-non-users).

10.5.3 Skin lesion response rate

Skin lesion response rate is determined only among patients with at least one skin lesion at baseline, and is the proportion of this group of patients with a response (complete clinical response or partial response) on the Physician's Global Assessment of Clinical Condition (PGA), as described in Section 7.5.6.1.1. Once a patient begins laser treatment or receives local surgery to treat their skin lesions, any data obtained from that point onward will be excluded from the skin lesion response analysis.

Skin lesion response rate will be compared between the RAD001 and placebo arms in patients from the Full Analysis Set with at least one skin lesion at baseline, using a one-sided exact CMH test with a Type I error rate of 2.5%. The skin lesion response rate will be presented for each treatment arm, along with exact 95% confidence intervals.

10.5.4 Multiplicity adjustment for analysis of main secondary endpoints

Multiplicity will be controlled via a closed testing procedure. Thus, while the hypothesis tests for the three secondary endpoints described in Sections 10.5.1, Section 10.5.2 and Section 10.5.3 will all be carried out, the interpretation of the p-values will depend on the hierarchy used in the closed testing strategy. Based on clinical judgment, it was decided to rank the secondary efficacy endpoints in the order following order of importance: frequency of epileptiform events, followed by time to SEGA progression, followed by skin lesion response rate. Thus, the closed testing procedure is as follows:

1. Conduct primary analysis to compare RAD001 versus placebo on SEGA response rate using one-sided exact CMH test. If p>0.025 then STOP - otherwise, declare statistically significant benefit of RAD001 on SEGA response rate, and continue to next step.
2. Compare frequency of epileptiform events between RAD001 and placebo using rank ANCOVA. If p>0.025 then STOP - otherwise, declare statistically significant benefit of RAD001 on frequency of epileptiform events, and continue to next step.
3. Compare time to SEGA progression between RAD001 and placebo using a one-sided, stratified logrank test. If p>0.025 then STOP - otherwise, declare statistically significant benefit of RAD001 on time to SEGA progression, and continue to next step.
4. Compare skin lesion response rate between RAD001 and placebo using one-sided exact CMH test. If the treatment effect has p>0.025 then STOP - otherwise, declare statistically significant benefit of RAD001 on skin lesion response rate.

Thus for example, the first secondary endpoint, namely frequency of seizures, can only be formally declared statistically significant if its p-value is less than or equal to 0.025 and the primary endpoint was statistically significant. Similarly, time to SEGA progression can only be formally tested if both the primary analysis and frequency of epileptiform events were statistically significant. This approach ensures that the overall Type I error rate of the trial is maintained at 2.5% (one-sided).

10.5.5 Additional secondary efficacy analyses

Duration of SEGA response

Duration of SEGA response is defined as the time from the date of the first SEGA response until the date of the first SEGA progression, where SEGA response and SEGA progression are as defined in Section 7.5.2. Duration of SEGA response applies only to patients who achieve a SEGA response. Duration of SEGA response will be censored if SEGA progression is not observed before the first to occur out of (i) the cut-off date for the final analysis, or (ii) the date when a non-study systemic anti-SEGA therapy is started, or (iii) the date of death. The censoring date will be the date of the most recent MRI assessment before the first of any of these three events occurred.

Duration of SEGA response will be summarized for patients in the RAD001 treatment arm only. A Kaplan-Meier curve will be constructed, and the median response duration will be presented along with 95% confidence intervals. In addition, the Kaplan-Meier estimates with 95% confidence intervals at 3, 6 and 12 months will be summarized.

Time to SEGA response

Time to SEGA response is defined as the time from the date of randomization until the date of the first SEGA response, where SEGA response is as defined in Section 7.5.2. Time to SEGA response applies only to patients who achieve a SEGA response, i.e., patients in the analysis will have known times to response and there will be no censored times. Time to SEGA response will be summarized only in the RAD001 treatment arm. The median time to response will be presented along with a 95% confidence interval, and the proportions of SEGA responders who respond by 3 and 6 months will be provided.

Duration of skin lesion response

Duration of skin lesion response is defined as the time from the date of the first skin lesion response until the date of the first skin lesion progression, according to the Physician's Global Assessment (PGA), defined in Section 7.5.6.1.1. Duration of skin lesion response applies only to patients who achieve a skin lesion response. Duration of response will be censored if skin lesion progression is not observed before the first to occur out of (i) the cut-off date for the final analysis, or (ii) the date when a non-study systemic anti-SEGA therapy is started, or (iii) the date when laser treatment or local surgery to treat skin lesions is started, or (iv) the date of death. The censoring date will be the date of the most recent skin lesion assessment before the first of any of these four events occurred.

Duration of skin lesion response will be summarized for patients in the RAD001 treatment arm only. A Kaplan-Meier curve will be constructed, and the median response duration will be presented along with 95% confidence intervals. In addition, the Kaplan-Meier estimates with 95% confidence intervals at 3, 6 and 12 months will be summarized.

10.5.6 Safety

The assessment of safety will be based mainly on the frequency of adverse events and on the number of laboratory values that fall outside of pre-determined ranges. Other safety data (e.g., electrocardiogram, vital signs) will be considered as appropriate. All safety data will be listed.

For all safety analyses, the safety population will be used.

10.5.6.1 Adverse events

All adverse events (AEs) recorded during the study will be summarized. The incidence of adverse events will be summarized by body system, severity (based on CTCAE grades), type of adverse event, and relation to the study treatment. Deaths and SAEs will be listed by patient and type of adverse event.

Adverse events will be summarized by presenting the number and percentage of patients having any adverse event in each body system and having each individual adverse event. Any other information collected (e.g., severity or relatedness to study medication) will be listed as appropriate.

In addition, adverse events of related nature may be analyzed by categories regrouping the relevant preferred terms, as appropriate.

10.5.6.2 Laboratory abnormalities

All laboratory values will be converted into SI units and the severity grade calculated using appropriate common toxicity criteria for adverse events (CTCAE, version 3.0) unless otherwise indicated.

Renal function will be assessed using an age appropriate measure; either the Cockcroft-Gault formula (Cockcroft and Gault 1976) for patients 18 years and older or the Schwartz formula for patients up to age 18 (Schwartz et al 2009). The proportions of patients in each treatment group with severe renal impairment will be compared numerically. In addition, the proportion of patients with NCI CTCAE grade 3/4 serum creatinine will be determined for each treatment group and compared.

Endocrine parameters such as LH, FSH, testosterone levels (in males) and LH, FSH, estradiol levels (in females) will be summarized descriptively by gender for each treatment group at each time point.

A listing of laboratory values will be provided by laboratory parameter and by patient. The frequency of notable lab abnormalities will be displayed by parameter.

Similarly, the frequency of all laboratory abnormalities will be displayed by parameter and worst CTCAE grade experienced.

10.5.6.3 Growth Data

Growth data collected during the study will be summarized descriptively for each treatment group at each time point. These data consist of height, height velocity (difference of height over 1 year), weight, weight velocity (difference of weight over 1 year), the age at thelarche and menarche for girls, the age at adrenarche for boys as well as the Tanner stage assessment. In addition, based on height data collected during the study and published reference height information, the height standard deviation score (SDS, also called z-score) will be computed for a particular patient at each time point as:

(height - mean height for that age category) / SD of height for that age category

The same approach will be used to compute height velocity SDS, weight SDS and weight velocity SDS.

Descriptive statistics of these endpoints will be presented by time point and the z-scores will allow identification of potential outliers. Growth velocity during the trial will also be compared with growth velocity at baseline (if pre-baseline data are available).

10.5.6.4 Other safety data

Safety data from other tests (e.g., electrocardiogram or vital signs) will be listed, notable values flagged, and any other information collected will be listed as appropriate.

Any statistical tests performed to explore the data will be used only to highlight any interesting comparisons that may warrant further consideration.

10.5.7 Tolerability

Not applicable for this study.

10.5.8 Resource utilization

Not applicable for this study.

10.5.9 Pharmacokinetics

RAD001 blood concentrations in whole blood will be determined by a LC-MS method. Samples with concentration values below the lower limit of quantification (LLOQ) of the analytical method (0.3 ng/mL) will be labeled accordingly and will be excluded in data analysis. Missing values will be labeled accordingly.

Pre-dose trough concentrations of RAD001 (C_{min}) and concentration at 2 hours (± 30 mins, C_{2h}) post-dose will be summarized with descriptive statistics and graphically over time. Dose proportionality will be explored using a power model with the actual doses to fit log-trough level data. The potential relationships between C_{min} and efficacy/safety endpoints and between C_{2h} and efficacy/safety endpoints will be explored. A logistic regression model will be used for SEGA response with C_{min} or C_{2h}, with or without log-transformation, as an independent variable and stratified by the study strata. The relationship between RAD001 concentration and safety will be explored by modeling events of CTC grade 3 or above with a generalized estimating equation with C_{min} or C_{2h}, with or without log-transformation, as an independent variable and patients as random effects. Appropriate stratification and/or covariates may be applied in the model.

The measured RAD001 PK levels will be used in future analyses, along with RAD001 PK data from other studies (including, but not restricted to: [CRAD001C2239] (pancreatic neuroendocrine tumors); [CRAD001C2240] (renal cell carcinoma), [CRAD001C2244] (pediatric medulloblastoma), phase 1 studies [CRAD001C2101], [CRAD001C2102], [CRAD001C2104], [CRAD001C2106], [CRAD001C2107], [CRAD001C2108]). These analyses may use non-compartmental and population PK methods, with the goal of comparing the results from this protocol with appropriate reference populations (in other tumor types), and thus to characterize PK in the target population, with respect to exposure. The population PK analyses across studies will be reported separately, i.e., not as a part of the CSR for Study M2301.

Additional PK analysis for PK profile collected as part of Protocol Amendment 5

Blood samples for concentration determination of everolimus will be collected at pre-dose and at 0.5, 1, 2, 5, and 24 hours post-dose in patients at any ONE of the regular study visit days

during the open-label extension phase to estimate the impact of age, weight, BSA, and co-administration of CYP3A4/PgP inducers on the pharmacokinetics of everolimus (CL/F, C_{max}, T_{max}, C_{min}, AUC) in children based on the subset of patients who undergo additional PK collection in accordance with Amendment 5.

In order to monitor the number of patients in each age group, the age groups are defined as follow: <5, 5-<10, 10-<14, 14-<18, ≥18 years at time of pre-dose sampling. However, the definition of those age groups might be combined at the time of the analysis if needed.

The following PK parameters will be calculated using non-compartmental methods:

C_{max} – peak concentration during the 24 hours PK profile

T_{max} – time to Cmax

C_{min} – minimum concentration during the 24 hours PK profile, calculated as average of pre-dose and the 24 hour post-dose concentrations.

AUC_{tlast} – Area under the concentration-time curve from time zero to time of last quantifiable concentration in the 24 hours PK profile

$AUC_{0\text{-}24}$ – Area under the concentration-time curve during the 24 hours PK profile

CL/F – Dose/$AUC_{0\text{-}24}$

Samples not at steady-state will not be included in the analysis.

For each PK parameter except T_{max}, the ratios (and 90% CI) between the reference age group (≥ 18 years) and the other age groups will be estimated by a linear model with the log-transformed PK parameter as dependent variable, and age group as categorical variable.

The same model will be used to assess the impact of the other factors, including co-administration of CYP3A4/PgP inducers, weight and BSA (as categorical variable), restricting to the samples collected in patients aged less than 18 years. The model may be adjusted for other factors if appropriate. Categories of BSA and weight will be defined in the RAP.

If the distribution of age, weight and BSA does not allow estimation of ratios for comparisons of interest, for each factor separately, a regression model with the factor as a continuous variable (with transformation if appropriate) may be used to explore the relationship between PK parameters and these factors. The model may also be adjusted for other factors if appropriate.

Furthermore, a regression model including all the factors may be fitted to explore their joint effect, if appropriate considering data distribution and co-linearity between the factors. Population PK modeling may also be carried out to further explore their effects in pediatric population, in contrast to that in adult population.

All PK parameters including T_{max} will be summarized by categories of age, weight, BSA, and co-administration of CYP3A4/PgP enzyme inducers at time of the PK profile.

10.5.9.1 Biomarkers

The effect of RAD001 on biochemical tumor markers and on angiogenesis markers (e.g., VEGF, basic FGF, PLGF, soluble VEGF receptor1 and soluble VEGF receptor2) will be analyzed using summary statistics for raw data and changes from baseline and also using

longitudinal models. Relationships between ligands and corresponding soluble receptors will also be examined both relative to baseline and longitudinally.

10.6 Interim analysis

No interim analysis is planned for this study.

10.7 Sample size calculation

The primary analysis compares SEGA response rate between the two treatment arms using an exact Cochran-Mantel-Haenszel (CMH) test (Agresti 2002) in the Full Analysis Set. The randomization is unbalanced, with two patients allocated to RAD001 for every one patient allocated to placebo. In addition, the randomization is stratified by prior use of enzyme-inducing antiepileptic drugs (EIAED), categorized as users or non-users, and it is anticipated that 50% of patients will be EIAED users. It is planned to use a one-sided test and a 2.5% significance level. The SEGA response rate in the placebo arm is expected to be close to 0%, since there are no reported cases of spontaneous tumor regression in patients with SEGA. The SEGA response rate on RAD001 is anticipated to be at least 20%.

Sample size was determined using simulation (note that software providing sample sizes for exact CMH tests with unbalanced randomization is not readily available). The simulation approach involves randomly generating data according to the study assumptions for a large number of simulated trials, and then analyzing each trial using the exact CMH test. The proportion of times that the test is significant (i.e., has a one-sided p-value ≤ 0.025) gives the study power. Different sample sizes can be assessed, and by trial and error a sample size that guarantees a study power of at least 90% can be chosen. As a starting value, NQuery (V4.0) indicates that for analysis using Fisher's exact test (i.e., a different exact test, and one that does not take into account the stratification), a total of 99 patients would provide 93% power (2:1 randomization).

The power of the exact CMH test with 99 patients is shown in Table 10-2 below according to the study assumptions, notably a fixed overall SEGA response rate on RAD001 of 20%, and assuming a 1:1 ratio in the number of patients in Stratum 1 versus Stratum 2. The first row in the table assumes no treatment by stratum interaction; subsequent rows show increasing levels of interaction until the last row where response rate is 40% in Stratum 1 and 0% in Stratum 2. As can be seen, the power of the stratified test is highly robust to even the most extreme treatment by strata interaction. The sample size of 99 patients provides 93% power in all cases considered.

Table 10-2 Sensitivity of study power to treatment by stratum interaction, with balanced strata sizes

Patients in Stratum 1	SEGA response rate in RAD001 arm			Power*
	Stratum 1	Stratum 2	Overall	
50%	20%	20%	20%	93.40%
	25%	15%	20%	93.65%
	30%	10%	20%	93.65%
	35%	5%	20%	93.33%
	40%	0%	20%	93.49%

*Based on 10000 runs, and assumes 66/33 patients on RAD001/Placebo, Placebo response=0%, one-sided exact CMH test at 2.5% level.

The power of the exact CMH test is also highly robust to imbalance in the proportion of patients within each stratum. For example, Table 10-3 shows the study power for various degrees of treatment by strata interaction assuming a 3:1 ratio in the number of patients in Stratum 1 versus Stratum 2, again with 99 patients. It can be seen that 93% power is maintained in all cases of treatment by center interaction, and that power increases as the response rate in the smaller stratum increases.

Table 10-3 Sensitivity of study power to treatment by stratum interaction, with unbalanced strata sizes

Patients in Stratum 1	SEGA response rate in RAD001 arm			Power*
	Stratum 1	Stratum 2	Overall	
75%	26.67%	0%	20%	93.18%
	24%	8%	20%	93.32%
	20%	20%	20%	93.40%
	16%	32%	20%	93.51%
	12%	44%	20%	94.13%
	8%	56%	20%	95.57%
	4%	68%	20%	97.46%
	0%	80%	20%	98.77%

*Based on 10000 runs, and assumes 66/33 patients on RAD001/Placebo, Placebo response=0%, one-sided exact CMH test at 2.5% level.

Table 10-4 shows the relationship between study power and the size of the treatment effect. The power is rather sensitive to even small changes in the RAD001 response rate; however, with 99 patients the power is at least 86% if the true response rate is greater than 18%, and at least 97% if it is greater than 22%. The required increase in sample size to provide sufficient power to detect smaller treatment effects was considered unnecessary (e.g., if the true response rate on RAD001 was 14%, sample size would need to increase by about 40% in order to ensure 90% power).

Table 10-4 Sensitivity of study power to size of treatment effect

SEGA response rate		Power* with 99 patients	Number of patients to ensure ≥90% power*
RAD001	Placebo		
14%	0%	59.05%	138
16%	0%	75.23%	120
18%	0%	86.39%	105
20%	0%	93.40%	95
22%	0%	97.11%	87
24%	0%	98.85%	78
26%	0%	99.55%	72

*Based on 10000 runs, and assumes 2:1 ratio for RAD001:Placebo, 1:1 ratio for Stratum 1:Stratum 2, one-sided exact CMH test at 2.5% level.

Taking all these simulation results into account, the total sample size was chosen to be 99 patients, with 66 randomized to the RAD001 arm and 33 randomized to placebo.

11 Administrative procedures

Regulatory and ethical compliance

This clinical study was designed and shall be implemented and reported in accordance with the protocol, the ICH Harmonized Tripartite Guidelines for Good Clinical Practice, with applicable local regulations (including European Directive 2001/20/EC and US Code of Federal Regulations Title 21), and with the ethical principles laid down in the Declaration of Helsinki.

Responsibilities of the investigator and IRB/IEC/REB

The protocol and the proposed informed consent form must be reviewed and approved by a properly constituted Institutional Review Board/Independent Ethics Committee/Research Ethics Board (IRB/IEC/REB) before study start. A signed and dated statement that the protocol and informed consent have been approved by the IRB/IEC/REB must be given to Novartis before study initiation. Prior to study start, the investigator is required to sign a protocol signature page confirming his/her agreement to conduct the study in accordance with these documents and all of the instructions and procedures found in this protocol and to give access to all relevant data and records to Novartis monitors, auditors, Novartis Clinical Quality Assurance representatives, designated agents of Novartis, IRBs/IECs/REBs and regulatory authorities as required.

Informed consent

Eligible patients may only be included in the study after providing written (witnessed, where required by law or regulation), IRB/IEC/REB-approved informed consent, or, if incapable of doing so, after such consent has been provided by a legally acceptable representative of the patient. Informed consent must be obtained before conducting any study-specific procedures (i.e., all of the procedures described in the protocol). In cases where the subject's legally

acceptable representative gives consent, the subject (e.g., minors, patients with severe dementia), should be informed about the trial to the extent compatible with the subject's understanding and if capable, the subject should assent, sign and personally date the written informed consent. The process of obtaining informed consent should be documented in the patient source documents. In emergency situations when prior consent of the subject is not possible and the subject's legally acceptable representative is not available, enrollment of the subject should require measures described in the protocol with documented favorable opinion of the IRB/IEC/REB. The subject or the subject's legally appointed representative should be informed about the trial as soon as possible and consent to continue and other consent as appropriate should be requested.

A proposed informed consent form that complies with the ICH GCP guideline and regulatory requirements and is considered appropriate for this study is provided to each site. Any changes to the proposed consent form suggested by the investigator must be agreed to by Novartis before submission to the IRB/IEC/REB, and a copy of the approved version must be provided to the Novartis monitor after IRB/IEC/REB approval.

Amendments to the protocol

Any change or addition to the protocol can only be made in a written protocol amendment that must be approved by Novartis, Health Authorities where required, and the IRB/IEC/REB. Only amendments that are required for patient safety may be implemented prior to IRB/IEC/REB approval. Notwithstanding the need for approval of formal protocol amendments, the investigator is expected to take any immediate action required for the safety of any patient included in this study, even if this action represents a deviation from the protocol. In such cases, Novartis should be notified of this action and the IRB/IEC/REB at the study site should be informed within 10 working days.

Discontinuation of the study

Novartis reserves the right to discontinue this study under the conditions specified in the clinical trial agreement.

Study drug supply and re-supply, storage, and tracking/drug accountability

Study drugs must be received by a designated person at the study site, handled and stored safely and properly, and kept in a secured location to which only the investigator and designated assistants have access. Upon receipt, the RAD001 should be stored according to the instructions specified on the drug labels. Clinical supplies are to be dispensed only in accordance with the protocol.

Medication labels will be in the local language and comply with the legal requirements of each country. They will include storage conditions for the drug and the medication number but no information about the patient.

The investigator must maintain an accurate record of the shipment and dispensing of study drug in a drug accountability ledger. Drug accountability will be noted by the field monitor during site visits and at the completion of the trial. Patients will return all unused study drug

and packaging at each dispensing visit and at the end of the study or at the time of study drug discontinuation.

At the conclusion of the study, and, as appropriate during the course of the study, the investigator will return all used and unused study drug, packaging, drug labels, and a copy of the completed drug accountability ledger to the Novartis monitor or to the Novartis address provided in the investigator folder at each site.

12 Protocol adherence

Investigators ascertain they will apply due diligence to avoid protocol deviations. Under no circumstances should the investigator contact Novartis or its agents, if any, monitoring the trial to request approval of a protocol deviation, as no authorized deviations are permitted. If the investigator feels a protocol deviation would improve the conduct of the study this must be considered a protocol amendment, and unless such an amendment is agreed upon by Novartis and approved by the IRB/IEC/REB it cannot be implemented. All significant protocol deviations will be recorded and reported in the CSR.

13 References (available upon request)

Agresti A, Categorical Data Analysis (Second Edition), Wiley, New York, 2002.

Astrinidis A, Henske EP (2005) Tuberous sclerosis complex: linking growth and energy signaling pathways in human disease. Oncogene; 24: 7475-7481.

Atkins MB, Hildalgo M, Stadler WM et al (2004) Randomized phase II study of multiple dose levels of CCI-779, a novel mammalian target of rapamycin kinase inhibitor, in patients with advanced refractory renal cell carcinoma. J Clin Oncology; 22(5): 909-918.

Bjornsti MA and Houghton PJ (2004) The TOR pathway. A target for cancer chemotherapy. Nature Reviews Cancer; 4:335-338.

Boulay, Rudloff J, Ye J, Zumstein-Mecker S, O'Reilly T, Evans DB, Chen S, Lane HA (2005) Dual inhibition of mTOR and estrogen receptor signaling in vitro induces cell death in models of breast cancer. Clin Cancer Res; 11: 5319-28.

Brusasco V, Crapo R, Viegi G (2005) Coming together: the ATS/ERS consensus on clinical pulmonary function testing. Eur Res *J*; 26 (1): 1-2.

Canover WJ, Practical Nonparametric Statistics (Third Edition), Wiley, New York, 1999.

Chan JA, Zhang H, Roberts PS (2004) Pathogenesis of tuberous sclerosis subependymal giant cell astrocytomas: biallelic inactivation of TSC1 or TSC2 leads to mTOR activation. *J* Neuropathol Exp Neurol, 63(12): 1236-42

Clopper C, Pearson S (1934) The use of confidence or fiducial limits illustrated in the case of the binomial. Biometrika 26: 404-413

Cockcroft DW, Gault MH et al (1976). Prediction of creatinine clearance from serum creatinine. Nephron; 16: 31-41.

Cohen HT, McGovern FJ, et al (2005) Renal-cell carcinoma. N Engl J Med, 353:2477-90.

Cuccia V, Zuccaro G, Sosa F, et al (2003) Subependymal giant cell astrocytoma in children with tuberous sclerosis. Childs Nerv Syst Apr; 19(4): 232-43.

DuBois D, DuBois EF (1916) A formula to estimate the approximate surface area if height and weight be known. Arch Intern Medicine; 17: 863-71.

Duvic M, Martin AG, Kim Y, Olsen E, Wood GS, Crowley CA, Yocum RC (2001) Phase 2 and 3 clinical trial or oral bexarotene (Targretin capsules) for the treatment of refractory or persistent early-stage cutaneous T-cell lymphoma. Arch Dermatol; 137: 581-593.

Erbayat-Altay E, Zeng L-H, Xu L, Gutmann DH, Wong M (2007). The natural history and treatment of epilepsy in a murine model of tuberous sclerosis. Epilepsia, in press.

Fouladi M, Laningham F, Jianrong W, et al (2007) Phase I Study of Everolimus in Pediatric Patients with Refractory Solid Tumors. Journal of Clinical Oncology*;* 30: 4806-4812.

Franz DN, Brody A, Meyer C, et al. (2001) Mutational and radiographic analysis of pulmonary disease consistent with lymphangioleiomyomatosis and micronodular pneumocyte hyperplasia in women with tuberous sclerosis. Am J Respir Crit Care Med; 164: 661-8.

Franz DN (2007). Tuberous Sclerosis. http://www.emedicine.com/neuro/topic386.htm. Last updated February 14, 2007. Accessed May 4, 2007.

Gomez MR, Sampson JR, Whittemore VH (eds) (1999): Tuberous Sclerosis Complex. Oxford: Oxford University Press.

Heald P, Mehlmauer M, Martin AG, Crowley CA, Yocum RC, Reich SD (2003) Topical bexarotene therapy for patients with refractory or persistent early-stage cutaneous T-cell lymphoma: results of the phase III clinical trial. J Am Acad Dermatol; 49: 801-815.

Hoofnagle J (2009). Reactivation of Hepatitis B. Hepatology 49: S156-S165.

Hyman MH; Whittemore VH (2000) National Institutes of Health consensus conference: tuberous sclerosis complex. Arch Neurol. 2000; **57**: 662-5

Inoki K, Corradetti MN, Guan K-L (2005) Dysregulation of the TSC-mTOR pathway in human disease. Nature Genetics; 37: 19-24.

Kenerson H, Dudon TA, Yeung RS (2005) Effects of rapamycin in the Eker rat model of tuberous sclerosis complex. Pediatric Res; 57: 67-75.

Krishna G, Moton A, Lei Ma L, Savant I, Martinho M, Seiberling M, McLeod J. Effects of oral posaconazole on the pharmacokinetic properties of oral and intravenous midazolam: A phase I, randomized, open-label, crossover study in healthy volunteers. Clinical Therapeutics, 2009; 31: 286-98.

Kwiatkowski DJ, Manning BD (2005) Tuberous Sclerosis: a GAP at the crossroads of multiple signaling pathways. Human Molecular Genetics ; 14: R1-R8.

Marshall WA, Tanner JM (1969) Variations in pattern of pubertal changes in girls. Arch Dis Child; 44:291-303.

Marshall WA, Tanner JM (1970) Variations in pattern of pubertal changes in boys. Arch Dis Child; 45:13-23.

Matsumura H, Takimoto H, Shimada N, et al. (1998) Glioblastoma following radiotherapy in a patient with tuberous sclerosis. Neurol Med Chir (Tokyo); 38: 287-91.

Mayr NA, Yuh WT, Taoka T, et al (2006), Serial therapy-induced changes in tumor shape in cervical cancer and their impact on assessing tumor volume and treatment response. Am J Roentgenol; 187:65-72.

Miller A.B, Hoogstraten B., Staquet M. et al (1981) Reporting results of cancer treatment. Cancer; 47:207-214.

O'Reilly TM, Wood JM, Littlewood-Evans A, Boulay A, Schnell CR, Patrizia S, Maira M, Martiny-Baron G, Lane HA (2005). Differential anti-vascular effects of mTOR or VEGFR pathway inhibition: a rational basis for combining RAD001 and PTK787/ZK222584. Proc Am Assoc Cancer Res; 46:715. Abstract 3038.

Onda H, Lueck A, Marks PW, Warren HB, Kwiatkowski DJ (1999) Tsc2(+/-) mice develop tumors in multiple sites that express gelsolin and are influenced by genetic background. J Clin Invest; 104: 687-95.

Partridge SC, Gibbs JE, Lu Y, Esserman LJ, et al. (2005) MRI measurements of breast tumor volume predict response to neoadjuvant chemotherapy and recurrence-free survival. Am J Roentgen; 184: 1774-81.

Report DMPK (CH) R00-2214. RAD001: Dose-dependent brain penetration in rat. Novartis Pharma AG, Basel, Switzerland. 04-Oct-2000.

Report RD-2001-00852. RAD001: Effects on endothelial and fibroblast cell proliferation. Novartis Pharma AG, Basel, Switzerland. 11-Oct-2001.

Report RD-2002-03252. RAD001: Comparison of the antiproliferative activity of RAD001 with activation of the PTEN/PI3 kinase/Akt/mTOR pathway in tumor cell lines. Novartis Pharma AG, Basel, Switzerland. 30-Oct-2002

Roach ES, Gomez MR, Northrup H. Tuberous sclerosis complex consensus conference: revised clinical diagnostic criteria. J Child Neurol 1998; **13**: 624-8.

Schwartz G, Munoz A, Schneider M, et al (2009) New equations to estimate GFR in children with CKD. J. Am. Soc. Nephrol. **20:** 629-637.

Shepherd, CW et al (1991) Subependymal giant cell astrocytoma: A clinical, pathological and flow cytometric study. Neurosurgery*;* 28: 864-868.

Sinson G, Sutton LN, Yachnis AT, et al (1994) Subependymal giant cell astrocytomas in children. Pediatr Neurosurg; 20: 233-9.

Stokes ME, Davis CS, Koch GG. Categorical Data Analysis using the SAS System (Second Edition), SAS Institute, Cary, NC, 2001.

Tabernero J. Rojo F, Casado E. et al (2005) A Phase 1 study with tumor molecular pharmacodynamic evaluation of dose and schedule of the oral mTOR inhibitor everolimus (RAD001) in patients with advanced solid tumors. [abstract]. J Clin Oncol: 23 16S: A3007.

Tabernero J, Rojo F, Calvo E, et al (2007). Dose- and schedule-dependent inhibition of the mTOR pathway with everolimus: A phase I tumor pharmacodynamic study in patients with advanced tumors. J Clin Oncol 2008: in press.

Therasse P, Arbuck SG, Eisenhauer EA et al (2000) New guidelines to evaluate the response to treatment in solid tumors. J Natl Cancer Inst; 92: 205-16.

Torres OA, Roach ES, Delgado MR, et al (1998) Early diagnosis of subependymal giant cell astrocytoma in patients with tuberous sclerosis. J Child Neurol; 13: 173-7.

Twombly R (2006) Criticism of Tumor Response Criteria Raises Trial Design Questions. JNCI; 98: 232.

Uhlmann EJ, Wong M, Baldwin RL, Bajenaru ML, Onda H, Kwiatkowski DJ, Yamada K, Gutmann DH (2002) Astrocyte-specific TSC1 conditional knockout mice exhibit abnormal neuronal organization and seizures. Ann Neurol; 52: 285-96.

Yates JRW (2006) Tuberous sclerosis. Europ J Hum Gen; 14:1065-73
